# Probable presence of an ubiquitous cryptic mitochondrial gene on the antisense strand of the cytochrome oxidase I gene

**DOI:** 10.1186/1745-6150-6-56

**Published:** 2011-10-24

**Authors:** Eric Faure, Luis Delaye, Sandra Tribolo, Anthony Levasseur, Hervé Seligmann, Roxane-Marie Barthélémy

**Affiliations:** 1 LATP, CNRS-UMR 6632, IFR 48 Infectiopôle, Evolution biologique et modélisation, case 5, Université de Provence, Place Victor Hugo, 13331 Marseille cedex 3, France; 2 Departamento de Ingeniería Genética, CINVESTAV, Irapuato Km 9.6 Libramiento Norte Carr. Irapuato-León 36821 Irapuato Gto, México; 3 Biovays SAS, Université de Provence, 3, place Victor Hugo, 13331 Marseille cedex 3, France; 4 INRA, UMR1163 de Biotechnologie des Champignons Filamenteux, IFR86-BAIM. Aix-Marseille Universités, ESIL, 163 avenue de Luminy, CP 925, 13288 Marseille Cedex 09, France; 5 Aix-Marseille Univ, UMR1163 BCF, 163 avenue de Luminy, CP925, 13288 Marseille Cedex 09, France; 6 National Collections of Natural History, The Hebrew University of Jerusalem, 91904 Jerusalem, Israel; 7 Department of Life Sciences, Ben Gurion University, 84105 Beer Sheva, Israel

**Keywords:** Mitochondrial DNA, *cox-1 *gene, ubiquitous gene, overprinting, genome evolution, janolog

## Abstract

**Background:**

Mitochondria mediate most of the energy production that occurs in the majority of eukaryotic organisms. These subcellular organelles contain a genome that differs from the nuclear genome and is referred to as mitochondrial DNA (mtDNA). Despite a disparity in gene content, all mtDNAs encode at least two components of the mitochondrial electron transport chain, including cytochrome *c *oxidase I (Cox1).

**Presentation of the hypothesis:**

A positionally conserved ORF has been found on the complementary strand of the *cox1 *genes of both eukaryotic mitochondria (protist, plant, fungal and animal) and alpha-proteobacteria. This putative gene has been named *gau *for gene antisense ubiquitous in mtDNAs. The length of the deduced protein is approximately 100 amino acids. In vertebrates, several stop codons have been found in the mt *gau *region, and potentially functional *gau *regions have been found in nuclear genomes. However, a recent bioinformatics study showed that several hypothetical overlapping mt genes could be predicted, including *gau; *this involves the possible import of the cytosolic AGR tRNA into the mitochondria and/or the expression of mt antisense tRNAs with anticodons recognizing AGR codons according to an alternative genetic code that is induced by the presence of suppressor tRNAs. Despite an evolutionary distance of at least 1.5 to 2.0 billion years, the deduced Gau proteins share some conserved amino acid signatures and structure, which suggests a possible conserved function. Moreover, BLAST analysis identified rare, sense-oriented ESTs with poly(A) tails that include the entire *gau *region. Immunohistochemical analyses using an anti-Gau monoclonal antibody revealed strict co-localization of Gau proteins and a mitochondrial marker.

**Testing the hypothesis:**

This hypothesis could be tested by purifying the *gau *gene product and determining its sequence. Cell biological experiments are needed to determine the physiological role of this protein.

**Implications of the hypothesis:**

Studies of the *gau *ORF will shed light on the origin of novel genes and their functions in organelles and could also have medical implications for human diseases that are caused by mitochondrial dysfunction. Moreover, this strengthens evidence for mitochondrial genes coded according to an overlapping genetic code.

## Background

Mitochondria play a central role in eukaryotic metabolism, apoptosis, disease and aging [1]. Oxidative phosphorylation, which is essential for the production of ATP and for a variety of other biochemical functions, occurs in mitochondria. These essential subcellular organelles contain mitochondrial DNA (mtDNA), which is an extrachromosomal genetic element. The mitochondrial genes are arranged compactly and generally have no introns and few intergenic nucleotides. In some mtDNAs (e.g., nematodes and annelids), all genes are transcribed in the same direction, whereas, in others, both strands encode genes. With few exceptions, metazoan mtDNAs are covalently closed-circular molecules that autonomously replicate and transcribe within the organellar matrix. The mitochondrial gene content is highly variable across eukaryotes. The number of mitochondrial protein genes is believed to vary from 3 to 67, while the tRNA gene content varies from 0 to 27 [2]. Typical triploblastic animal mtDNAs contain genes that encode the large and small subunit ribosomal RNAs, 22 transfer RNAs (tRNAs), and 13 proteins that are all components of the oxidative phosphorylation process: ATPase subunits 6 and 8 (ATP6 and 8), cytochrome *b *(Cyt b), cytochrome *c *oxidase subunits 1-3 (Cox1-3) and NADH dehydrogenase subunits 1-6 and 4L (ND1-6 and 4L). In some species that are missing mitochondrial genes, such as *atp6 *and *atp8 *[3], these genes are either encoded in the nucleus (as it has been shown for the *atp6 *gene of a non-metazoan (*Chlamydomonas reinhardti*) [4]) or have been lost, which implies that their function has become dispensable or has been assumed by other proteins.

In view to find putative mitochondrial genes in alternative reading frames on both coding and "presupposed" non-coding strands, numerous complete mtDNA genomes have been analyzed. This search has led to the observation of an unknown, positionally conserved open reading frame (ORF) on the complementary strand of eukaryotic *cox1 *genes.

An independent method was designed to detect previously unknown overlapping protein coding mt genes using microevolutionary comparisons of primate mitochondrial gene sequences in view to find a number of such genes in various alternative frames, including a sequence corresponding to the above mentioned ORF [5]. The study of this ORF constitutes the aim of this article.

## Presentation of the hypothesis

**Figure 1:**
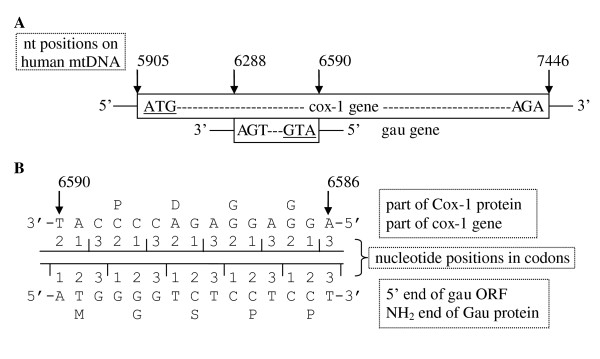
**The open reading frames for the *cox1 *and *gau *genes are shown in more detail**. (A) The *gau *ORF is oriented opposite to *cox1*. The potential start and stop codons are underlined and italicized, respectively. (B) The respective nucleotide positions in the codons at the 5' end of the *gau *ORF and in the corresponding part of the *cox1 *gene. The numbers indicate the nucleotide residue in the human mtDNA (NC_001807).

**Figure 2:**
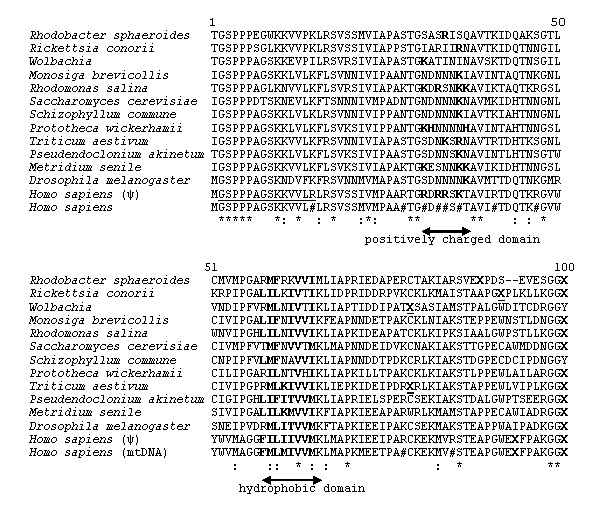
**Sequences of Gau proteins from representative taxa of alpha-proteobacteria and eukaryotes**. For each nuclear or mtDNA sequence, the specific genetic code has been used. Due to the AGG and AGA stop codons in the mitochondrial vertebrate genetic code (*Homo sapiens)*, the Gau protein sequence has been deduced from a *cox1 *nuclear pseudogene. Taxa with accession numbers, alpha-proteobacteria: *Rhodobacter sphaeroides *(CP000661.1) (the *gau *region is complementary to the sequence from nucleotide position 557002 to 556700), *Rickettsia conorii *(NC_003103), *Wolbachia *endosymbiont of *Drosophila melanogaster *(AE017196); protists: *Monosiga brevicollis *(NC_004309), *Rhodomonas salina *(NC_002572); *Prototheca wickerhamii *(NC_001613); Viridiplantae: *Triticum aestivum *(NC_007579), *Pseudendoclonium akinetum *(NC_005926); fungi: *Saccharomyces cereviseae *(NC_001224), *Schizophyllum commune *(NC_003049); metazoa: *Metridium senile *(NC_000933), *Drosophila melanogaster *(NC_001709), *Homo sapiens *(NC_001807) (human mt-Gau deduced protein matches from base positions 6288 to 6590) and a human nuclear pseudogene (*Homo sapiens *(ψ)) (ensemble.org, chromosome: NC_000001.9, *gau *region matches from the complementary to the sequence from nucleotide positions 557002 to 556700). X, UAA or UAG stop codons; X, UGA stop codon; #, AGA or AGG stop codons; *, identical amino acids;:, similar amino acids. All sequence alignments were performed using ClustalW (http://www.ebi.ac.uk/Tools/clustalw2/index.html). The monoclonal antibody was raised against a synthetic peptide that was derived from the underlined region in the human nuclear pseudogene sequence. Positive and hydrophobic amino acids are in bold letters in the positively charged and hydrophobic domains, respectively.

To identify the novel putative mtDNA genes, numerous complete mtDNA genomes have been translated in all six frames, and all of the putative ORFs in unconventional frames have been studied. Conceptual translations using the corresponding translation table have been made using the program "VirtualRibosome" [6]. The presence of putative ORFs in the nucleotide sequence was determined using "NCBI ORF finder" [7]. Among these results is a relatively long alternative ORF on the complementary strand of the *cox1 *genes or, more precisely, in the region corresponding to the second half of the NH_2_-terminal domain (Figure 1). Genes can overlap on the same strand or on the opposite strand. Moreover, despite stop codons in sequences, a relatively well-conserved nucleotide region has been found in all of the complete mtDNAs analyzed. Its position versus the *cox1 *gene justifies the name *gau *(*g*ene *a*ntisense *u*biquitous in mitochondrial genomes). The deduced Gau protein sequence is relatively well conserved in protist, fungal, plant and animal mtDNAs and in members of the alpha-proteobacteria taxa (rickettsiales, Rhizobacteria, rhodobacterales), the presumed ancestors of mitochondria [8] (Figure 2). In other bacterial taxa, such as proteobacteria other than alpha-proteobacteria, Chloroflexi, Cyanobacteria, Firmicutes and Actinobacteria, a region with a lower degree of similarity to the Gau protein can also be found after translation of the complementary strand of the genes encoding members of the cytochrome *c *oxidase subunit I-like SCOP superfamily [9].

The translational start and stop codons of the *gau *ORF are unknown due to alternative initiation codons [10], RNA editing [11] and suppressor tRNAs [12], which are well known in mtDNAs [13,14]. Therefore, it is difficult to predict the exact size of the *gau *coding region. We hypothesize that it could be exactly 100 codons long (see discussion).

### Are there functional copies of *gau *in the nuclear genomes of vertebrates?

As in other taxa, in vertebrate mtDNAs, the sequences of the *gau *regions are highly conserved but they are punctuated by numerous AGR (R for purines) stop codons. To determine regions that are homologous to the *gau *genes but free of stop codons, various complete or almost complete nuclear vertebrate genome sequences were screened. Alignments of both Gau deduced proteins and *cox1 *regions revealed some interesting features (Table 1 and Additional file 1). For instance, in both *Pan troglodytes *(Ch2a) and *Pongo pygmaeus *(Ch2a), the two flanking regions of the *gau-*like genes have a very low level of similarity to the *cox1 *genes. This suggests that in *cox1 *pseudogenes, negative selection has only acted to conserve the *gau *ORFs. This hypothesis is reinforced by the fact that in the nuclear *gau *regions, the number of stop codons is lower than in the mitochondrial sequences that were translated using the standard genetic code (Additional file 1). Surprisingly, in vertebrates, nuclear *gau *regions were found in introns of various genes (Table 1).

**Table 1:** Characteristics of the vertebrate nuclear regions that exhibit the greatest level of homology with the *gau *region

Vertebrate species	Chrom. number	Chromosome position of the *gau *ORF (101 codons)	Sequence of the possible start codon	Positions of the stop codons	% identities of nuclear nucleic acid sequences/mt-*gau *region Number of indel(s)/mt-*gau *region	% identities of nuclear nucleic acid sequence/mt-*cox1 *gene Number of indel(s)/mt-*cox1 *gene	Transcriptional data of the *gau *nuclear region according to Ensembl.org
Human *Homo sapiens*	1	557002-556700	ATG	94 (TAG) 101 (TAG)	97.0% no indel	98.6% 1 indel	In the cDNA (Acc. n° ENST00000391564) of an unprocessed pseudogene (no protein product). Orientation of *gau *vs this transcript: -
Human *Homo sapiens*	14	32023072-32023374	ATA	94 (TAG) 101 (TAG)	90.8% no indel	67.2% 29 indels	In the intron of the cDNA (Acc. n° ENST00000280979) encoding the Protein Kinase A-anchoring protein 6) (PRKA6). Orientation vs transcript: +
Chimpanzee *Pan troglodytes*	2a	51807478-51807780	ATA	52(TGA) 94(TAG) 101 (TAA)	82.5% no indel	62.7% 30 indels	In the intron of 5 cDNAs of the same gene (Acc. n° ENSPTRT00000012096, ENSPTRT00000058923, ENSPTRT00000058924, ENSPTRT00000064068, ENSPTRT00000066428, ENSPTRT00000067456) encoding unknown protein. Orientation of *gau *vs these transcripts: -
Chimpanzee *Pan troglodytes*	8	47845028-47844726	ATA	92(TGA) 94(TAA) 101 (TAG)	88.8% no indel	80.9% 6 indels	Non transcripted region
Orangutan *Pongo pygmaeus*	2a	60553759-60553457	ATA	101 (TAA)	83.5% no indel	64.9% 37 indels	In the intron of the cDNA (Acc. n° ENSPPYT00000014418) encoding the Neurexin-1-alpha Precursor (Neurexin I-alpha). Orientation of *gau *vs this transcript: -
Macaque *Macaca mulatta*	1	108934996-108934700	ATA	no	95.2% no indel	95.2% no indel (partial sequence)	Non transcripted region
Macaque *Macaca mulatta*	2	12317948-123179188	ATA	no	96.4%	77.5% 19 indels (partial sequence)	Non transcripted region
Macaque *Macaca mulatta*	6(a)	30942119-30941823	ATA	no	95.7% no indel	68.9% indels (partial sequence)	Non transcripted region
Macaque *Macaca mulatta*	6(b)	50452034-50451738	ATA	no	97.0%	67.3% 31 indels (partial sequence)	In the intron of two cDNAs of the same gene (Acc. n° ENSMMUT00000002475, ENSMMUT00000002476) encoding the Integrin alpha-1 Precursor. Orientation of *gau *vs these transcripts: -
Horse *Equus caballus*	27	5204837-5205139	ATT	50 (TGA)	87.8% no indel	88.9% no indel	Non transcripted region
Dog *Canis familiaris*	16	9457729-9458031	ATG	no	83.5% no indel	74.7% 15 indels (partial sequence)	Non transcripted region
Cow *Bos taurus*	10	4584422-4584126	ATA	no	88.1%	87.6% 4 indels	In the intron of a cDNA (Acc. n° ENSBTAT00000020753) encoding an unknown protein. Orientation of *gau *vs this transcript: +
Mouse *Mus musculus*	2	22444482-22444784	ATT	50(TGA) 79(TGA) 101 (TAG)	97.7% no indel	97.6% no indel	In the intron of the cDNA (Acc. n° ENSMUST00000044749) encoding the Myosin IIIA. Orientation of *gau *vs this transcript: +

Sequences extracted from ensemble.org. The deduced amino acid sequences are shown in Figure 2.

These analyses could be compatible with a functional nuclear ORF for Gau, whose product would be imported into the mitochondria.

### Mitochondrial expression and antisense tRNAs

The possible *in situ *expression of the vertebrate mitochondrial *gau *ORF and the synthesis of the corresponding protein product cannot be ruled out despite the presence of stop codons within the *gau *region and other putative overlapping mitochondrial genes. Importing cytosolic tRNAs [15-21] with anticodons that recognize AGR codons and bear the cognate amino acid arginine, according to the standard genetic code used in vertebrate nuclear chromosomes, could allow mitochondrial expression of these mitochondrial genes [5]. Additionally, it was shown that both DNA strands are transcribed in their entirety [22]. Hence, it is very likely that RNA that corresponds to the putative *gau *gene is produced in mitochondria. Accordingly, RNA corresponding to two other putative mitochondrial antisense overlapping genes on the complementary strand of mammalian ATP6 and ATP8 has been previously detected and shown to be associated with low resource availability ([23], as presented by figure 6 in reference [5]).

A further independent mechanism could enable mitochondrial expression of the mitochondrial *gau *region despite the presence of stop codons. RNA corresponding to the complementary (antisense) sequence of primate sense tRNAs has a number of properties beyond that of forming cloverleaf secondary structures, which suggests activity in protein synthesis [24]. For example, the usage frequencies of amino acid cognates in regular primate mitochondrial ORFs coevolve with various antisense tRNA properties that are required for proper activity in translation [24]. These results were also confirmed by the association between the pathogenic effects of human polymorphisms in tRNA genes and the cloverleaf formation of the antisense tRNA sequences [25]. Pathogenic mutations, when compared to neutral polymorphisms, decrease cloverleaf formation in antisense tRNAs that are predicted to be active in translation according to other properties and analyses [24]. However, the opposite occurs (pathogenic mutants increase cloverleaf formation compared to regular polymorphisms) for antisense tRNAs that are not expected to function in translation (figure 3 in [25]). The finding that was most relevant to the expression of antisense ORFs, which include stop codons such as in *gau*, is that the anticodons of some antisense mitochondrial tRNAs correspond to mitochondrial stop codons, also called antitermination (antisense) tRNAs [26]. Several patterns suggest that regular sense protein synthesis is adapted for proper function in the presence of these antitermination tRNAs. One of these patterns is the negative coevolution between the formation of the antisense antitermination tRNA cloverleaf and the regular main frame usage in mitochondrial genes with stop codons corresponding to the tRNA anticodons. These results themselves are evidence for translational activity by antisense tRNAs [26]. In addition, comparative analyses of primate mitochondrial genomes show that predicted antisense antitermination tRNA properties coevolve positively with putative mitochondrial overlapping genes (including *gau*) whose expression necessitates the suppressor/antitermination antisense tRNAs [5]. Coevolution between the predicted primate mitochondrial antisense antitermination tRNAs and the putative mitochondrial off-frame overlapping genes requires stop codon suppression for their expression, which suggests that proteins corresponding to Gau and other mitochondrial genes may be synthesized *in situ *in the mitochondria. Notably, these mechanisms enable potential mitochondrial expression of *gau *in association with antisense antitermination tRNAs or by importing cytosolic tRNAs. However, these do not imply that the negative selection of stop codon usage within the *gau *region does not occur, especially in species such as *Homo sapiens*, in which no antisense antitermination tRNA is predicted to exist [5,26]. Low stop codon numbers would cause *gau *expression to be independent of the induction of cytosolic tRNA import and/or antisense tRNAs, which would enable the independent regulation of expression of the various putative overlapping genes according to the number of stop codons.

**Figure 3:**
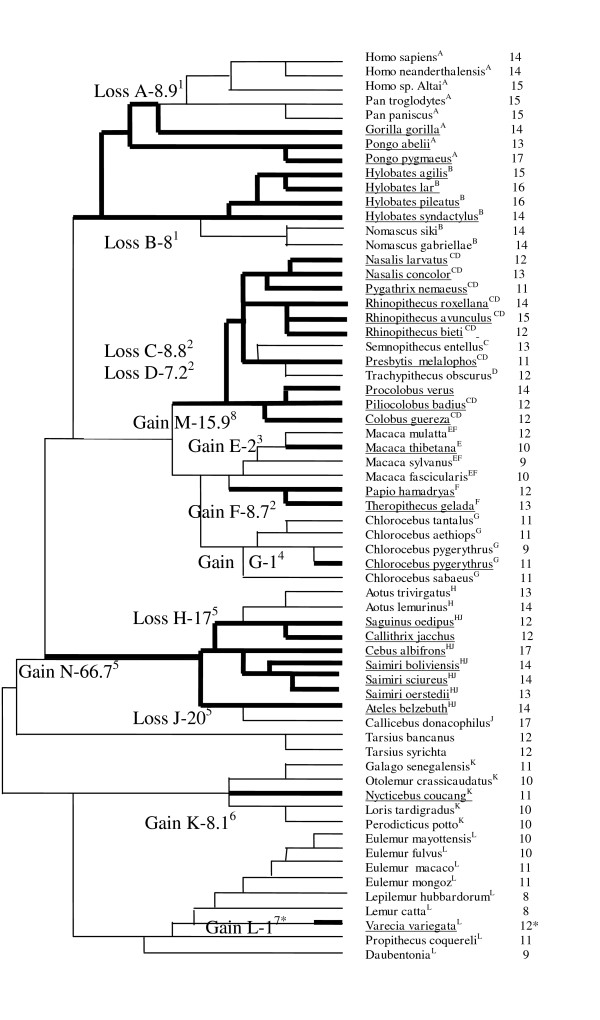
**Schematic representation of the phylogenetic relationship between the primate species examined for *gau *followed by numbers of stops in this region**. Underlined species possess a predicted mitochondrial antisense antitermination AGR tRNA, which potentially enables translation of the AGR codons without importing cytosolic tRNAs. Broad branches represent evolution in the presence of antitermination tRNAs, as predicted by parsimony. Branch lengths are not proportional to divergence times, but specific nodes used for contrast analyses between lineages with and without antitermination AGR tRNA are indicated by capital letters, together with estimated divergence times, because these lineages with and without antitermination AGR tRNA diverged. Divergence times (millions of years) shown are from the following references (superscripts match the corresponding nodes indicated by capital letters): [27]^1^, [28]^2^, [29]^3^, [30]^4^, [31]^5^, [32]^6^, [33]^7 ^and [34]^8^. *Analyses of several sequences indicate the possibility of a polymorphism in the presence of the antitermination AGR tRNA in *Varecia*. It is most parsimonious to assume that the presence of antitermination AGR tRNA is very recent in this genus.

Previous analyses [5] showed that the number of overlapping genes is greater in primate species possessing the predicted antisense antitermination tRNAs with anticodons matching the AGR codons than in sister taxa that are not predicted to possess such tRNAs. This coevolution between the translational properties necessary to translate overlapping genes and the overlapping genes themselves is significant evidence in favor of the hypothesis that overlapping genes are physiologically functional. A similar analysis is performed here testing whether the number of AGR codons in the predicted *gau *sequence of primates coevolves with the presence/absence of predicted tRNAs with anticodons that match the AGR codons. Figure 3 presents the phylogenetic relationships between 63 primate taxa, for which the complete mitochondrial genome is available in GenBank, the presence of predicted antisense antitermination AGR tRNAs in these species, and the number of stop codons in *gau*. Taxa were grouped to create monophyletic clusters of species predicted to possess this tRNA in view to compare to closely related monophyletic groups of species that were not predicted to possess a tRNA matching AGR codons. These pairs of monophyletic clusters were used to calculate the phylogenetically independent contrasts, A-N, which are shown in Figure 3. Groups are indicated by the letters A-N near taxon names, and the times of divergence that are shown are from various publications on primate phylogeny [27-34].

Phylogenetic contrasts for a given monophyletic group were calculated by subtracting the mean number of *gau *AGR codons in species that do not possess a tRNA matching the AGR codons from the mean in the phylogenetically matched taxa predicted to possess this tRNA. For example, group G indicates the gain, in one of the two available mt genomes of the velvet monkey *Chlorocebus pygerythrus*, of an antisense antitermination tRNA with an anticodon matching the AGR codons. The *gau *region in the *Chlorocebus pygerythrus *genome has 11 AGR codons, while the other available sequence from that species has only 9 AGR codons, and on average, *gau *sequences from *Chlorocebus *species that do not possess this tRNA have 10.5 AGR codons. Hence, the contrast for group G is +0.5 AGR codons in *gau*. Physiological activity expects a positive association between AGR codons and the presence of this tRNA. Thus, most contrasts are expected to be positive, as in the case of *Chlorocebus*. Indeed, 9 of the 13 contrasts (69%) that were calculated are positive, which is a statistically significant majority of the cases according to the robust non-parametric sign test (one tailed P = 0.0231). These contrasts are not equal in terms of estimation quality. Seven contrasts were calculated based on one single species in one of the groups being contrasted (as in contrast G). Three of the four negative contrasts belong to contrasts with low estimation quality due to a small sample size, which suggests that the actual strength of the coevolution between the presence/absence of mt tRNAs that recognize AGR codons and the number of AGR codons in *gau *is probably stronger. Further exploration of the data (not shown) suggests that the interaction between long divergence times between groups with and without this tRNA and a low sample size creates uncertainty in the contrast estimation. Indeed, phylogenetic reconstruction becomes more uncertain the further the reconstructed state/time is from the actual observed data, which in most cases are the actual species observed rather than the assumed ancestors (i.e., see the discussion in reference [35]).

**Figure 4:**
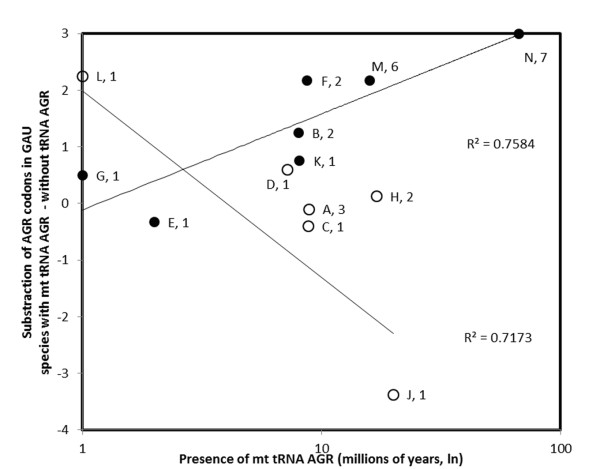
**Contrasts in the numbers of AGR codons in the mitochondrial *gau *genes of primates**. Analyses made between the lineages with and without antisense antitermination tRNA (from nodes A-N in Figure 3) as a function of the divergence times between these lineages. Numbers near data points refer to the smallest sample size used in the contrast calculation. Open symbols are for nodes where the antisense antitermination tRNA was presumably lost. Filled symbols indicate gains according to the parsimonious interpretations of Figure 3.

The various contrasts are also not equivalent in a different sense; parsimonious phylogenetic interpretations of the presence/absence of the tRNAs in Figure 3 suggest that some contrasts reflect a gain in tRNAs that recognize AGR codons (contrasts B, E, F, G, K, M, N), while an evolutionary scenario of loss fits the other contrasts (A, C, D, H, J, L). For gains, 6 of the 7 contrasts are positive, which is statistically significant according to a one-sided sign test (P = 0.03125). Half of the six contrasts assumed to be evolutionary losses are negative, which does not indicate any pattern. Previous analyses [5] also suggested that the time since a change in the presence/absence in AGR tRNA occurred affected the number of overlapping genes, which suggests that with time, overlapping genes evolve when the ability to translate them exists. This correlation between the contrasts associated with gains and the time since the gain occurred (times are indicated in Figure 3) exists for primate mt *gau*, as shown in Figure 4 (non-parametric Spearman rank correlation rs = 0.93, one-sided P = 0.0113). The correlation was unexpectedly negative for losses (non-parametric Spearman rank correlation rs = -0.77, two-sided P = 0.0836) but weaker and statistically insignificant.

This section presents two independent analyses that suggest that mt antisense antitermination tRNAs with anticodons matching the AGR codons translate *gau*: significant majorities of phylogenetic independent contrasts are positive; and the contrasts increase with time as the evolutionary ability of mitochondria to translate AGR codons is gained. The coevolution between *gau *and the mitochondrial translational apparatus strongly supports the hypothesis of the mitochondrial expression of *gau*. In vertebrate mitochondria, the expression of *gau *would require a modification of the vertebrate mitochondrial genetic code, which would mean reassigning the stop AGR codons to code for arginine [5].

### Data concerning the selective pressure on the *gau *ORF and protein structure

A relatively high level of amino acid sequence similarity has been found between alpha-proteobacterial proteins and metazoan Gau proteins. For example, in *Wolbachia *bacteria and *Drosophila simulans *Gau, the amount of sequence containing identical and functionally similar amino acids is 47.5% and 57.4%, respectively (Table 2). These values are very significant: higher values for identical amino acids are only found in some parts of the Cox1 proteins but not in other translated *cox1 *sequences from other potential reading frames (Table 2). The *gau *ORF is always found in the same reading frame, which is in the antisense strand overlapping the *cox1 *gene in such a way that the third position of the *cox1 *gene codons are complementary to the third position of the *gau *ORF triplets (Figure 1). This implies that in the first two positions, point mutations in the *cox1 *gene are almost always associated with major structural changes in the Gau protein. Therefore, changes in the *gau *and *cox1 *codons are limited.

**Table 2:** Comparison of the sequence alignment between the *Drosophila simulans *and *Wolbachia cox1 *genes in the six possible reading frames

Nucleotidic position on the *Drosophila cox1 *gene	1-381	382-684	685-988	989-1290	1291-1536
Number of nucleotides by region	381	303	303	303	246
% of identical nucleotide in the alignment	60.9	66.7	62.7	57.4	50.8
Gap/indel	0	0	3	0	0
+1 reading frame (*cox1*)					
a.a. position on the *Drosophila *Cox1 protein	1-127	**128-228**	229-329	330-430	431-512
Number of a.a. by region	127	**101**	101	101	82
% of identical a.a.	60.6	**68.3**	56.4	46.5	36.6
% of identical and functionally similar a.a.	81.9	**91.1**	94.0	73.3	61.0
Gap/indel	0	**0**	1	0	0

+ 2 reading frame					
% of identical a.a.	23.6	**27.7**	34.6	27.7	16.0
% of identical and functionally similar a.a.	36.2	**38.6**	48.5	41.0	38.3
Gap/indel	0	**0**	5	3	0
% of stop codon in *Wolbachia *sequence	11.0	**5.9**	7.9	5.9	3.7
% of stop codon in *Drosophila *sequence	15.0	**8.9**	9.9	14.8	7.4

+ 3 reading frame					
% of identical a.a.	25.4	**22.7**	28.7	17.8	18.3
% of identical and functionally similar a.a.	46.8	**38.6**	44.6	38.6	43.9
Gap/indel	0	**0**	1	0	1
% of stop codon in *Wolbachia*	7.1	**4.9**	3.9	7.9	6.0
% of stop codon in Drosophila	2.4	**1.0**	2.0	3.0	1.2

-1 reading frame					
% of identical a.a.	26.2	**24.7**	30.7	21.8	19.3
% of identical and functionally similar a.a.	51.2	**48.5**	49.5	49.5	37.8
Gap/indel	1	**0**	1	0	0
% of stop codon in *Wolbachia *sequence	8.7	**6.9**	3.0	6.9	4.9
% of stop codon in *Drosophila *sequence	11.1	**13.9**	9.9	12.9	11.0

-2 reading frame (= *gau *ORF)					
% of identical a.a.	42.8	**47.5**	41.6	34.6	28.0
% of identical and functionally similar a.a.	58.7	**57.4**	48.5	39.6	48.8
Gap/indel	0	**0**	1	0	0
% of stop codon in *Wolbachia *sequence	3.2	**1.0**	7.9	8.9	3.7
% of stop codon in *Drosophila *sequence	1.6	**1.0**	3.0	4.0	3.7

-3 reading frame					
% of identical a.a.	23.1	**26.7**	25.7	19.8	17.3
% of identical and functionally similar a.a.	38.6	**35.6**	43.6	39.6	33.3
Gap/indel	0	**10**	1	0	1
% of stop codon in *Wolbachia *sequence	10.2	**10.9**	10.9	8.9	4.9
% of stop codon in *Drosophila *sequence	6.3	**9.9**	10.9	10.9	7.4

The sequences have been divided into five parts, including the *gau *region. Concerning the last region (values are in bold), the translations correspond either to the exact nucleotide sequence or to +/- 1 or two nucleotide(s) sequences according to the reading frame. For *Drosophila *and *Wolbachia*, the accession numbers are AF200838.1/AAF77368 and AE017196/AAS14036.1, respectively. The genetic codes are those of invertebrate mitochondria and of bacteria. The sequence of *Drosophila simulans *is preferred to that of *D. melanogaster *because the start codon is undetermined in the latter. Abbreviations (a.a.: amino acids).

Complementary analyses have been made to determine if the relatively high degree of conservation of the deduced protein sequence is under heavy selective pressure or is due to the sequence conservation of the *cox1 *genes. Analyses reveal that for 12/100 positions in the Gau protein, the amino acid changes preferably occur within the same family (F/L/I/M/V, K/N and Q/H). However, because the most conserved positions (first and second) on the *cox1 *codons correspond to the second and first positions on *gau *codons, respectively, changes within a functionally similar amino acid family are facilitated (for example: I/M, F/L, Q/H and N/K, but not I/F) (Figure 2 and Table 3).

**Table 3:** Functionally similar amino acid changes in Gau sequences

a.a. position on the Gau protein	11	16	22	24	46	44
	I (10)	F (8)	I (11)	I (10)	N (9)	Q(7)
	M (3)	L (5)	M (2)	M (3)	K (4)	H(6)
a.a. position on the Gau protein	54	59	60	65	67	73
	I (10)	I (7)	I (8)	I (9)	L (10)	N (9)
	M (3)	M (6)	F (5)	M (4)	F (3)	K (4)

This analysis was performed on the sequences in figure 2, excluding the human mitochondrion. Only positions for which all amino acids are functionally similar are mentioned. The number of sequences containing a given amino acid is in parentheses.

Moreover, overlap coding for *gau *by the mitochondrial *cox1 *gene implies that synonymous codon usage is optimized to enable overlap coding on the opposite strand. Simulations of the human mitochondrial *cox1 *region which randomly reassign synonymous codons according to synonymous codon frequencies found in the human *cox1 *gene enabled us to test this hypothesis. These simulated sequences do not alter the sequence of the protein as coded by the main frame but do alter the sequence of Gau. The third codon position of Gau, which corresponds to the third codon position in *cox1*, alters synonymous codons in the two-fold codon families in *cox1 *but does not alter the amino acids putatively encoded by Gau. Therefore, simulations can only alter *gau *at *cox1 *codons from four-fold codon families corresponding to codons in *gau *to an amino acid encoded by a two-fold codon family. Greater possibilities exist for the six-fold codon families coding for leucine and serine. However, this suggests that the simulations that randomly reassign synonymous codons at the *cox1 *gene level have limited freedom to alter Gau. In addition, synonymous codon usages are biased, which decreases the possibility of altering Gau even more. If, for example, nine out of ten codons that code for phenylalanine are UUU and only one is UUC, the simulations will alter the original codon at most in only 2 cases.

On average, the simulations altered "only" synonymous codons in 51.4 percent of *cox1 *but altered 53.5 percent in the region corresponding to *gau*. This result is statistically significant. Twenty independent simulation replications were performed and a paired t test between the percentages altered in the *cox1 *gene at large versus the *gau *region indicated that this difference is statistically significant (two-tailed paired t test, P = 0.00479). This suggests that the amino acid composition of Gau is slightly more open to alterations by simulations than the rest of *cox1*. This result, if interpreted as the result of selection, suggests an adaptation allow some freedom for *gau *to evolve independently of *cox1 *and more than for other regions of *cox1*, which are presumably not involved in overlap coding.

Altering synonymous codons at the *cox1 *level does not necessarily alter the amino acids encoded in Gau, as explained above. Indeed, on average, 79.8 percent of Gau amino acids remained unchanged by simulations, which means that on average only 37.8 percent of the altered codons resulted in amino acid changes in Gau. Because of these relatively high constraints on the effects of simulations on coding properties of *gau*, simulations should not have altered much GAU. So, we blasted [36,37] the amino acid sequence coded by each of the 20 simulated *cox1 *sequences. These aligned with the same sequence as found for the original GAU sequence translated for that frame from the natural *cox1 *sequence, as found previously [5]. However, the non-random nature of the natural *cox1 *coding sequence, in relation to the overlapping *gau *coding region, is revealed by the observation that the alignment quality is systematically lower for simulated sequences than for the natural sequence: 81.2 percent of the residues were identical or similar for the natural sequence, and on average, only 77.8 percent (standard deviation 1.6) were identical or similar for the 20 simulated sequences (two-tailed, one sample t test, P < 0.0001). This means that Gau proteins translated from simulated human *cox1 *sequences differ more from the GenBank sequences than the natural sequence. Hence, the natural sequence is optimized toward a given coding content and its high quality alignment with the GenBank sequence is not due to chance. If no such optimization existed, simulated sequences when compared to the GenBank sequences would have had greater similarity to the GenBank sequences than the natural sequences in approximately half of the cases; however, this was not the case for the 20 simulated replications. It is important to stress here that this non-random result is even more unlikely considering that simulations alter so few Gau amino acids (approximately 80 percent remain unchanged). These simulation analyses suggest that c*ox1 *coding sequences are optimized to enable overlap coding for Gau and that matching an existing GenBank sequence of Gau is not the result of chance.

The ratio of non-synonymous (d*N*) to synonymous (d*S*) changes between taxa is frequently computed to determine the strength and direction of selection. Thus, protein and DNA sequences coding for Gau and a part of Cox1 (i.e., corresponding to the complementary regions of *gau*) from vertebrates and protostomia were retrieved from GenBank. Protein sequences were aligned using MUSCLE [38]. The correspondence between the protein alignments and each DNA sequence was established using Wise2 software [39]. The CodeML program of the PAML (Phylogenetic Analysis by Maximum Likelihood) software package was used to test for positive selection using the M2a and M1a models, respectively [40]. Likelihood ratio tests were calculated by comparing M1a to M2a with df = 2. The site model M2a was applied to test the evolutionary shift by maximum likelihood analysis for *gau*. This model allows the ω (= d*N*/d*S *ratio) to vary among sites. The ratios ω > 0, ω = 0, and ω < 0 signify positive selection (adaptive molecular evolution), neutral mutations, and negative selection (purifying selection), respectively. In our analyses, no significant positive selection was found, and a majority of the sites evolve with a ω (d*N*/d*S*) < 0 (data not shown).

### Distribution of nonsense mutations along *gau *ORFs

We have searched for nonsense codons that disrupt the coding frame of the *gau *gene in several different organisms. We downloaded available mitochondrial genomes from the NCBI Organelle Genome Resource [41]. A total of 1,525 complete genomes were analyzed. Among them, 1147 sequences from metazoa, 33 from fungi, 25 from plants and 28 from other eukaryotes were found to code for GSPPP (see below). This pentanucleotide was chosen because it is the most conserved domain among the *gau*-bearing taxa, and it has been found in more than 93%, 96%, and 97% of the fungi, alpha-proteobacteria and plant, and protostomian, and deuterostomian complete mitochondrial genomes, respectively. Moreover, most of the sequences that do not contain this region may be uncertain or have undetermined nucleotides (data not shown). As a cautionary note, it has been observed that approximately a quarter of the published complete mtDNAs contain misannotations, although they generally concern tRNA genes [3,42]. Using the highly conserved sequence GSPPP (Figure 2) as an anchoring position, we have searched for stop codons in: *i*) 100 codons upstream of the glycine of the GSPPP sequence; *ii*) 100 codons downstream from the glycine of the GSPPP sequence; and *iii*) 100 codons downstream of the start codon number 101 from the previous measure (Table 4). As shown in Table 4, the largest numbers of sequences from the 100 possible codons without nonsense mutations (223) are found 100 codons downstream of the GSPPP sequence (region *ii*) in contrast to the regions upstream (4) and downstream (90) of the *gau *sequence (regions *i *and *iii*, respectively). Sequences without nonsense stop codons are found mainly in protostomia.

**Table 4:** The taxonomic distribution of nonsense codons in the GAU sequence among mitochondrial genomes

		a) 100 aminoacids upstream GSPP sequence												b) 100 aminoacids downstream GSPPP (GAU sequence)												c) 100 aminoacids downstream GAU sequence											
		Number of sequences with STOP codons										NoSC*		Number of sequences with STOP codons										NoSC*		Number of sequences with STOP codons										NoSC*	
		Position of last STOP codon starting from 5'												Position of 1rst STOP codon starting from 5'												Position of 1rst STOP codon starting from 5'											
	Phylum	10	20	30	40	50	60	70	80	90	100			10	20	30	40	50	60	70	80	90	100			10	20	30	40	50	60	70	80	90	100		
Metazoa/Protostomia	Arthropoda			1			18	4		57	22	1		4					3			4	5	87		2				1			86	2	1	11	
	Annelida							1		2	2													5										1		4	
	Brachiopoda						1			2														3									1			2	
	Bryozoa						1																	1									1				
	Chaetognatha						1			1														2									1			1	
	Entoprocta							1		1									1					1									2				
	Echiura						1																	1									1				
	Mollusca			3			9	8		13	4			1		1		3		2		3		27		1	2	3					12	1		18	
	Priapulida						1																	1												1	
	Onychophora									2									1					1												2	
																																				
Metazoa/Deuterostomia	Chordata							11		76	798			1	751	92	36	3		1			1			4	824	20		12		1	16			8	
	Echinodermata							6		9	1													16									8			8	
	Hemichordata							1		1														2									2				
	Xenoturbellida							1																1									1				
																																				
Metazoa/Pseudocoelomata	Nematoda						3			4		1		6					1					1					4				4				
																																				
Metazoa/Acoelomata	Platyhelminthes						11	1		14				1				18			1	2	3	1		7				12		1	6				
																																				
Metazoa/Cnidaria	Cnidaria						2			26													6	22									27			1	
																																				
Metazoa (other)	Placozoa						1	2																3												3	
	Porifera						2	1		17		1						1						20						1			1			19	
																																				
Fungi/Dikarya	Ascomycota						3	1		14	2	1		4					1					16		1		1	2	10						7	
Fungi/Dikarya	Basidiomycota			1			1				2			4										1						5							
Fungi	Blastocladiomycota									1	1			1																1							
Fungi	Chytridiomycota									3	3			4					1				1										6				
																																				
Viridiplantae	Chlorophyta									3	3			1									3	2					1				5				
	Streptophyta									15	4										17		2									18	1				
																																				
Protists/stramenopiles	Bacillariophyta									1														1												1	
	Phaeophyceae									2	3										5											2	3				
																																				
Eukaryota (other)	Eukaryota (other)						2	1		7	12			8					1				5	8						2	1	4	11			4	
																																				
	Total without STOP codons												4												223												90
	Total with STOP codons												1229												1010												1143

The table is divided into three main sections: a) 100 codons upstream of the GSPPP motif in the GAU sequence; b) 100 codons downstream, starting in the GSPPP motif; and c) 100 codons starting at codon 101 from the GSPPP motif. In each section (a, b and c), the 100 codons are divided into 10 groups of 10 codons each, and the table cells indicate the number of stop codons found in each division. For section (a), the cells indicate the position of the last stop codon starting from the 5' end and for sections (b) and (c) the position of the first stop codon starting from the 5' end. The cells in gray highlight sequences with a substantial portion without nonsense codons. NoSC* indicates the number of sequences without nonsense codons.

**Figure 5:**
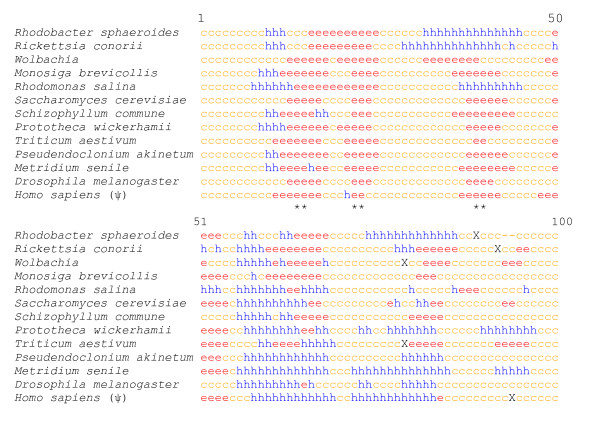
**Prediction of the secondary structure**. The HNN (Hierarchical Neural Network) prediction method was used (http://npsa-pbil.ibcp.fr/cgi-bin/npsa_automat.pl?page=/NPSA/npsa_hnn.html). (h: α helices, e: β strands, c: random coil, X: stop codon; *: conserved secondary structure).

**Figure 6:**
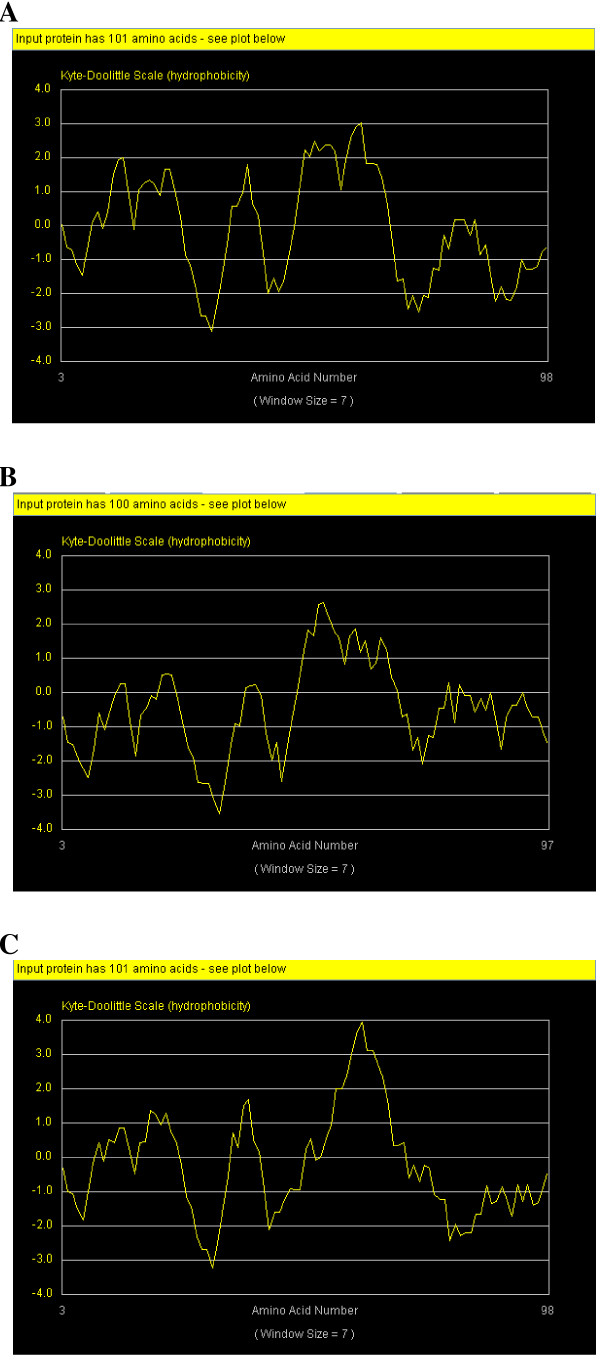
**Hydropathy plots for Gau proteins**. This figure shows the hydrophobic domain comprising approximately two-thirds of the whole protein. A, B and C are hydropathy plots of *Monosiga brevicollis *(sequence deduced from the reverse strand of the *cox1 *gene), *Saccharomyces cerevisiae *(sequence deduced from the reverse strand of mt-*cox1 *gene) and *Macaca mulatta *(sequence deduced from a part of chromosome 1), respectively. Images are from http://arbl.cvmbs.colostate.edu/molkit/hydropathy/ (Kyte-Doolittle).

To expand our knowledge regarding Gau proteins, different types of analyses using structural bioinformatic tools were performed. Sequence similarity is not uniform throughout the Gau proteins, but a computer-aided prediction of the secondary structure of eukaryotic Gau proteins showed β strands in the NH_2 _part of the protein and α helices (with the exception of those of *Monosiga brevicollis *(protist)) in the first part of the COOH-region (Figure 5). Moreover, the Gau proteins (with the exception of those of alpha-proteobacteria) contain two regions (near their middle): a positively charged domain followed by a very hydrophobic one. In addition, examination of the hydrophobicity profiles revealed a convincing relationship between the Gau proteins; all exhibit similar profiles, which suggest a close structural equivalence (Figure 6). Various programs have been used to predict the transmembrane regions and/or signal peptide: Phobius [43], PrediSi [44], PSORT [45] and PRED-TMR [46]. However, no transmembrane domain or signal peptide has been highlighted, which suggests that these proteins could be localized in the periplasm. It should be mentioned that these proteins do not have any obvious motifs or domains that are conserved in other proteins in databases, such as the PROSITE database.

### Transcription of the *gau *region

BLAST analysis was performed to determine EST EMBL/GenBank database sequences similar to the *gau *regions of various taxa. However, the main problem encountered during this type of search is that many of the ESTs are known to be misoriented [47], and the EST sequences corresponding to the *gau *region could be doubtful. Nevertheless, we combined multiple results to infer the correct orientation of the mRNAs and ESTs, including sequence type (mRNA or EST), CDS annotation, data on the cloning strategy and poly(A) signal/tail. The presence of the poly(A) tail is an unambiguous criterion to determine the EST orientation; however, the nature of the 3' end post-transcriptional modifications in mitochondria varies significantly among organisms. Poly(A) tails have been detected in most plant mitochondrial mRNAs, in some trypanosomal (protist) and mammalian mitochondrial mRNAs [48], and in numerous mitochondrial ESTs of the chaetognath *Spadella cephaloptera *(unpublished data). In addition, the poly(A) tail is generally removed during cloning. Therefore, in databases, ESTs containing sequences similar to the whole *gau *regions and bearing a poly(A) tail are very rare. However, examples belonging to a plant, a mollusk and an insect were found (Additional file 2). No EST sequence containing both the entire *gau *region and a poly(A) tail was found in vertebrates; however, examples of two murid sequences cloned unidirectionally using an oligo dT are shown in Additional file 2.

### Immunolocalization of human Gau proteins

**Figure 7:**
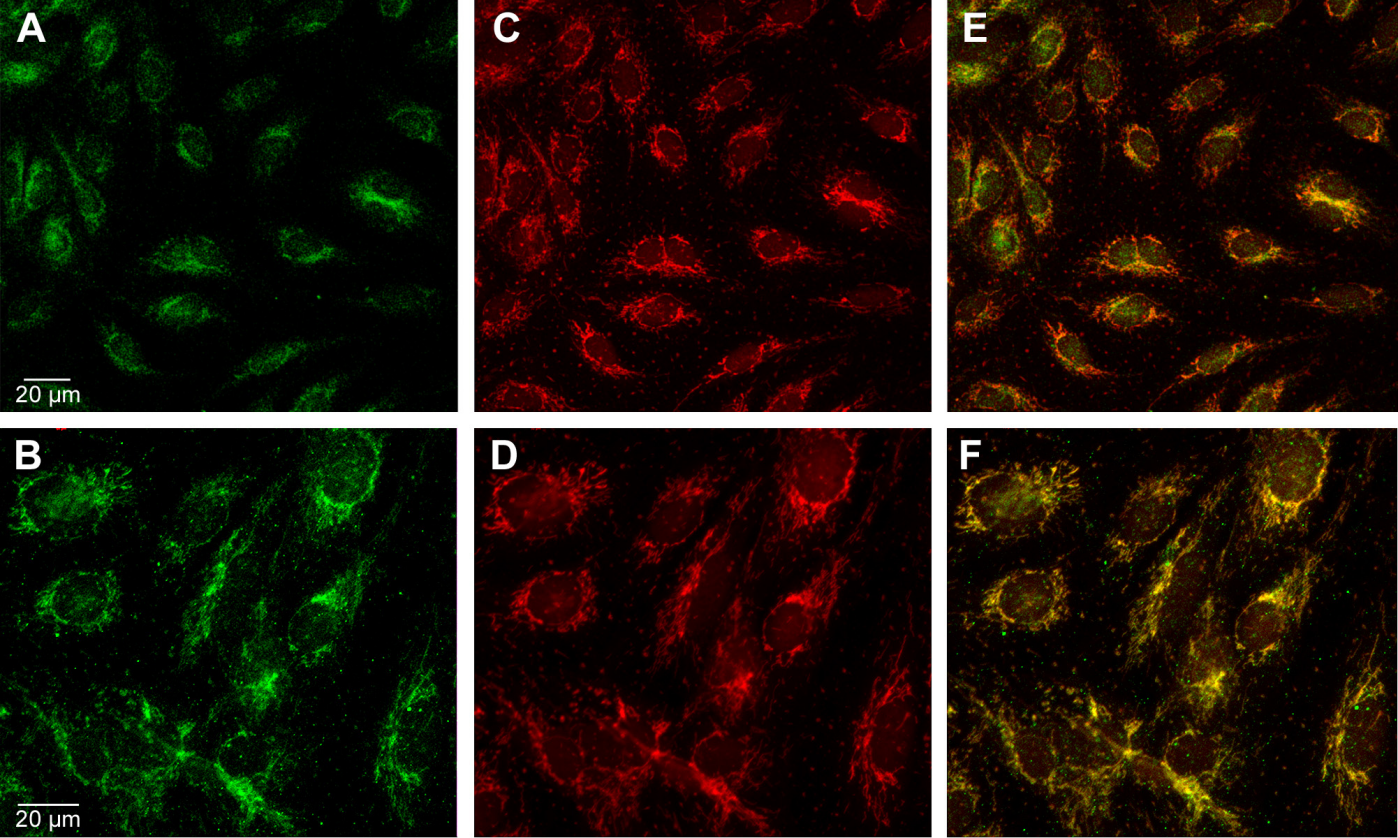
**Immunolocalization of Gau protein**. Immunolocalization of Gau protein (green) in HUVEC cells (A, B). In (C, D), localization of MitoTracker Red CMXRos, a mitochondrial marker (red). In (E, F), colocalization of Gau protein (green) and mitochondria (red). After reaching confluence, human umbilical vein endothelial cells (HUVEC) were incubated with MitoTracker Red CMXRos (Invitrogen, France). Mitotracker Red CMXRos (200 nM) was added to the cells for 10 min. After this incubation, the cells were washed for 10 min with HBSS and then fixed directly with 4% PFA for 3 min at 4°C. The cells were covered with Vectashield mounting medium to avoid bleaching the fluorescence. All steps were performed in the dark. For immunodetection of Gau proteins, the mouse monoclonal antibody developed by ProteoGenix (Oberhausbergen, France) was used. To minimize any non-specific antibody binding, the cells were first incubated in 10% normal goat serum in PBS (0.1 M) containing 0.1% Triton X-100 and 2% bovine serum albumin (PBS buffer) for 1 h at room temperature. Then, they were incubated overnight at 4°C in the primary monoclonal antibody against Gau, which was diluted at 1/100 in a PBS (0.1 M) solution containing 10% normal goat serum and 2% bovine serum albumin. After being rinsed three times, the cells were incubated for 1 h with the secondary antibody, Alexa 488-coupled anti-mouse IgG (Invitrogen) diluted at 1:400. Finally, the cells were rinsed in PBS and mounted in a medium containing an antifading agent (Gel/MountR, Bibmeda, USA). The primary antibody was omitted for the controls. Images were acquired using the Zeiss LSM 710 NLO confocal microscope (63x objective, numerical aperture 1.35). All parameters (laser percentage and voltage, light, gain, exposure, offset values) were adjusted to achieve the best results.

An anti-Gau monoclonal antibody was produced by Proteogenix (Oberhausbergen, France, http://www.proteogenix.fr) using the peptide sequence GSPPPAGSKKEVLK as the antigen. This peptide corresponds to a eukaryotic consensus sequence of the NH_2 _terminus of this protein (residues 2 to 15) (Figure 2). BLAST analysis revealed no significant alignments of this peptide with the GenBank human protein sequences (data not shown). A higher degree of similarity was found in one and six proteins with 10 and 8 identical amino acids out of 14, respectively. Moreover, identical amino acids are non-contiguous in the primary sequences, and the corresponding proteins are not localized to the mitochondria. Among the proteins having 7 identical amino acids in the peptide sequence is a protein with a mitochondrial localization signal, but the alignment contains a gap. Use of the anti-Gau antibody has revealed a strict co-localization of Gau proteins and the mitochondrial marker MitotrackerRed CMXRos in human umbilical vein endothelial cells (HUVEC), and almost all Gau proteins are localized to the mitochondria (Figure 7). Moreover, control experiments using preimmune serum remained negative.

## Discussion

The origin of novel genes is mostly attributed to processes, such as the mutation, duplication, and rearrangement of genetic segments. A rarely considered alternative is that proteins may arise continuously *de novo *(i.e., from a non-coding sequence). The possibility of generating new genes from pre-existing nucleotide sequences was first suggested by Grassé [49], who called it "overprinting". There are two principal types of overlap: (1) the same-strand type in which the two genes involved are transcribed from the same strand but in different reading frames and (2) the different-strand type in which the two genes are transcribed from different strands. Overlapping genes are known to be common in viruses, mitochondria, bacteria, plasmids and even in vertebrates (references in [50,51]). However, with the exception of viruses [52], it is widely assumed that only one strand can encode a protein and that the non-coding strands do not, although they can play a role in regulation [53]. The term *janology *(from the two-faced Roman deity Jano) has been proposed for the homologous relationship among the sequences sharing the same DNA [54]; although the term refers to *Jano*, it would be more appropriate to use this term for naming proteins encoded by the complementary strands of a DNA duplex. In the present study, analysis of complete mtDNAs has revealed an ORF in the complementary strand of the *cox1 *gene. Interestingly, the mitochondrial gene content is highly variable across extant eukaryotes. However, despite the disparity in size, all mtDNAs encode long and small subunit ribosomal RNAs and two components of the mitochondrial electron transport chain: Apocytochrome b and Cox-1 [4]. Evidence now firmly supports that mitochondria have a single origin; they arised from a eubacterial symbiont whose closest contemporary relatives are found within the alpha-proteobacteria [8,55]. The sequence of the deduced Gau proteins is relatively highly conserved not only in protist, plant, fungal and animal mtDNAs but also in alpha-proteobacteria. Its lack in other bacteria taxa strongly suggests an ancestral co-evolution of this region over the last 1.5 to 2.0 billion years [56].

Due to alternative translational mechanisms, it is difficult to predict the exact size of the *gau *coding region. Alternatives to the AUG start codons are very common among mtDNA genes, so it is unusual to find an AUG codon at the beginning of orthologous genes from different species ([13,57] and references there in [58]. For example, in almost all of the taxa, some of the NUN codons could be used as start codons. Interestingly, in the N-terminal region of the putative Gau proteins, a methionine or an isoleucine has generally been found at the same position, and it is known that the AUC/AUU/AUA codons (normally an isoleucine codon) can serve as initiation codons. In addition, in the transcript, an initiator AUG codon can be created by RNA editing [9]. The physiological translational stop codons are also unknown. However, codons in position 101, in reference to the putative initiation codon, are generally *ochre *(UAA) or *amber *(UAG) stop codons, which are the most efficient stop codons, strongly suggest a possible termination of the ORF at this position. Moreover, in the *gau *ORFs, the UAN codons are rare, and when present, the UAC or UAU triplets are preferentially translated into tyrosine. In addition, when internal stop codons are found in the *gau *sequences of both bacteria and plants, it is almost always the *opal *stop codon (UGA). However, it is not only the least efficient translational terminator, but there is evidence in eukaryotic and bacterial cells that it is translated by the tryptophan-tRNA [59,60]. The fact that these internal *opal *stop codons have been found at almost always the same position where the tryptophan amino acid is encoded in the other sequences (e.g., *Drosophila *and protists *Monosiga brevicollis *and *Rhodomonas salina*) suggests that these codons could be read. It is well known that in numerous taxa, mitochondrial codon usage varies from the standard genetic code, principally in the use of UGA as a tryptophan codon [13]. Sequence determination of metazoan mt protein genes followed by the comparison of the deduced amino acid sequences with those of other species has shown that the amino acid assignment of the codons AGA/AGG differs among metazoans [61,62]. Instead of arginine, the AGA/AGG codons specify serine in most invertebrates, including protostomia and echinoderms (references in [63]). Several arthropods translate the codon AGG as lysine instead of serine [64], and the protein genes of diptera (insects) do not contain the codon AGG [65]. In vertebrates, one or both of the AGA/AGG codons are generally absent or rarely used as stop codons [61]. Surprisingly, these two codons are numerous in vertebrate *gau *sequences (for an example, see the mitochondrial human sequence in Additional file 1). This is compatible with the cytosolic synthesis of Gau, perhaps from the nuclear mitochondrial pseudogene and the *in situ *mitochondrial expression of the *gau *gene, which may be induced by importing cytosolic AGR tRNAs with cognate Arg into the mitochondria. Mitochondrial synthesis of Gau independent of cytosol import is also possible due to the production of mitochondrial antisense, antitermination suppressor tRNAs with anticodons that match AGR stop codons [5] (see also Figures 3 and 4).

If we hypothesize that the size of the *gau *ORF is likely approximately 100 codons, various data showing the high degree of conservation of the deduced Gau protein sequences indicate that the *gau *sequence is under relatively heavy selective pressure and that it is not due to the overprinting bias. This assumption is based on the following facts: 1) the conserved positions of both the potential initiation and stop codons, 2) the high level of synonymous mutations, and 3) the *cox1 *genes are organized in exons and introns, as shown in some taxa [66-68] and in these taxa, the *gau *ORFs are complementary to only one part of an exon (see the examples in Table 5). Interestingly, in some species, the last nucleotide of the *gau *stop codon corresponds to the first nucleotide of the *cox1 *exon. This is another element supporting the functionality of the *gau *ORF products.

**Table 5:** Position of the *gau *ORF in the *cox1 *exon

Organism	Phylogenetic position	Accession number of the *cox1 *gene	Position of the *cox1 *exon containing the *gau *ORF/total number of exons	Nucleotide position of the *cox1 *exon containing the *gau *ORF	Position of the *gau *ORF in this exon
*Monosiga brevicollis*	Choanoflagellida (unicellular and colonial flagellate eukaryotes)	NC_004309.1	2/4	**2390**-2723	**2390**-2692

*Rhodomonas salina*	Cryptophyta (algae)	NC_002572.1	2/3	4304-4835	4485-4787

*Saccharomyces cerevisiae*	Fungi; Dikarya	NC_001224.1	4/8	6691-7167	6834-7136

*Prototheca wickerhamii*	Viridiplantae; Chlorophyta; Trebouxiophyceae (green algae)	NC_001613.1	2/4	c.3218-**3540**	c.3238-**3540**

*Pseudendoclonium akinetum*	Viridiplantae; Chlorophyta; Ulvophyceae (green algae)	NC_005926.1	2/5	c.5858-**6180**	c.5878-**6180**

*Metridium senile*	Metazoa; Cnidaria	NC_000933.1	1/2	1-893	393-695

The nucleotide position is printed in bold letters where the last nucleotide of the *gau *stop codon corresponds to the first nucleotide of the *cox1 *exon. Abbreviations: c., complementary strand.

Despite the failure to identify the physiological translation initiation and stop codons, we hypothesized that the *gau *ORFs could encode at least a 100 amino acid sequence. Because, for example, in the invertebrate mitochondrial genetic code, two of the sixty-four codons encode stops, an ORF that contains at least 100 codons is unlikely to appear by chance in the non-coding sequence with an average base composition. Thus, the presence of an ORF ≥100 codons is frequently considered to be a rough indication that the sequence is, or is not, protein-coding. Frequently, only ORFs greater than 100 codons in length are annotated as genes, and as a consequence, any ORF ≤100 codons is treated as spurious until proven otherwise through either experimental or comparative work. The *gau *ORF is longer than the minimal size compatible with a functional protein. For example, in metazoans, ATP8 is the smallest mitochondrially encoded protein; it is only approximately 50-65 amino acids long, and only half a dozen of these amino acids are well conserved across animal mtDNAs [69]. Approximately, twenty identical amino acids have been found in the deduced Gau protein sequences of both eukaryotes and bacteria. Moreover, due to overprinting, the composition of the Gau proteins is biased, and the level of amino-acid conservation is low for a mitochondrial protein. Interestingly, the mitochondrial protein ATP8 is not only well conserved within the metazoans and alignments could contain indels [56], which is contrary to most of the Gau alignments. Interestingly, ATP8 is more conserved at the level of secondary structure and in the chemical characteristics of the amino acids than in specific amino acid identity [55]. Similarly, comparisons of the secondary structure, especially the hydrophobicity profiles of Gau proteins, suggest that these putative proteins are structurally equivalent and could have similar physiological roles (Figures 5 and 6). Some amino acid differences have been found between eukaryote and bacterial proteins. However, this could be due to different constraints between the mitochondrial periplasm and the bacterial cytoplasm. In addition, these proteins do not have any obvious motifs or domains that are conserved in other proteins in the databases. Two reasons could explain this result: first, the Gau protein may be unique, and second, *gau *is an overprinting gene. Indeed, the creation of new coding sequences by overprinting has many constraints [70]. Mutations must allow an optimal adaptation of the new gene without inducing unfavorable mutations in the original gene. The maintenance of two functional overlapping genes is likely to constrain the ability of both genes to become optimally adapted. Genes that have arisen by overprinting can be identified by their biased composition. Thus, the new gene will exhibit unusual codon usage and encode new proteins with physicochemically biased properties [71]. The *gau *gene has a strongly biased composition, and both the first and second codon positions are "locked" (Figure 1). Interestingly, a recent study suggested that some viral overlapping reading frames that encode hypothetical proteins with highly unusual and biased composition, often discarded as non-coding, may in fact encode proteins [70].

Integration of mt genes into the nuclear genome is a physiologically important process contributing to the origin and evolution of the eukaryotic cell [63]. The transfer of entire genes from the mitochondria to the nucleus appears to continually occur in some plants (references in [72]). Although the transfer of entire genes seems to have ended in animals, DNA fragments of mitochondrial origin continue to integrate into the nuclear genome (references in [73,74]). Copies of mitochondrial sequences that are present in nuclear genomes are commonly referred to as "numts" (nuclear mitochondrial-like sequences) [75,76]. Although numts are common in several taxa, the stable nuclear absorption of mtDNA sequences does not appear to be universal. No numts have been reported in *Plasmodium falciparum *(protist)*, Caenorhabditis elegans *(nematode), or *Drosophila *(insect), although these organisms are well studied [76]. Moreover, functional mt genes transferred to the nucleus via endosymbiotic gene transfer no longer occurs in animals but is still an active process in green algae and land plants [77-79]. Numerous *gau*-like nuclear sequences have been found in vertebrate nuclear genomes, principally in *cox-1 *pseudogenes, but the analysis of the flanking regions suggests a strong selection pressure favoring sequence conservation only in the *gau *regions. Moreover, the proteins deduced from these regions exhibit a surprising homology level with the mitochondrial paralogs, which suggests possible nuclear transcription. This allows genes to evolve independently of the *cox1 *genes and possible lecture of vertebrate mitochondrial stop codons AGA/AGG [56], although mechanisms enabling *in situ *mitochondrial expression exist either via importing cytosolic tRNAs or independent of import by mitochondrial antisense antitermination tRNAs [5] (see also Figures 3 and 4).

Estimations of the ratio of synonymous and non-synonymous substitutions (d*N*/d*S*) at sites in the overlapping reading frames of the *gau *and *cox1 *regions (complementary to *gau) *provide strong evidence for an evolutionary mode under purifying selection. The selective pressure is similar for these regions in both protostomia and vertebrates, which suggests that also in the latter taxon, Gau proteins are also mitochondrially encoded. Interestingly, in viruses, it has been shown that d*N*/d*S *rates were consistently lower in regions with more overlap than in those with less overlap; however, these analyses only concern genes which overlap on the same strand ([80] and references there in). Other analyses, in which genes overlapping on the opposite strand have been studied, show that false results concerning the nature of the selection are not unfrequent and that non-functional overlapping ORFs are evolutionnary conserved because their sequence is shared with functional genes [81,82]. This strongly suggests that bioinformatic analyses of evolutionary conservation could be uninformative in the case of overlapping genes.

Because of the problematic assignment of the orientation of ESTs, unambiguous sequences corresponding to the *gau *region are rare in the database. However, some EST sequences bearing the entire *gau *region containing a terminal poly(A) tail exist. It is probable that the EST sequences for which the orientation is unknown could correspond to the transcriptional regions of *gau *sequences. It is also possible that, as has been already shown for another janolog sequence (*ribin*, which is found on the complementary strand of the *28S *rRNA gene [83,84]), the relatively low level of *gau*-EST sequences may result from transcription that is tissue specific, by transcription that only occurs in infected/stressed organisms or during particular stages of development. In addition, it must keep in mind that both same-strand and opposite-strand overlapping ORFs may be transcribed regardless of functionality [85]. Moreover, immunohistological experiments using an anti-Gau monoclonal antibody clearly showed a mitochondrial specific signal in human cells. The human mitochondrial proteome consists of an estimated 1,100-1,400 distinct proteins, only 13 of which are encoded by the mtDNA. In addition, approximately 15% of the mitochondrial proteome awaits identification [86], and the Gau proteins could be one of these still unknown proteins.

## Testing the hypothesis

The presence of the Gau proteins still remains hypothetical. Concerning the anti-peptide antibody, based on the expected N-terminal sequence of Gau, experimental studies are needed to determine its size, whether the antibody reacts with a single protein, and whether the sequence of the immunoreactive protein corresponds to the predicted *gau *gene product. Moreover, a set of immunohistological experiments is required to demonstrate that these proteins are present and functional both in eukaryote and alpha-proteobacteria cells. This includes the use of monoclonal antibodies against the putative amino, central, and carboxyl terminal synthetic peptides of GAU proteins, Western blot analysis and immunological analyses of different subcellular enrichments from several cell types. These immunoprecipitation analyses are required to determine if the *gau *ORF could encode a functional protein in the mitochondria. Mass spectrometry could be applied to identify the Gau proteins from highly enriched mitochondrial extracts. This hypothesis could also be tested by purifying the product of the *gau *gene and determining its sequence. Cell biological experiments are needed to determine the physiological role of this protein. However, in preliminary studies, this hypothesis can also be tested genetically by constructing yeast or alpha-proteobacteria strains with a tagged *gau *gene and analyzing *gau*-negative mutants. The tag would allow the purification of Gau by affinity chromatography and analysis of the purified protein by mass spectrometry. Yeast is a useful organism that can be used to gain a better understanding of the genetic and molecular properties of mitochondria. Moreover, the general conservation of both mitochondrial genes and pathways between human and yeast allows us to use yeast as a model for some diseases [87]. Similarly, the alpha-proteobacterium *Rhodobacter sphaeroides *is a popular model system already used to demonstrate the functional effects of human mitochondrial DNA mutations, including those within the structural subunits of cytochrome *c *oxidases [88,89]. However, it must not be forgotten that in yeast and alpha-proteobacteria, the *gau *region is on the opposite strand of the *cox1 *gene. In vertebrates, *gau *regions are in the nucleus as a possible functional pseudogene and on the complementary strand of the mitochondrion-encoded *cox1 *gene. Moreover, it also stresses that future studies are likely to confirm the existence of the other putative overlapping genes [5].

## Implications of the hypothesis

The protein families involved in basic cellular pathways are highly conserved throughout evolution, and despite the fact that further work is required to determine whether the *gau *ORF encodes a functional protein, we hypothesize that studies of this ORF will highlight the evolutionary origin of mitochondria and the possible overlapping mitochondrial genetic code. Moreover, despite the small size of the mitochondrial genome, mtDNA mutations are important causes of inherited disease. Indeed, pathogenic point mutations and rearrangements of the mtDNA have been reported in a wide spectrum of clinical disorders, including neurodegenerative disease, aging and cancer (reviewed in [1,86]). However, many challenges remain. Relatively little is known concerning the precise pathophysiological mechanisms that lead to cellular dysfunction and pathology, and we hypothesize that studies of Gau proteins could help to provide a better understanding of mitochondrial disease.

## Declaration of competing interests

The authors declare that they have no competing interests.

## Authors' contributions

EF conceived the study and drafted the manuscript. LD analyzed the complete mtDNA. ST conducted the cell culture experiments. RB performed the immunolocalization experiments. HS and AL performed the bioinformatic analyses and wrote the corresponding analyses. AL, LD, ST, RB and HS have critically revised the manuscript.

## Reviewers' comments

Changes are highlighted in yellow in the manuscript

### Referee 1: Arcady Mushegian, Kansas University Medical Center

The manuscript by Faure et al reports an intriguing observation of a novel conserved putative ORF in the antisense orientation of the mitochondrial and alphaproteobacterial *cox1 *genes. I am somewhat concerned about the significance of these observations, since most of the case is made by pointing out various aspects and consequences of high sequence conservation of this ORF, which itself is a consequence of its overlap with also conserved *cox1*.

Authors' response: Part of the supporting data relates to the simulations showing that natural sequences are optimized for overlapping coding with the cox1 region, which is also assumed to code for gau. In addition, variation within gau among primates coevolves with the part of the mt translational apparatus specifically required for gau's translation. Such analyses are not dependent on alignments and conservation and are themselves strong independent confirmation of the results based on conservation.

Western with anti-peptide monoclonal antibodies is good but not enough - have the authors tried to also detect the transcript using the strand-specific probe?

Authors' response: This experiment has not been performed; however, we performed BLAST searches of gau regions against the EST GenBank database. Although apparently rare, some correctly oriented EST sequences bearing the complete gau regions were found.

P. 5: "The deduced Gau protein sequence is relatively well conserved not only in protists, fungi, plants and animals mtDNAs but also in members of the alphaproteobacteria taxa (rickettsiales, rhizobacteria rhodobacterales, etc...) ... but not in other bacterial taxa" -- is the ORF for Gau found in the opposite strand of the *cox1 *in alphaproteobacteria? Do other taxa have *cox1*, and what to make of the lack of gau there?

*Authors' response: We have added this sentence in the MS: « In other bacterial taxa, such as proteobacteria other than alpha-proteobacteria, Chloroflexi, Cyanobacteria, Firmicutes and Actinobacteria, a region with a lower degree of similarity to the Gau protein can also be found after translation of the complementary strand of the genes encoding members of the cytochrome c oxidase subunit I-like SCOP superfamily *[9]. »

p. 6: "overlaps the *cox1 *gene in such a way that the third positions of gene codons overlap with the third positions of the overlapping ORF triplets" -- three "overlaps" in one sentence is a bit if stylistic trouble, but, more important, the meaning is unclear. One base cannot "overlap" another base, only be the same if they are in the same strand, or be complementary if they are in the opposite strands as is the case here.

Authors' response: As suggested by the referee, the changes have been made.

Ibid. "Therefore, *gau *codons have much restriction to change, as compared to those of *cox1*." -- not "as compared to", but rather "as well as"?

*Authors' response: Corrected*.

p. 6: Changes in 1st and 2nd position of either ORF will impact the other one strongly, no?

*Authors' response: This is now discussed in the manuscript: « ... However, because the most conserved positions (first and second) on the cox1 codons correspond to the second and first positions on gau codons, respectively, changes within a functionally similar amino acid family are facilitated (for example: I/M, F/L, Q/H and N/K, but not I/F) (Figure *2*and Table *3). *»*

p. 7 and following: GSPPP is not a motif, it is an exact match in all sequences - is it variable in some of the sequences that are not shown

Authors' response: Corrected. Data concerning the conservation of this sequence are now in the manuscript.

### Referee 2 - Prof Neil Smalheiser

Reviewer's report

**Title**: Probable presence of an ubiquitous cryptic mitochondrial gene on the antisense strand of the cytochrome oxydase I gene

**Version**: 1 **Date**: 27 April 2011

**Reviewer number**: 2

**Report form**:

This manuscript proposes the existence of a novel conserved protein, Gau, expressed on the antisense strand opposite the *cox-1 *gene. The evidence based on bioinformatics analyses is circumstantial and not compelling on its own, but does indicate that it is worthwhile testing the hypothesis. They went part of the way, by raising an anti-peptide antibody based on the expected N-terminal sequence of Gau, and demonstrating that human cells express immunoreactivity that is localized to mitochondria. However, they did not characterize the immunoreactive protein at all - does the antibody react with a single protein, and is it of the expected size? Does the sequence of the immunoreactive protein correspond to the predicted Gau gene product? I would want to see a Western blot (preferably of several cell types, and across different subcellular compartments within the cells), at least, before I would feel that their hypothesis has been validated experimentally.

*Authors' response: According to the « Instructions for **Biology Direct **authors » (http://www.biology-direct.com/authors/instructions/hypothesis), « hypothesis articles should present an untested original hypothesis backed up solely by a survey of previously published results rather than any new evidence. Hypothesis articles should not be reviews and should not contain new data. They should be articles outlining significant progress in thinking that would also be testable, though not so easily testable that readers will wonder why the testing has not already been done ».*

We agree with the referee's comments, and given the interest in these experimental studies, we have added a chapter entitled « Testing the hypothesis ». Moreover, in the revised manuscript, we consider that the convergence of different independent types of bioinformatic analyses is more than circumstantial and is a body of evidence worth consideration, although it is not yet compelling. Concerning the specificity of the antibody, as is now indicated in the manuscript, BLAST analyses suggest that no part of any known human proteins exhibits a high level of amino acid identity with the peptide antigen that is used for immunization and antibody production.

There are also widespread typos and errors in English usage that should be corrected.

Authors' response: Now after yours remark, the English of the MS has been corrected and improved by American Journal Experts.

### Referee 3 - Jeremy Selengut

Reviewer's report

**Title**: Probable presence of an ubiquitous cryptic mitochondrial gene on the antisense strand of the cytochrome oxydase I gene

**Version**: 1 **Date**: 3 June 2011

**Reviewer number**: 3

**Report form**:

**Major comments**:

Presentation of the hypothesis: Numbers are presented for the conservation between the *gau *ORF in *Wolbachia *and *Drosophila *and the statement is made that such levels are "very significant". It is not clear, however, setting aside for the moment the lack of stop codons, how much conservation would be expected given the corresponding conservation of the *cox1 *gene region on the opposite strands. A null hypothesis should be considered. What is the minimum amount of conservation that could be generated from the observed Cox1 protein sequences? Is there less conservation in the opposite strand in a different region of the *cox1 *coding sequence that has approximately the same relative conservation between *Wolbachia *and *Drosophila*? I find that the statement included following this, that the *gau *gene contains a positive patch and a hydrophobic patch, although not linked to any strong statement, is meant to imply that Gau is a "real" protein. I find this a very weak inference, as it is not clear to what extent random changes to the corresponding cox1 sequence might generate sequences in the opposite strand that have the "appearence" of protein-like structures.

Authors' response: The comparison between the sequences of Wolbachia and Drosophila has been improved. In the present analysis, the cox1 gene has been divided into five parts, including the region corresponding to gau sequence. This latter region corresponds to the most conserved sequence of the cox1 gene. Thus, a null hypothesis should not be excluded; especially because bioinformatic analyses (dN/dS) do not suggest a positive selective pressure on the mt gau regions. We agree with the referee's remark that we do not demonstrate that "Gau" proteins are producted. However, many arguments are provided that supports this hypothesis, including immunodetection and BLAST results from the EST database.

The case of *gau *regions in vertebrates: Special effort were put into finding *gau*-homologous regions in vertebrate genomes because of the presence of stop codons in the mitochondrial *cox1 *antisense sequences. These observations should be paired with control evaluations of genomes that have a mitochondrially-encoded *gau *region, and thus should be less likely to have nuclear versions.

*Authors' response: Analyses in the reference *[5]*by Seligmann, who is now a co-author of this MS, suggest that in vertebrates, two hypotheses are possible: mitochondrial recruitment of a nuclear pseudogene product or that Gau is the result of in situ mitochondrial expression using cytosolic tRNAs or mitochondrial antisense antitermination tRNAs. The second hypothesis (expression of a mitochondrial gene) is the most parsimonious hypothesis.*

Data concerning selection pressure of the gau ORF and protein structure: In the line "...mutations in the *cox1 *gene involve *de facto *important changes...", the word "important" should be changed or carefully defined. The word "important" is repeated at the end of that pararaph, but again without sufficient definition.

Authors' response: We have removed this ambiguous sentence. Moreover, the word "important" has been replaced by « major structural » changes.

The exposition of the analysis of possible changes is a good start, but devoid of any numerical or statistical treatment, it is impossible to determine the significance of the results. Are 12/100 more than expected to behave in the way shown? What are the odds of a random mutation constrained by the relative orientation of the Cox1 and Gau genes resulting in the retention of amino acids within their "conformational" selection groups?

*Authors' response: We agree with the referee's comment, especially because the results presented in Figure *2*show that the cox1 region that is complementary to the gau sequence is the most conserved. Thus, we added the following sentence: " ...because the most conserved positions (first and second) on the cox1 codons correspond to the second and first positions on gau codons, respectively, changes within a functionally similar amino acid family are facilitated."*

Discussion: The likelihood that a 100 amino acid open reading frame could arise by random chance in a non-coding stretch of DNA is alluded to, but is not really relevant to the issue of the Gau ORF. More germane is the likelihood that, given the observed conservation of the particular Cox1 region in question, stop codons could be avoided given all the possible allowed point mutations. For example, at some positions the observed Cox1 multiple sequence alignment might allow for so few possible amino acids that a stop codon in the corresponding Gau codon would be impossible. The fact that this ORF is aparrently conserved across large phylogenetic distances is very strong evidence, but it could be made much stronger by presenting a calculation showing that the number of possible ways of disrupting this ORF without harming the Cox1 gene is large and a very likely outcome absent selection for the Gau protein's translation.

*The data presented in Additional file *1*show that the number of stops within primate mt gau varies from 8 to 17. Hence, the coding constraints on cox1 do not prevent changes in the number of stops in gau. In addition, the fact that the number of stops coevolves with the ability to translate stops by suppressor tRNAs shows that the number of stops is not a random consequence of changes in coding at the cox1 level. In the human mt gau, there are 14 stops in the simulated sequences, which is constrained to keep cox1 unchanged. However, randomly mutated synonymous codons contain an average of 11.35 ± 1.5 stops, which are found at 21 locations throughout gau. Hence, at least 21 stops could exist within gau in humans without altering the protein encoded by cox1. The fact that the natural sequence contains more stops than the average simulated sequence may result from pressures to avoid gau's expression under conditions other than those in which suppressor tRNAs are present in the mitochondria.*

### Referee 3 - Jeremy Selengut

Reviewer's report

**Title**: Probable presence of an ubiquitous cryptic mitochondrial gene on the antisense strand of the cytochrome oxydase I gene

**Version**: 2 **Date**: 22 September 2011

**Reviewer number**: 3

**Report form**:

This work lays out an intriguing hypothesis that is well supported by the existing data and a number of preliminary calculations and experimental observations.

Whether the Gau transcript is a real and biologically important entity or a very persistent phantom remains to be experimentally tested. This manuscript provides a sound basis and impetus for that effort.

**Quality of written English**: Needs some language corrections before being published

Authors' response: Despite that we have already had the manuscript professionally copy-edited by American Journal Experts some parts which could be difficult to read have checked and improved if necessary for English language by one colleague.

## Supplementary Material

Additional file 1 **Alignments of mammalian Gau proteins and *cox1 *regions**. Characteristics of the nuclear *gau *sequences are shown in Table 1. For each species, the alignments of the nuclear Gau proteins have been provided with the sequences that have been deduced from the mitochondrial *cox1 *gene (mt.mt and mt.st are sequences that were translated using the standard and the mitochondrial (vertebrate) genetic codes, respectively). The alignments of the nuclear *cox1-like *regions are shown with those of the mitochondrial *cox1 *gene. For nuclear sequences, the chromosome number is shown after the two letters "Ch". The nuclear sequences corresponding to the *gau *regions are in bold. Characteristics of the nuclear sequences: *Homo sapiens, cox1 *(NC_001807, nt5905-nt7446), Ch1 (nt556317-nt557859), Ch14 (nt32023757-nt32022168); *Pan troglodytes, cox1 *(NC_001643, nt5321-nt6862), Ch2a (nt51808180-nt51806579), Ch8 (nt47844508-nt47845884); *Pongo pygmaeus, cox1 *(NC_001646, nt5331-nt6870), Ch2a (nt60553029-nt60554660); *Macaca mulatta, cox1 *(AY612638, nt5850-nt7391), Ch1 (nt108934590-nt108935852), Ch2 (nt123178799-nt123180392), Ch6(a) (nt30941431-nt30943006), Ch6(b) (nt50451345-nt50452956); *Equus caballus, cox1 *(EF597513, nt5359-nt6903), Ch27 (nt5205522-nt5203978); *Canis familiaris, cox1 *(U96639, nt5349-nt6893), Ch16 (nt9458239-nt9456847); *Bos taurus, cox1 *(NC_006853, nt5687-nt7231), Ch10 (nt4583738-nt4585281); *Mus musculus, cox1 *(EF108336, nt5328-nt6872), Ch2 (nt22445167-nt22443623). Sequences extracted from ensemble.org. Click here for file

Additional file 2 **Alignments of the EST sequences containing the complete region of Gau translation with homologous regions in mtDNA**. With the exception of the *Rattus norvegicus *sequence, the closest mtDNA issued from a complete genome has been used. Additionally, the nucleotide positions in the genome are given. The sequences corresponding to the *gau *regions are in bold letters. Characteristics of the sequences are the following: A) EST sequence from *Eucalyptus gunnii *(Viridiplantae, CT987850.1; another EST sequence (CT980201.1) is strictly identical to this one) and mtDNA sequence from *Carica papaya *(Viridiplantae, NC_012116); B) EST sequence from *Biomphalaria glabrata *(Mollusca, EE049639.1; the nucleotide insertion at position 531 is a sequencing artifact because it is not present in all ESTs from the *Biomphalaria *genus that contain this region) and mtDNA sequence from the same species (NC_005439); C) ESTs sequences from *Phlebotomus perniciosus *(Insecta, EST1: GW817739.1, EST2: GW816615.1, EST3: GW819720.1; for this last sequence, the 3' end has been removed because it apparently corresponds to a cloning artifact) and mtDNA sequence from *Anopheles darlingi *(NC_014275); D) ESTs sequences from *Mus musculus *and *Rattus norvegicus *(Muridae, BF784456.1 and CO394761.1, respectively) and mtDNA sequence from *Mus musculus *(NC_006914). Click here for file
