# Supplementary material for: Probable presence of an ubiquitous cryptic mitochondrial gene on the antisense strand of the cytochrome oxidase I gene
Source: Biol Direct. 2011 Oct 24;6:56. doi: 10.1186/1745-6150-6-56 (PMC3214167; doi:10.1186/1745-6150-6-56)
Supplement: Additional file 1 — Alignments of mammalian Gau proteins and cox1 regions. Characteristics of the nuclear gau sequences are shown in Table 1. For each species, the alignments of the nuclear Gau proteins have been provided with the sequences that have been deduced from the mitochondrial cox1 gene (mt.mt and mt.st are sequences that were translated using the standard and the mitochondrial (vertebrate) genetic codes, respectively). The alignments of the nuclear cox1-like regions are shown with those of the mitochondrial cox1 gene. For nuclear sequences, the chromosome number is shown after the two letters "Ch". The nuclear sequences corresponding to the gau regions are in bold. Characteristics of the nuclear sequences: Homo sapiens, cox1 (NC_001807, nt5905-nt7446), Ch1 (nt556317-nt557859), Ch14 (nt32023757-nt32022168); Pan troglodytes, cox1 (NC_001643, nt5321-nt6862), Ch2a (nt51808180-nt51806579), Ch8 (nt47844508-nt47845884); Pongo pygmaeus, cox1 (NC_001646, nt5331-nt6870), Ch2a (nt60553029-nt60554660); Macaca mulatta, cox1 (AY612638, nt5850-nt7391), Ch1 (nt108934590-nt108935852), Ch2 (nt123178799-nt123180392), Ch6(a) (nt30941431-nt30943006), Ch6(b) (nt50451345-nt50452956); Equus caballus, cox1 (EF597513, nt5359-nt6903), Ch27 (nt5205522-nt5203978); Canis familiaris, cox1 (U96639, nt5349-nt6893), Ch16 (nt9458239-nt9456847); Bos taurus, cox1 (NC_006853, nt5687-nt7231), Ch10 (nt4583738-nt4585281); Mus musculus, cox1 (EF108336, nt5328-nt6872), Ch2 (nt22445167-nt22443623). Sequences extracted from ensemble.org. [file 1745-6150-6-56-S1.DOC]

**A1 (human)**

H.sapiens-Ch1 MGSPPPAGSKKVVLRLRSVSSIVMPAARTGRDRRSKTAVIRTDQTKRGVW

H.sapiens-Ch14 IGSPPLAGSKKVVLRLWSVNSMVMPAARTGRDRRSKTAVIRTDQTKRGVW

H.sapiens-mt.st MGSPPPAGSKKVVLRLRSVSSIVMPAARTGRDRRSRTAVIRTDQTKRGVW

H.sapiens-mt.mt MGSPPPAGSKKVVL*LRSVSSMVMPAA*TG*D**S*TAVI*TDQTK*GVW

:**** ******** * **.*:***** ** . **** ***** ***

H.sapiens-Ch1 YWVMAGGFILIIVVMKLMAPKIEEIPARCKEKMVRSTEAPGWE*FPAKGG*

H.sapiens-Ch14 YWVMAGGFILMIVVMKLMAPRIEEMPARCKEKMVRSTEAPGWE*FPAKGG*

H.sapiens-mt.st YWVMAGGFILIIVVMKLMAPKIEETPARCKEKMVRSTEAPGWE*FPAKGG*

H.sapiens-mt.mt YWVMAGGFMLMIVVMKLMAPKMEETPA*CKEKMV*STEAPGWE*FPAKGG*

********:*:*********::** ** ****** ******** ******

**A2 (human)**

H.sapiens-Ch14 ATGTTCGCCGACCGTTGATTGTTCTCTACAAACCACAAAGACATTGGAACACTGTACCTA 60

H.sapiens-Ch1 ATGTTCGCCGACCGTTGACTATTCTCTACAAACCACAAAGACATTGGAACACTATACCTA 60

H.sapiens-cox1 ATGTTCGCCGACCGTTGACTATTCTCTACAAACCACAAAGACATTGGAACACTATACCTA 60

****************** * ******************************** ******

H.sapiens-Ch14 CTATTCGGCGCATGAGCTGGAGTCCTGGGCACAGCTCTAAGTCTCCTTATTTGGGCTGAG 120

H.sapiens-Ch1 TTATTCGGCGCATGAGCTGGAGTCCTAGGCACAGCTCTAAGCCTCCTTATTCGAGCCGAA 120

H.sapiens-cox1 TTATTCGGCGCATGAGCTGGAGTCCTAGGCACAGCTCTAAGCCTCCTTATTCGAGCCGAG 120

************************* ************** ********* * ** **

H.sapiens-Ch14 CTAGGCCAACCAGGCAACCTTCTAGGTAACGACCACATCTACAATGTCATCGTCACAGCC 180

H.sapiens-Ch1 CTGGGCCAGCCAGGCAACCTTCTAGGTAACGACCACATCTACAACGTTATCGTCACAGCC 180

H.sapiens-cox1 CTGGGCCAGCCAGGCAACCTTCTAGGTAACGACCACATCTACAACGTTATCGTCACAGCC 180

** ***** *********************************** ** ************

H.sapiens-Ch14 CATGCATTTGTAATAATCTTCTTCATAGTGATACCTATCATAATCGGAGGCTTTGGCAAC 240

H.sapiens-Ch1 CATGCATTTGTAATAATCTTCTTCATAGTAATACCCATCATAATCGGAGGCTTTGGCAAC 240

H.sapiens-cox1 CATGCATTTGTAATAATCTTCTTCATAGTAATACCCATCATAATCGGAGGCTTTGGCAAC 240

***************************** ***** ************************

H.sapiens-Ch14 TGACTAGTTCCCCTAAAAATCGGTGCCCCCGATATGGCATTCCCCCGCATAAACAACATA 300

H.sapiens-Ch1 TGACTAGTTCCCCTAATAATCGGTGCCCCCGATATGGCGTTTCCCCGCATAAACAACATA 300

H.sapiens-cox1 TGACTAGTTCCCCTAATAATCGGTGCCCCCGATATGGCGTTTCCCCGCATAAACAACATA 300

**************** ********************* ** ******************

H.sapiens-Ch14 AGCTTCTGACTCCTACCCCCTTCTCTCCTACTTCTGCTTGCATCTGCCATAGTGGAAGCC 360

H.sapiens-Ch1 AGCTTCTGACTCTTACCCCCCTCTCTCCTACTCCTGCTTGCATCTGCTATAGTGGAGGCC 360

H.sapiens-cox1 AGCTTCTGACTCTTACCTCCCTCTCTCCTACTCCTGCTCGCATCTGCTATAGTGGAGGCC 360

************ **** ** *********** ***** ******** ******** ***

H.sapiens-Ch14 GGCGCCGGAACAGGTTGGACAGT**CTACCCTCCCTTAGCAGGGAACTACTCCCACCCTGGA** 420

H.sapiens-Ch1 GGCGCAGGAACAGGTTGAACAGT**CTACCCTCCCTTGGCAGGGAACTACTCCCACCCTGGA** 420

H.sapiens-cox1 GGAGCAGGAACAGGTTGAACAGTCTACCCTCCCTTAGCAGGGAACTACTCCCACCCTGGA 420

** ** *********** ***************** ************************

H.sapiens-Ch14 **GCTTCCGTAGATCTAACCATCTTCTCCTTGCATCTAGCAGGCATCTCCTCTATCCTAGGA** 480

H.sapiens-Ch1 **GCCTCCGTAGACCTAACCATCTTCTCCTTACACCTAGCAGGTATCTCCTCTATCTTAGGA** 480

H.sapiens-cox1 GCCTCCGTAGACCTAACCATCTTCTCCTTACACCTAGCAGGTGTCTCCTCTATCTTAGGG 480

** ******** ***************** ** ******** *********** ****

H.sapiens-Ch14 **GCCATTAACTTCATTACAACAATCATTAATATAAAACCCCCTGCCATAACCCAATACCAA** 540

H.sapiens-Ch1 **GCCATCAATTTCATCACAACAATTATTAATATAAAACCCCCTGCCATAACCCAATACCAA** 540

H.sapiens-cox1 GCCATCAATTTCATCACAACAATTATCAATATAAAACCCCCTGCCATAACCCAATACCAA 540

***** ** ***** ******** ** *********************************

H.sapiens-Ch14 **ACACCCCTTTTCGTCTGATCCGTCCTAATCACAGCAGTCTTACTTCTCCTATCCCTCCCA** 600

H.sapiens-Ch1 **ACGCCCCTTTTCGTCTGATCCGTCCTAATCACAGCAGTCTTACTTCTCCTATCTCTCCCA** 600

H.sapiens-cox1 ACGCCCCTCTTCGTCTGATCCGTCCTAATCACAGCAGTCCTACTTCTCCTATCTCTCCCA 600

** ***** ****************************** ************* ******

H.sapiens-Ch14 **GTCCTAGCTGCTGGCATCACCATGCTGTTAACAGACCATAACCTCAACACCACCTTCTTC** 660

H.sapiens-Ch1 **GTCCTAGCCGCTGGCATCACTATACTACTAACAGACCGTAACCTCAACACCACCTTCTTC** 660

H.sapiens-cox1 GTCCTAGCTGCTGGCATCACTATACTACTAACAGACCGCAACCTCAACACCACCTTCTTC 660

******** *********** ** ** ********* *********************

H.sapiens-Ch14 **GACCCAGCCAGGGGAGGGGACCCTAT**TCTATACCAACACCTATCCCTTGCAGGGACATGG 720

H.sapiens-Ch1 **GACCCAGCCGGAGGAGGAGACCCCAT**TCTATACCAACACCTATTCT-----GATTTTTCG 715

H.sapiens-cox1 GACCCCGCCGGAGGAGGAGACCCCATTCTATACCAACACCTATTCT-----GATTTTTCG 715

***** *** * ***** ***** ******************* * * * *

H.sapiens-Ch14 ATAATGCTGGAAACCATCATTCTCAGCAAACTAACACAGGAACAGAAAACCAAACACCAC 780

H.sapiens-Ch1 GTCACCCTGAAGTTTAT-ATTCTCATCCTAC-----CAGGCTTCGGAAT---AATCTCCC 766

H.sapiens-cox1 GTCACCCTGAAGTTTAT-ATTCTTATCCTAC-----CAGGCTTCGGAAT---AATCTCCC 766

* * *** * ** ***** * * ** **** * ** ** * *

H.sapiens-Ch14 ATGTTCTCACTTATAAGTGGGAGTTGAACAATGAGAACATATGGACACAGGGAGGGGAAC 840

H.sapiens-Ch1 ATATTGTAACTTACTACTCCGGG---AAAAAAAGAACCATTTGGATACATAGG------- 816

H.sapiens-cox1 ATATTGTAACTTACTACTCCGG----AAAAAAAGAACCATTTGGATACATAGG------- 815

** ** * ***** * * * ** ** * *** **** *** *

H.sapiens-Ch14 ATACACACCAGGGTCTGTCAGGGGATGGGGGGCAAGGTGAGGGACAGCATTAGGAGAAAT 900

H.sapiens-Ch1 -TATGGTCTGAGCTATGATATCAATTGGCTTCCTAGG----GTTTATCGT--GTGAGCAC 869

H.sapiens-cox1 -TATGGTCTGAGCTATGATATCAATTGGCTTCCTAGG----GTTTATCGT--GTGAGCAC 868

** * * * ** * *** * *** * * * * * *

H.sapiens-Ch14 ACCTAATGTAGACGACAGGTTGATGGGTGCAGCCACCACCATAGCCCATGTATACTATGT 960

H.sapiens-Ch1 ACCATATATTTACAGTAGGAATAGACGTAGACACACGAGCATATTTCACCTCCGCTACC- 928

H.sapiens-cox1 ACCATATATTTACAGTAGGAATAGACGTAGACACACGAGCATATTTCACCTCCGCTACC- 927

*** ** * ** *** * ** * *** * **** ** * ***

H.sapiens-Ch14 AACAAACCTGCACGTTCTGCATATGTATCCCAGAACTTAAAGTACAATAAATAAATCTGG 1020

H.sapiens-Ch1 -ATAATCAT-CGCTATCCCCACCGGCGTCAAAGTATTTAGCTGACTCGCCACACTCCACG 986

H.sapiens-cox1 -ATAATCAT-CGCTATCCCCACCGGCGTCAAAGTATTTAGCTGACTCGCCACACTCCACG 985

* ** * * * * ** ** * ** ** * *** ** * * * *

H.sapiens-Ch14 GGGGCACAGTGGCTCA--CACCTGTAAT-CCCAGAACTTTGGGAGGCTGAGGCATGCAGA 1077

H.sapiens-Ch1 GAAGCAATATGAAATGATCTGCTGCAGTGCTCTGAGCCCTAGGA---TTTATTTTTCTTT 1043

H.sapiens-cox1 GAAGCAATATGAAATGATCTGCTGCAGTGCTCTGAGCCCTAGGA---TTCATCTTTCTTT 1042

* *** ** * *** * * * * ** * * *** * * *

H.sapiens-Ch14 TCACCTGAGGTTGGGAGTTCAAGACCAGCCTGACCAAC--ATGGAGAAACCCCGT-CTCC 1134

H.sapiens-Ch1 TCACCGTAGGTGGCCTGACTGGCATTGTATTAGCAAACTCATCACTAGACATCGTACTAC 1103

H.sapiens-cox1 TCACCGTAGGTGGCCTGACTGGCATTGTATTAGCAAACTCATCACTAGACATCGTACTAC 1102

***** **** * * * * * *** ** * ** *** ** *

H.sapiens-Ch14 ACTAAAAATACAAAATTAGCTGGGAGTGGTGGCATATGCCTGTAATCCCAGCTACTCAGG 1194

H.sapiens-Ch1 ACGACACGTACTACGTTGTAGCCCACTTCCACTATGTCCTATCAATAGGAGCTGTATTTG 1163

H.sapiens-cox1 ACGACACGTACTACGTTGTAGCTCACTTCCACTATGTCCTATCAATAGGAGCTGTATTTG 1162

** * * *** * ** * * ** * * *** **** *

H.sapiens-Ch14 AGGCTGAGGCAGGAGAATTGCTTGAACCGGGGAGGCGGAGGTTGCAGTGAGCCGAGATCA 1254

H.sapiens-Ch1 ---CCATCATAGGAG----GCTTCATTCACTGAT-------TTCCCCTATTCTCAGGCTA 1209

H.sapiens-cox1 ---CCATCATAGGAG----GCTTCATTCACTGAT-------TTCCCCTATTCTCAGGCTA 1208

* ***** **** * * ** ** * * * ** *

H.sapiens-Ch14 TGCCATTGTGCTCCAGCCTGGGCAAAAAG--AGCGAAACTCCAT-CTCATAAAAAATAAA 1311

H.sapiens-Ch1 CACCCTAGA--CCAAACCTACGCCAAAATCCATTTCGCTATCATATTCATCGGCGTAAAT 1267

H.sapiens-cox1 CACCCTAGA--CCAAACCTACGCCAAAATCCATTTCACTATCATATTCATCGGCGTAAAT 1266

** * * * * *** ** **** * *** **** **

H.sapiens-Ch14 TAAATAATTTTTTTAAAAAAGACACAAAACCTATAAGAGAAAAAAAATCAAACTTCACT- 1370

H.sapiens-Ch1 CTAACTTTCTTCCCACAACACTTTCTCGGCCTATCCGG----AATGCCCCGACGTTACTC 1323

H.sapiens-cox1 CTAACTTTCTTCCCACAACACTTTCTCGGCCTATCCGG----AATGCCCCGACGTTACTC 1322

** * ** * ** * * ***** * ** * ** * ***

H.sapiens-Ch14 ATAAAACTGTTTTACATTC-CTACTTGAAATAGAAAATG--CTATAAACAAAGTTAAAAG 1427

H.sapiens-Ch1 GGACTATCCCGATGCATACACCACATGAAATATCCTATCATCTGTAGGCTCATTCATTTC 1383

H.sapiens-cox1 GGACTACCCCGATGCATACACCACATGAAACATCCTATCATCTGTAGGCTCATTCATTTC 1382

* * * *** * * ** ***** * ** ** ** * * * *

H.sapiens-Ch14 ATAAACAGCAA-ACCAAGAAAAGTATTTGCAAATATGCACACTATTTTCTACAAATCAAA 1486

H.sapiens-Ch1 TCTAACAGCAGTAATATTAATAATTTTCATAATT-TGAGAAGCCTTCGCTTCGAAGCGAA 1442

H.sapiens-cox1 TCTAACAGCAGTAATATTAATAATTTTCATGATT-TGAGAAGCCTTCGCTTCGAAGCGAA 1441

******* * * ** * * ** * * ** * ** ** * ** * **

H.sapiens-Ch14 AAGACTGGACAAAGTTCACAAAACCTTCATAAAGAAAAAGTATTATAATAAAGATTATAC 1546

H.sapiens-Ch1 AAGTCCTAATA--GTAGAAGAACCCTCCATAAACCTGGAGTGACTATATGGATGCCCCCC 1500

H.sapiens-cox1 AAGTCCTAATA--GTAGAAGAACCCTCCATAAACCTGGAGTGACTATATGGATGCCCCCC 1499

*** * * * ** * ** *** ****** *** ** * *

H.sapiens-Ch14 AAAAGAGAATACAACTGCCTTTTAAATATATGAAAATATTTTCA 1590

H.sapiens-Ch1 ACCCTACCACACATTCGAAGAACCCGTATACATAAA-ATCTAGA 1543

H.sapiens-cox1 ACCCTACCACACATTCGAAGAACCCGTATACATAAA-ATCTAGA 1542

* * * *** * **** *** ** * *

**B1 (chimpanzee)**

P.troglodytes-Ch8 IGSPPPAGSKKVVLRLRSVNNIVMPAARTGRDRRSKTAVIRTDQMKRGVW

P.troglodytes-Ch2a IGSPPPTGSKKVVSRLWSVNNIVMPAARTGRERRSRTAVIRTDQTKRGVW

P.troglodytes-mt.st IGSPPPAGSKKVVLRLRSVNSMVMPAARTGRDRRSKTAVIRTDQTKRGVW

P.troglodytes-mt.mt MGSPPPAGSKKVVL*LRSVNSMVMPAA*TG*D**SKTAVI*TDQTK*GVW

:*****:****** * ***.:***** ** : *:**** *** * ***

P.troglodytes-Ch8 YWVMAGGFILMIVVMKLMAPRIEEMPARCREKMVRSTEAPG*E*FPAKGG*

P.troglodytes-Ch2a Y*DMAGGFMLIIVIMKLIAPGMEEIPARCREKMVRSTEAPGWE*FPAKEG*

P.troglodytes-mt.st YWVMAGGFILIIVVMKLMAPRIEEMPARCKEKMVRSTEAPGCE*FPAKGG*

P.troglodytes-mt.mt YWVMAGGFMLMIVVMKLMAP*MEEMPA*CKEKMV*STEAPGCE*FPAKGG*

* *****:*:**:***:** :**:** *:**** ****** ***** *

**B2 (chimpanzee)**

P.troglodytes-Ch8 NNNNNNNNNNNNNNNNNNNNNNNNNNNNNNNNNNNNNNNNNNNNNNNNNNNNNNNNNNNN 60

P.troglodytes-Ch2a CTCTACCCAGCTCCCTGACCTTGGGCAAGACACAGTCTTTTGGTGTGTCTGGCTATAACA 60

P.troglodytes-cox1 ATGTTCACCGACCGCTGACTATTCTC----TACAAACCACAAAGATATTGGAACACTATA 56

P.troglodytes-Ch8 NNNNNNNNNNNNNNNNNNNNNNNNNNNNNNNNNNNNNNNNNNNNNNNNNNNNNNNNNNNN 120

P.troglodytes-Ch2a GTTGGGTTTTGCTGCAAACCTACTTAACTCCAGTGAGCCTCGATTTCCTCATTGGTAAAG 120

P.troglodytes-cox1 CCTACTATTCGGTGCATGAGCTGGAGTCCTGGGCACAGCCCTAAGTCTCCTTATTCGGGC 116

P.troglodytes-Ch8 NNNNNNNNNNNNNNNNNNNNNNNNNNNNNNNNNNNNAAATATTTGAGTGATGTTTGTTTC 180

P.troglodytes-Ch2a TGAGCATAAGAATCCCTGTTTTGGCCAGACATGGTGGCTCACACCTGTAATCCCAACTCT 180

P.troglodytes-cox1 TGAAC-TAGGCCAACCAGGCAACCTCCTAGGTAATGAC-CACATCTACAATGTC-ATCGT 173

* **

P.troglodytes-Ch8 CAGGGTGACTCAAATTGAATCAACACTTTGGA-TTGGTTTCAGATCTTTCCTTTGAGGAA 239

P.troglodytes-Ch2a TTGGGAGGCTGATGTGGGCAGATCACTTGAGAACAGGTGTTTGAGACCAACC----TGGG 236

P.troglodytes-cox1 CACAGCCCATGCATTCGTAATAATCTTCTTCA-TAGT-----AATGCCTATTATAATCGG 227

* * * * * * * * *

P.troglodytes-Ch8 AAAAATTGTGAAATG---AGTACAGTT-----TT----AATGTTCAGGAAAGATTGGCTT 287

P.troglodytes-Ch2a CAACATGGTGAAACA-CCGTCTCCACTAAGAATACAAAAATTAGCCAGGCATGGTGGTGT 295

P.troglodytes-cox1 AGGCTTTGGCAACTGGCTAGTTCCCTTGATAATT----GGTGCCCCCGACA---TGGCAT 280

* * ** * * * * * * * *** *

P.troglodytes-Ch8 TGTCCT------TAAGGAAATTA---TATTGATTAGAA---TTTCTTTAAAACTAATTTA 335

P.troglodytes-Ch2a ACACCTGTGGTCCTAGCTACTCAGGAGGCTGAGGAGGGAGGCTGACTTGAGCCCAGGATG 355

P.troglodytes-cox1 TCCCCCGCA---TAAACAACATAAGCTTCTGGCTCCTG---CCCCCTTCTCTCCTACTTC 334

** * * * ** ** * *

P.troglodytes-Ch8 ---AAGTGACACTCACTTT--TGATAAC--------GAACATGTTGGACAGT**CTACCCTC** 382

P.troglodytes-Ch2a GGGAGGTTGCAGTGAGCCA--TGTCCACC-----TGGAACGGGCTGGACAGT**TTATCCTT** 408

P.troglodytes-cox1 ---TACTTGCATCTGCCATAGTAGAAGCCGGCGCGGGAACAGGTTGAACAGTCTACCCTC 391

* ** * * **** * ** ***** ** ***

P.troglodytes-Ch8 **CCTTGGCAGGAAATTACTCTCATCCTGGAGCTTCTGTAGATCTAACCATTTTCTCCCTGC** 442

P.troglodytes-Ch2a **CCTTAGCAGGAAACTACTCCCACCCAGGAGCCTCTGTAGACCTGACCATTTTCTCCCTGC** 468

P.troglodytes-cox1 CCTTAGCGGGAAACTACTCGCATCCTGGAGCCTCCGTAGACCTAACCATCTTCTCCTTAC 451

**** ** ***** ***** ** ** ***** ** ***** ** ***** ****** * *

P.troglodytes-Ch8 ACCTAGCAGGCATCTCCTCTATCCTAGGGGCCATCAACTTCATTACAACAATCATCAATA 502

P.troglodytes-Ch2a **ATCTAGCAGGTATCTCCTCCATTCCAGGGGCTATTAACTTCATTATGACAATTATTAACA** 528

P.troglodytes-cox1 ATCTGGCAGGCATCTCCTCTATCCTAGGAGCCATTAACTTCATCACAACAATTATTAATA 511

* ** ***** ******** ** * *** ** ** ******** * ***** ** ** *

P.troglodytes-Ch8 **TAAAACCCCCTGCCATAACCCAATACCAAACACCCCTTTTCATCTGATCCGTCCTAATCA** 562

P.troglodytes-Ch2a **TAAAACCCCCTGCCATATCTCAATACCAAACACCCCTCTTTGTCTGATCCGTCCTAATCA** 588

P.troglodytes-cox1 TAAAACCTCCTGCCATGACCCAATACCAAACACCCCTCTTCGTCTGATCCGTCCTAATCA 571

******* ******** * ***************** ** ******************

P.troglodytes-Ch8 **CAGCAGTCTTGCTTCTTCTATCCCTCCCAGTCCTAGCTGCTGGCATTACAATGTTGTTAA** 622

P.troglodytes-Ch2a **CAGCAGTCCTACTCCTCCTCTCCCTTCCAGTCCTGGCCGCTGGCATTACTATATTATTAA** 648

P.troglodytes-cox1 CAGCAGTCTTACTTCTCCTATCCCTCCCAGTCCTAGCTGCTGGCATCACCATACTATTGA 631

******** * ** ** ** ***** ******** ** ******** ** ** * ** *

P.troglodytes-Ch8 **CAGACCGTAACCTCAATACCACCTTCTTCGACCCAGCTGGAGGAGGAGACCCTAT**TCTAT 682

P.troglodytes-Ch2a **CAGACCACAACCTTGATACCACCTTCTTTGATCCAGTCGGAGGGGGAGACCCTAT**CCTGT 708

P.troglodytes-cox1 CAGATCGTAACCTCAACACTACCTTCTTCGACCCAGCCGGGGGAGGAGACCCTATTCTAT 691

**** * ***** * ** ******** ** **** ** ** *********** ** *

P.troglodytes-Ch8 ACCAACACCTATTCTGATTTTTTGGTCACCCCGAAGTTTATATTCTTATCCTGCCCGGCT 742

P.troglodytes-Ch2a ACCAACACCTATTTTGATTCTTCGGCCATCCCGAAGTCTATATTCTCATCCTACCAGGCT 768

P.troglodytes-cox1 ATCAACACTTATTCTGATTTTTTGGCCACCCCGAAGTTTATATTCTTATCCTACCAGGCT 751

* ****** **** ***** ** ** ** ******** ******** ***** ** ****

P.troglodytes-Ch8 TTGGAATAATTTCCCACATTGTAACTTACTACTCCGGAAAAAAAGAGCCATTCGGGTACA 802

P.troglodytes-Ch2a TCGGGATAATTTCCCACATCGTAACATACTATTCTGGAAAAAAAGAACCATTCGGGTATA 828

P.troglodytes-cox1 TCGGAATAATTTCCCACATTGTAACTTATTACTCCGGAAAAAAAGAACCATTTGGATATA 811

* ** ************** ***** ** ** ** *********** ***** ** ** *

P.troglodytes-Ch8 TAGGTATGGTCTGGGCTATAATATCAATTGGTTTCCTAGGGTTTATTGTGTGAGCGCACC 862

P.troglodytes-Ch2a TGGGCATAGTCTGAGCTATGAAATCAATTGGTTTCCTAGGATTTATTGTATGAGCTCATC 888

P.troglodytes-cox1 TAGGCATGGTTTGAGCTATAATATCAATTGGCTTCCTAGGGTTTATCGTGTGAGCACACC 871

* ** ** ** ** ***** * ********* ******** ***** ** ***** ** *

P.troglodytes-Ch8 ATATGTTTACAGTAGGAATAGACGTAGACACACGAGCCTACTTCACCTCCGCTACCATAA 922

P.troglodytes-Ch2a ATATATTCACAGTAGGGATAGACGTGGATACACGAGCCTACTTCACCTCCGCTACTATCA 948

P.troglodytes-cox1 ATATATTTACAGTAGGGATAGACGTAGACACCCGAGCCTATTTCACCTCCGCTACCATAA 931

**** ** ******** ******** ** ** ******** ************** ** *

P.troglodytes-Ch8 TCATCGCTATTCCCACCGGCGTCAAAGTATTTAGCTGACTCGCTACACTCCACGGAAGCA 982

P.troglodytes-Ch2a TTATCGCCATTTCCACTGGCGTCAAACTATTTAGTTGACTTGCTACACTTCACAGAAGTA 1008

P.troglodytes-cox1 TCATTGCTATTCCTACCGGCGTCAAAGTATTCAGCTGACTCGCTACACTTCACGGAAGCA 991

* ** ** *** * ** ********* **** ** ***** ******** *** **** *

P.troglodytes-Ch8 ATACCAAATGATCTGCTGCAGTACTCTGAGCCCTAGGGTTCATTTTTCTCTTCACTGTAG 1042

P.troglodytes-Ch2a ACATCAAATGATCTGCCGCAGTACTCTGAGCCGTAGGGTTCATCTTCCTCTTCACTGTGG 1068

P.troglodytes-cox1 ATATGAAATGATCTGCCGCAGTACTCTGAGCCCTAGGGTTTATCTTTCTCTTCACCGTAG 1051

* * *********** *************** ******* ** ** ******** ** *

P.troglodytes-Ch8 GTGGCCTAACCGGCATCGTACTAGCAAACTCATCATTAGATATCATACTGCACGATACAT 1102

P.troglodytes-Ch2a GTAGTCTAACCGGCATTGTACTAGCAAACTTATCCTTAGATATCTAATGATGAAATATA- 1127

P.troglodytes-cox1 GTGGCCTAACCGGCATTGTACTAGCAAACTCATCATTAGACATCGTGCTACACGACACAT 1111

** * *********** ************* *** ***** *** * * *

P.troglodytes-Ch8 ATTACGTCGTAGCTCACTTCC----ATTATGTCCTATC---AATAGG-GGCTGTATTCGC 1154

P.troglodytes-Ch2a ACTAATTTCTAATTAATCTGAGAAAATTAAGACTCAGAGAAAATAGGTAACTAGGTAAAC 1187

P.troglodytes-cox1 ACTACGTCGTAGCCCACTTCC----ACTACGTTCTATC---AATAGG-AGCTGTATTCGC 1163

* ** * ** * * * ** * * ****** ** * *

P.troglodytes-Ch8 CATCATAGGGGGTTTTATCCACTGATTTCCCTTATTCTCAGG---CTACACTCTG----G 1207

P.troglodytes-Ch2a TCCAAAAAGCCAATAAATAGACAAGAACAGAGTAGAACAGAAATACTATAACTTACTGAA 1247

P.troglodytes-cox1 CATCATAGGAGGCTTCATTCACTGATTCCCCCTATTCTCAGG---CTATACCCTA----G 1216

* * * * ** ** ** *** * *

P.troglodytes-Ch8 ACCAAACCTATGCCAAAATCCATTTTGCCATCATGTTCATTGGCGTA--AATCTAACCTT 1265

P.troglodytes-Ch2a GGCACGTTTTTACCATTACACATTTTTCAGTAATGGATGAAGTCGGAGGAGTCAGACATT 1307

P.troglodytes-cox1 ACCAAACCTATGCCAAAATCCAATTTGCCATCATGTTCATTGGCGTA--AACCTAACCTT 1274

** * * *** * ** *** * * *** * ** * * * ** **

P.troglodytes-Ch8 CTTCCCACAACACTTTCT-TGGCTTGTCTGGAATACCCCGACGATACTCGGACTACCCCG 1324

P.troglodytes-Ch2a AAGCACTCTAGAGTTCCTGTTAGTTGCTTCCTATACTGTCACAATGTTTATACACACTGT 1367

P.troglodytes-cox1 CTTCCCACAGCACTTCCT-TGGCCTATCTGGGATGCCCCGACGTTACTCGGACTACCCCG 1333

* * * * ** ** * * * ** * ** * * ** *

P.troglodytes-Ch8 ATGCATACAACACA--------TGAAACATTCTATC--------------ATCTATGGGC 1362

P.troglodytes-Ch2a ATATGTTCTACACACTTTCCATTAAAATATATGATTTCATGAGGTGCAGAATCTTTGAGC 1427

P.troglodytes-cox1 ATGCATACACCACA--------TGAAATGTCCTATC--------------ATCCGTAGGC 1371

** * * **** * *** * ** *** * **

P.troglodytes-Ch8 TCATTTATTTCCCTAACAGCAGTAATATTAATAATTTTTATAAT------TTGAGAAGCC 1416

P.troglodytes-Ch2a TCTGTCAAAGACCCAATGTCACAGTTACTATTTAGTTTAATAATGTTATTTTTATTATTT 1487

P.troglodytes-cox1 TCATTTATCTCCCTGACAGCAGTAATATTAATAATTTTCATGAT------TTGAGAAGCC 1425

** * * ** * ** ** ** * * *** ** ** ** * *

P.troglodytes-Ch8 TTCGCTTCAAAACGAAAAGTCCTAATAATCGAAGAACCCTCCACAAACCTGGAGTGGCTA 1476

P.troglodytes-Ch2a CCTGTTCATAAGGCTCAGATTCTATTTTTCCACAGTATTGCCATAG----GGAGCCATCA 1543

P.troglodytes-cox1 TTTGCTTCAAAACGAAAAGTCCTAATAGTAGAAGAGCCCTCCGCAAACCTGGAATGACTA 1485

* * ** * * *** * * * ** * *** *

P.troglodytes-Ch8 TATGGATGCCCCCTACCCTA--TCATACATTCGAAGAACCCGTATACATAAAATCTAGA 1533

P.troglodytes-Ch2a GGAGCATGTTAAAAAAAGCAGCTTATTCATTTGCGGAACTTGCCTTTTGGTTTTTTAAA 1602

P.troglodytes-cox1 TATGGATGCCCCCCACCCTA--CCACACATTCGAAGAACCCGTATACATAAAATCTAGA 1542

* *** * * * **** * **** * * * ** *

**C1 (**[**Orangutan**](http://www.ensembl.org/Pongo_pygmaeus/Info/Index/)**)**

P.pygmaeus-Ch2a IGSPLPAGSKKVVLRLLSVNNIVMPVARTGRERRSRAAVIRTDQTKRGVW

P.pygmaeus-mt.st IGSPPPAGSKNVVFKLRSVSSMVMPAARTGRERRSRTAVIRIDQTK*RGV

P.pygmaeus-mt.mt MGSPPPAGSKNVVFKLRSVSSMVMPAA*TG*E**S*TAVI*IDQTK*GVW

:*** *****:**::* **..:***.* ** * * :*** ****

P.pygmaeus-Ch2a YRDMAGGFMLIIVVMKLIAPRMEEIPARCREKMVRSTEVPGWE*FPAKGG*

P.pygmaeus-mt.st YWDIAGGFILIIVVMKLIAPRIEEMPARCREKIVKSTEAPG*E*FPARGG*

P.pygmaeus-mt.mt YWDIAGGFMLMIVVMKLMAP*IEEMPA*C*EKIVKSTEAPGWE*FPA*GG*

* *:****:*:******:** :**:** * **:*:***.** * *** **

**C2 (**[**Orangutan**](http://www.ensembl.org/Pongo_pygmaeus/Info/Index/)**)**

P.pygmaeus-Ch2a GCATTTGCAGTTCCAATGCCAATCTTGTCTCTACCCAGCTCCCTGACCTTGGG-CGAGAC 59

P.pygmaeus-cox1 ATGTTCGCCGA-CCGCTGGCTAT----TCTCCACGAACCACAAAGATATTGGAACGCTAT 55

** ** * ** ** * ** **** ** * * * ** **** ** *

P.pygmaeus-Ch2a ACA-ATCTTTTGGTGTGTCTGGCTATAACGGTTGGGTTTTGCTGCAAACCTACTTAACTC 118

P.pygmaeus-cox1 ACCTGTTGTTCGGCGCATGAGCTGGTGTC—CTAGGCACTGCCCTAAGCCTCCTCATTCG 113

** * ** ** * * * * * * ** *** ** *** ** *

P.pygmaeus-Ch2a CAATGAGCCTCGATTTCCTCATTGGTGAAGTGTGCATAAGAATCCCTGTTTTGGCCAGG- 177

P.pygmaeus-cox1 TGCTGAAC-TAGGCCAACCCG-----GCAACCTCCTAGGTAATGACCATATTTACAATGT 167

*** * * * * * * * * * *** * * ** * * *

P.pygmaeus-Ch2a CATGGTGGCTCACACCTGTAATCCCAACTATTTGGGAGGCTGATGTGGGCAGATCACTTG 237

P.pygmaeus-cox1 CATCGTCACA-GCCCATGCATTCGTAATAATTT------TTTTCATGGTCATGCC-CATA 219

*** ** * * * ** * ** ** **** * *** ** * * *

P.pygmaeus-Ch2a AGAACAGGAGTTTGAGACCAACGTGGGCAACATGGTGAAACACCGTCTCCACTAAAAATA 297

P.pygmaeus-cox1 ATAATTGGAGGCTTTGGC-AAC-TGACTAGTGCCCCTGATAATTGGCGCCCCTGATATGG 277

* ** **** * * * *** ** * * * * * ** ** * *

P.pygmaeus-Ch2a CAAAAATTAGCCAGGCATGG-TGGTGTACACCTGTGGTCCTAGCTACTCAGGAGGCTGAG 356

P.pygmaeus-cox1 CA-----TTCCCGCGCATAAATAACATAAGCTTCTGACTCCTCCTCCCCTCCTTCCTCCT 332

** * ** **** * ** * * ** * ** * * **

P.pygmaeus-Ch2a GAGGGAGGCTCACTTGAGCCCAGGACGG-GGAGGTTGCAGTGAGCCATGTCCACCCGGAA 415

P.pygmaeus-cox1 A--------TTACTCGCTTCTGCTACAGTAGAGGCCGGAGCA--------------GGAA 370

* *** * * ** * **** * ** ****

P.pygmaeus-Ch2a CAGGCTGGACAGT**TTATCCTCCCTTAGCGGGAAACTACTCCCACCCAGGAACCTCTGTAG** 475

P.pygmaeus-cox1 CGGGCTGAACAGTCTATCCACCCCTAGCAGGAAACTACTCTCACCCAGGAGCCTCTGTAG 430

* ***** ***** ***** *** **** *********** ********* *********

P.pygmaeus-Ch2a **ACCTGACCATTTTCTCCCTGCATCTGGCAGGTATCTCCTCCATTCTAGGGGCTATTAACT** 535

P.pygmaeus-cox1 ACTTGACAATCTTCTCTCTACACCTAGCAGGCATTTCCTCAATTCTAGGGGCTATCAATT 490

** **** ** ***** ** ** ** ***** ** ***** ************** ** *

P.pygmaeus-Ch2a **TCATTACGACAATTATTAACATAAAACCCCCTGCCATATCTCGATACCAAACACCCCTCT** 595

P.pygmaeus-cox1 TCATTACAACAATTATTAATATAAAACCCCCTGCAATATCCCAATATCAAACTCCCCTCT 550

******* *********** ************** ***** * *** ***** *******

P.pygmaeus-Ch2a **TTGTCTGATCCGTCCTAATCACAGCAGCCCTACTCCTCCTCTCCCTTCCAGTCCTGGCCA** 655

P.pygmaeus-cox1 TCGTCTGATCAATCCTGATCACAGCAGTCCTACTTCTCCTCTCCCTCCCAGTCCTAGCCG 610

* ******** **** ********** ****** *********** ******** ***

P.pygmaeus-Ch2a **CTGGCATTACTATGTTATTAACAGACAGCAACCTTAATACCACCTTCTTTGATCCAGCTG** 715

P.pygmaeus-cox1 CTGGCATCACCATACTACTAACAGACCGCAACTTAAATACTACATTCTTTGACCCGGCTG 670

******* ** ** ** ******** ***** * ***** ** ******** ** ****

P.pygmaeus-Ch2a **GAAGGGGAGACCCTAT**CCTGTACCAACACCTATTTTGATTTTTCAGTCACCCTGATGTCT 775

P.pygmaeus-cox1 GAGGTGGGGATCCTATCCTATACCAACACTTATTCTGATTTTTCGGCCACCCTGAAGTCT 730

** * ** ** ******** ********* **** ********* * ******** ****

P.pygmaeus-Ch2a ATATTCTCATCCTACCGGGCTTCGGGATAATTTCCCACATCGTAACATACTATTCTG-AA 834

P.pygmaeus-cox1 ACATTCTCATCCTACCAGGTTTCGGCATAATCTCCCACATCGTAACACACTACTCCGGAA 790

* ************** ** ***** ***** *************** **** ** * **

P.pygmaeus-Ch2a AAAAAGAACCATTCGGGTATATGGGCATAGTCTGAGCTATGATGTCAGTTGGTTTCCTAG 894

P.pygmaeus-cox1 AAAAAGAACCATTTGGGTATATAGGCATAGTCTGAGCCATAGTCTCAATTGGTTTCCTGG 850

************* ******** ************** ** * *** ********** *

P.pygmaeus-Ch2a GATTTATTATATGAGCTCATCATATATTCACAGTAGGAATAGATGTGGATACACGAGCCT 954

P.pygmaeus-cox1 GTTTTATCGTATGAGCCCACCACATATTCACAGTAGGGATAGACGTGGACACACGAGCCT 910

* ***** ******* ** ** ************** ***** ***** **********

P.pygmaeus-Ch2a ACTTCACCTCCACTACTATAATTATCGCCATTCCCACTGGCGTCAAAGTATTTAGTTGAC 1014

P.pygmaeus-cox1 ACTTCACCTCCGCTACCATAATTATTGCCATCCCCACCGGCGTCAAAGTATTTAGCTGAC 970

*********** **** ******** ***** ***** ***************** ****

P.pygmaeus-Ch2a TTGCTACGCTTCACGGAAGAAACACCAAATGATCTGCTGCAATACTCTGAGCCCTAGGGT 1074

P.pygmaeus-cox1 TCGCTACACTCCACGGAAGCAACACTAAATGATCTGCCGCAATCCTCTGAGCCTTAGGAT 1030

* ***** ** ******** ***** *********** ***** ********* **** *

P.pygmaeus-Ch2a TCATCTTCCTCTTCACTGTGGGCGGTCTAACCGGCATTGTACTAGCAAACTCATCATTAG 1134

P.pygmaeus-cox1 TCATTTTCCTCTTCACCGTAGGCGGCTTAACAGGCATCGTACTGGCAAACTCATCACTAG 1090

**** *********** ** ***** **** ***** ***** ************ ***

P.pygmaeus-Ch2a ATATCTAATGATGAAATATA-ACTGATTTTTAATTAATCTGAGAAAATGAAGACTCAGAG 1193

P.pygmaeus-cox1 ACATCGTATTACACGATACATACTACGTTGTAGCCCACTTTC----ACTACGTCTTAT-- 1144

* *** ** * *** * *** ** ** * * * * * ** *

P.pygmaeus-Ch2a AAAATAGGTAACTAAGTAAACTCCAAAAAGCCAATAAATAGACAAGAACAGACTAGAACA 1253

P.pygmaeus-cox1 -CAATAGG-AGCTGTATTCGCCATCATAGGAGGCTTCATCCACTGGTTCCCACTATTCTC 1202

****** * ** * * * * * * ** ** * * ****

P.pygmaeus-Ch2a GAAATACTATAACTTACTGAAGGCACGTTTTTACCATTACACATTTTTCAGTAATGGATG 1313

P.pygmaeus-cox1 AG---GCTACACCTTA----AACCAGACCTATGCTAAAATTCACTTCATCACCATATTTG 1255

*** * **** * ** * * * * * ** ** ** **

P.pygmaeus-Ch2a AAGTCAGACGAGTCAGATATTAAGCACTCTAGAGTTCCTGTTAGTTATTTCCTATACTGT 1373

P.pygmaeus-cox1 TCGGCG--TAAATTTAACCTTCTTCCCGCAACATTTCCT-TGGCCTATCAGGTATACCCC 1312

* * * * * ** * * * * * ***** * *** *****

P.pygmaeus-Ch2a CACAATGTTTATAAGCACTGTATATGTTCTACACACTTCCCATTAAAATATATGATTTCA 1433

P.pygmaeus-cox1 GACGCTACTC-CGATTACC--------CCGACGCATATACCAC----ATGAAATATTTTA 1359

** * * * ** * ** ** * *** ** * **** *

P.pygmaeus-Ch2a TGAGGTGCAGAATCTTTGAGCTCTGTCAAAGATCCAATGTCACAGTTACTATTTAGTGTA 1493

P.pygmaeus-cox1 TCATCCGCAGGCTCATTTATCTCCCTAACAG-----------CAGTTA-TACTAA---TA 1404

* * **** ** ** * *** * * ** ****** ** * * **

P.pygmaeus-Ch2a ATAATGTTATTTTTATTATTTCCTGT-TCATAAGGCTGAGATTCTATTTTTCCACACTAT 1552

P.pygmaeus-cox1 ATTTTCATAATTTGAGAAGCCTTTGCCTCAAAACGAAAAGTCCCAATAATTGAACAACCT 1464

** * ** *** * * ** *** ** * ** * ** ** *** *

P.pygmaeus-Ch2a TGCCATAGGGAGCCATCAGGAGCATGTAAAAAAAAAAAAAACAGCTTATTCATTTGCGGA 1612

P.pygmaeus-cox1 T-CCACAAGC--CTAGAGTGGTTATACGGATGCCCCCCACCCTACC-ATACGTTTGAAGA 1520

* *** * * * * * ** * * * * ** * **** **

P.pygmaeus-Ch2a ATTTGCCTTTTGGTTTTTTA 1632

P.pygmaeus-cox1 ACCCGTCTATATAAAACCCG 1540

* ** *

**D1 (Macaque)**

M.mulatta-Ch1 IGSPPPTGSKKVVLRLRSVSSMVMPAARTGRERSRRIAVIKVDQTNKGVW

M.mulatta-Ch6(b) IGSPPPTGSKKVVLRLRSVSSMVMPAARTGRERSRRIAVIKIDQTNKGVW

M.mulatta-Ch6(a) IGSPPPTGSKKVVLRLRSVSSMVMPAARTGRERSRRIAVIKIDQTNKGVW

M.mulatta-Ch2 IGSPPPTGSKKVVLRLRSVSSMVMPAARTGKERSRRIAVIKIDQTNKGVW

M.mulatta-mt.st IGSPPPTGSKKVVLRLRSVSSMVMPAARTGRERSRRIAVIKIDQTNKGVW

M.mulatta-mt.mt MGSPPPTGSKKVVL*LRSVSSMVMPAA*TG*E*S**IAVIKIDQTNKGVW

:************* ************ ** * * *****:********

M.mulatta-Ch1 YWDIAGGFMLIIVVIKLMAPKIEEIPARWREKMTKSTEAPGWEKFPARGGY

M.mulatta-Ch6(b) YWDTAGGFMLMMVVMKLMAPKIEEIPARWREKMTKSTEAPGWEKFPARGGY

M.mulatta-Ch6(a) YWDTAGGFMLMMVVMKLMAPKIEEIPARWREKMTKSTEAPGWEKFPARGGY

M.mulatta-Ch2 YWDTAGGFMLMMVVMKLMAPKIEEIPARWREKMTKSTEAPGWEKFPARGGY

M.mulatta-mt.st YWDTAGGFMLIMVVMKLMAPKIEEIPAR*REKMTKSTEAPGWEKFPARGGY

M.mulatta-mt.mt YWDTAGGFMLMMVVMKLMAPKMEEMPA*W*EKMTKSTEAPGWEKFPA*GGY

*** ******::**:******:**:** * ***************** ***

**D2 (Macaque)**

M.mulatta-cox1 ATGCTCATTAATCGCTGACTCTTTTCAACAAATCACAAAGACATTGGAACCCTGTATT-T 59

M.mulatta-Ch1 ------------------------------------------------------------

M.mulatta-Ch6(b) ATACTTCTTCTAAAAGTTTACTTATTGTTTATCTGAAATTCAAATGTAATTTAGTATC-T 59

M.mulatta-Ch6(a) ----GGCCTCCCAAAGTGCTGGGATTACAAGCGTGAGCCACCACGCCCGGCCAAAGTAGC 56

M.mulatta-Ch2 ATGCTCATCAATCGCTGACTCTTTTCAACAAATCACAAAGACATTGGAACCCTGTATT-T 59

M.mulatta-cox1 ACTA--TTTGGTGCATGAGCTGGAATCATAGGCACTGCCCT----AAGCCTCCTCATTCG 113

M.mulatta-Ch1 ------------------------------------------------------------

M.mulatta-Ch6(b) AGCAACCCTGCATCCTGATATTCAACTTTCATACTCCTCTG----GCCATGACTGGCTTG 115

M.mulatta-Ch6(a) AGTTCTTTAAAGTTATAAGCCTGAGCGTGTGTATACCTATGTAACAAACCTGCACATCTG 116

M.mulatta-Ch2 ACTA--TTTGGTGCATGAGCTGGAATCATAGGCACTGCCCT----AAGCCTCCTCATTCG 113

M.mulatta-cox1 AGCTGAACTAGGCCAACCCGGCAACCTACTAGGCAACGACCACATCTATAACGTTATTGT 173

M.mulatta-Ch1 ------------------------------------------------------------

M.mulatta-Ch6(b) GGCAGGAGAGATCTTTGCCTGGATAGATGTTGGGAGGAGCCTGGTGAATGTATTCCTTCC 175

M.mulatta-Ch6(a) CACATATATCCCAGAACCCGGCAACCTACTGGGCAACGACCACATCTATAACGTTATTGT 176

M.mulatta-Ch2 AGCTGAACTAGGCCAACCCGGCAACCTACTAGGCAACGACCACATCTATAACGTTATTGT 173

M.mulatta-cox1 AACGGCCCAT--GCATTTGTCATAATTTTCTTTACAGTCATACCCATTATAATTGGAGGG 231

M.mulatta-Ch1 ------------------------------------------------------------

M.mulatta-Ch6(b) CAGGATTGGTCTGGAGCCTTTGTTGGGAGAATGGTGCCCTTTCTTATGGAAAGGGAGGGG 235

M.mulatta-Ch6(a) AACGGCCCAT--GCATTTGTTATAATTTTCTTTACAGTTATACCCATTATAATTGGGGGG 234

M.mulatta-Ch2 AACGGCCCAT--GCATTTGTTATAATTTTCTTTACAGTTATACCCATTATAATTGGAGGG 231

M.mulatta-cox1 TTCGGGAACTGACTAGTACCCCTAATAATCGGCGCCCCCGACATAGCATTCCCCCGTCTA 291

M.mulatta-Ch1 ------------------------------------------------TTTCCCCGTCTA 12

M.mulatta-Ch6(b) TTCGGGAACTGACTAGTGCCCCTAATAATCGGCGCTCCCGACATAGCATTTCCCCGTCTA 295

M.mulatta-Ch6(a) TTCGGGAACTGACTAGTGCCCCTAATAATCGGCGCCCCCGACATAGCATTTCCCCGTCTA 294

M.mulatta-Ch2 TTCGGGAACTGACTAGTGCCCCTAATAATCGGCGCTCCCGACATAGCATTTCCCTGTCTA 291

** *** *****

M.mulatta-cox1 AACAATATGAGCTTCTGACTCCTCCCCCCTTCTTTCCTGCTACTAATAGCATCCGCCGTG 351

M.mulatta-Ch1 AACAATATAAGCTTCTGACTCCTCCCTCCCTCTTTCCTGCTACTAATAGCATCAGCCGTA 72

M.mulatta-Ch6(b) AATAACATGAGCTTCTGACTCCTCCCCCCTTCTTTCCTGCTACTAATAGCATCCGCCGTG 355

M.mulatta-Ch6(a) AATAATATGAGCTTCTGACTCCTCCCCCCTTCTTTCCTGCTACTAATAGCATCCGCCGTG 354

M.mulatta-Ch2 AACAACATGAGCTTCTGACTCCTCCCCCCTTCTTTCCTGCTACTAATAGCATCCGCCGTG 351

** ** ** ***************** ** *********************** *****

M.mulatta-cox1 GTAGAAGCTGGCGCCGGAACAGGCTGAACAGTATACCCCCCTCTAGCAGGAAACTTCTCC 411

M.mulatta-Ch1 GTAGAAGCTGGCGCCGGAACAGGCTGAACAGT**ATATCCCCCTCTAGCAGGAAACTTCTCC** 132

M.mulatta-Ch6(b) GTAGAAGCTGGCGCCGGAACAGGCTGAACAGT**ATATCCCCCCCTGGCAGGAAACTTCTCC** 415

M.mulatta-Ch6(a) GTAGAAGCTGGCGCCGGAACAGGATGGACAGT**ATATCCCCCCCTGGCAGGAAACTTCTCC** 414

M.mulatta-Ch2 GTAGAAGCTGGCGCCGGAACAGGCTGAACAGT**ATATCCCCCCCTGGCAGGAAACTTCTCC** 411

*********************** ** ******** ***** ** ***************

M.mulatta-cox1 CACCCAGGAGCTTCTGTAGATTTAGTCATCTTCTCTCTTCACCTAGCAGGTATTTCCTCT 471

M.mulatta-Ch1 **CACCCGGGAGCTTCTGTAGATTTAGTCATCTTCTCCCTCCACCTAGCAGGTATTTCCTCT** 192

M.mulatta-Ch6(b) **CACCCAGGAGCTTCTGTAGACTTAGTCATCTTCTCTCTCCACCTAGCAGGTATTTCCTCT** 475

M.mulatta-Ch6(a) **CACCCAGGAGCTTCTGTAGACTTAGTCATCTTCTCTCTCCACCTAGCAGGTATTTCCTCT** 474

M.mulatta-Ch2 **CACCCAGGAGCTTCTGTAGACTTAGTCATCTTCTCTCTCCACCTAGCAGGTATTTCCTCT** 471

***** ************** ************** ** *********************

M.mulatta-cox1 ATCTTAGGAGCCATCAACTTCATTACCACCATTATCAACATAAAACCCCCCGCAGTATCC 531

M.mulatta-Ch1 **ATCTTAGGAGCCATCAACTTTATTACCACTATTATCAACATAAAACCTCCCGCAATATCC** 252

M.mulatta-Ch6(b) **ATCTTAGGAGCCATCAACTTCATTACCACCATCATCAACATAAAACCCCCCGCAGTATCC** 535

M.mulatta-Ch6(a) **ATCTTAGGAGCCATCAACTTCATTACCACCATCATCAACATAAAACCCCCCGCAGTATCC** 534

M.mulatta-Ch2 **ATCTTAGGGGCCATCAACTTCATTACCACCATCATCAACATAAAACCCCCCGCAGTATCC** 531

******** *********** ******** ** ************** ****** *****

M.mulatta-cox1 CAATACCAAACCCCTTTATTTGTCTGATCAATCTTAATCACAGCAATCCTTCTACTCCTC 591

M.mulatta-Ch1 **CAATACCAAACCCCTTTATTTGTCTGATCAACCTTAATCACAGCAATCCTTCTACTCCTC** 312

M.mulatta-Ch6(b) **CAATACCAAACCCCTTTATTTGTCTGATCAATCTTAATCACAGCAATCCTTCTACTCCTC** 595

M.mulatta-Ch6(a) **CAATACCAAACCCCTTTATTTGTCTGATCAATCTTAATCACAGCAATCCTTCTACTCCTC** 594

M.mulatta-Ch2 **CAATACCAAACCCCTTTATTTGTCTGATCAATCTTAATCACAGCAATCCTTCTACTCCTC** 591

******************************* ****************************

M.mulatta-cox1 TCTCTACCAGTTCTAGCCGCCGGCATTACCATGCTACTAACAGATCGCAACCTCAATACT 651

M.mulatta-Ch1 **TCTCTACCAGTCCTAGCCGCTGGCATTACCATGCTACTAACAGATCGCAACCTCAATACT** 372

M.mulatta-Ch6(b) **TCTCTACCAGTCCTAGCCGCTGGCATTACCATGCTACTAACAGATCGCAACCTCAATACT** 655

M.mulatta-Ch6(a) **TCTCTACCAGTCCTAGCCGCTGGCATTACCATGCTACTAACAGATCGCAACCTCAATACT** 654

M.mulatta-Ch2 **TCTTTACCAGTCCTAGCCGCTGGCATTACCATGCTACTAACAGATCGCAACCTCAATACT** 651

*** ******* ******** ***************************************

M.mulatta-cox1 ACTTTCTTTGACCCTGTTGGAGGAGGAGACCCTATCCTATATCAACACCTATTCTGATTC 711

M.mulatta-Ch1 **ACTTTCTTTGATCCTGTTGGAGGAGGAGATCCTAT**CCTATATGAACACCTGTTTTGATTC 432

M.mulatta-Ch6(b) **ACTTTCTTTGACCCTGTTGGAGGGGGAGACCCTAT**CCTATATCAACACCTATTTTGATTC 715

M.mulatta-Ch6(a) **ACTTTCTTTGACCCTGTTGGAGGAGGAGATCCTAT**CCTATATCAACACTTATTTTGATTC 714

M.mulatta-Ch2 **ACTTTCTTTGACCCTGTTGGAGGAGGAGATCCTAT**CCTATATCAACACCTATTTTGATTC 711

*********** *********** ***** ************ ***** * ** ******

M.mulatta-cox1 TTTGGTCACCCGGAAGTCTACATCCTTATTCTCCCCGGCTTCGGAATAGTCTCCCACATT 771

M.mulatta-Ch1 TTTGGCCACCCCGAAGTCTACATCCTTATTCTTCCCGGCTTTGGAATAGTCTCTCACATT 492

M.mulatta-Ch6(b) TTTGGTCACCCCGAAGTCTACATCCTTATTCTTCCCGGCTTCGGAATGATCTCTCACATT 775

M.mulatta-Ch6(a) TTTGGTCACCCCGAAGTCTACATCCTTATTCTTCCCGGCTTCGGAATGATCTCTCACATT 774

M.mulatta-Ch2 TTTGGTCACCCGGAAGTCTACATCCTTATTCTNNNNNNNNNNNNNNNNNNNNNNNNNNNN 771

***** ***** ********************

M.mulatta-cox1 GTAACCTACTACTCTGGGAAAAAAGAACCATTTGGGTACATGGGTATAGTTTGAGCCATA 831

M.mulatta-Ch1 GTAACTTACTACTCTGGAAAAAAAGAACCATTTGGGTATATGGGTATAGTTTGAGCCATG 552

M.mulatta-Ch6(b) GTAGCTTACTACTCTGGAAAAAAAGAACCATTTGGGTATATGGGCATAGTTTGGGCCATA 835

M.mulatta-Ch6(a) GTAGCTTACTACTCTGGAAAAAAAGAACCATTTGGGTATATGGGCATAGTTTGGGCCATA 834

M.mulatta-Ch2 NNNNNNNNNNNNNNNNNNNNNNNNNNNNNNNNNNNNNNNNNNNNNNNNNNNNNNNNNNNN 831

M.mulatta-cox1 ATATCAATTGGGTTTTTAGGTTTTATTGTATGAGCCCACCACATGTTTACAGTTGGCATA 891

M.mulatta-Ch1 ATATCAATTGGGTTTTTAGGTTTTATTGTATGAGCCCACCACATGTTTACAGTTGGCATA 612

M.mulatta-Ch6(b) ATATCAATTGGGTTTTTAGGTTTTATTGTATGAGCCCACCACATGTTNNNNNNNNNNNNN 895

M.mulatta-Ch6(a) ATATCAATTGGGTTTTTAGGTTTTATTGTATGAGCCCNNNNNNNNNNNNNNNNNNNNCAT 894

M.mulatta-Ch2 NNNNNNNNNNNNNNNNNNNNNNNNNNNNNNNNNNNNNNNNNNNNNNNNNNNNNNNNNNNN 891

M.mulatta-cox1 GACGTAGATACACGAGCC--------TATTTCACCTCCGCCACTATAATCATT---GCAA 940

M.mulatta-Ch1 GACGTAGATACACGAGCC--------TATTTCACTTCCGCCACTATAATCATT---GCAA 661

M.mulatta-Ch6(b) NNNNNNNATAGGGATCTCCTTCCCTATGTTTGGAAAACACTGGTCTAAGCATTTCAACAA 955

M.mulatta-Ch6(a) GCCTGCCACGAACTGTGGGTGAGAAATGTGGTCATGCTTCTAAACCAATTATT------C 948

M.mulatta-Ch2 NNNNNNNNNNNNNNNNNNN-------NNNNNNNNNNNNNNNNNNNNNNNNNNNNNNNNNN 944

M.mulatta-cox1 TCCCCACCGGTGTGAA--AGTTTTTAGCTGGCTTGCTACACTTCATGGGG------GCAA 992

M.mulatta-Ch1 TCCCCACCGGTGTGAA--AGTTTTTAGCTGGCTTGCTACACTTCATGGAG------GCAA 713

M.mulatta-Ch6(b) TAGCCTTGAATATAAATGAATGTCTACTTAGCAATCATCTTTTCCAGATGATTCTTGTAT 1015

M.mulatta-Ch6(a) TAATCAAGGCTTTGATTCAGTGTGCTCAAAACAGAGAAGACTGGCCAGGGAATTTCCCAT 1008

M.mulatta-Ch2 NNNNNNNNNNNNNNNNNNNNNNNNNNNNNNNNNNNNNNNNNNNNNNNNNNNNNNNNNNNN 1004

M.mulatta-cox1 CATCAAATGGT----CCCCAGCAATACTTTGAGCCCTAGGCTTTATCTTCC-TATTTACT 1047

M.mulatta-Ch1 CATTAAATGAT----CGCCAGCAATACTTTGAGCCCTAGGCTTTATCTTCC-TATTTACT 768

M.mulatta-Ch6(b) CTTAAAATAATGTTACTTCAAGAAGGTGCCTAATTGTAAGACATCCTTACAGTAATAGTA 1075

M.mulatta-Ch6(a) TTAAGAAAGAAGGTTGATCAGTATGAATCTGATGCTTA-----TCTTTAAGGATAAGATT 1063

M.mulatta-Ch2 NNNNNNNNNTTTCCTTCTTGGGAAGGCCTTCAAGTAT------TCAAAGGGACTTGGGTG 1058

* * * *

M.mulatta-cox1 GTAGGGGGTCTAACTGGCATCATCTTAGCAAACT-CATCCCTAGATATCGTACTACACGA 1106

M.mulatta-Ch1 GTAGGAGGTCTAACCGGCATCATCTTGGCAAACT-CATCCCTAGATATCGTACTACACGA 827

M.mulatta-Ch6(b) GTTGGGGTTATGATGAAATTTGATAATGTGAGAT-TCTCTTAGAAAATAAAATTA-GTAA 1133

M.mulatta-Ch6(a) ATATGTCCCACATAATGACACAATAAAATATATTCTACTCTAAGAAACTTAAATATATGA 1123

M.mulatta-Ch2 CAGTGATTTAAGTCTTTGGTTACTGCAGCTGTATCTGCATTAAGGAACATTCCAAGACTA 1118

* * * * *

M.mulatta-cox1 TACATA-CTACGTTGTCGCCCAC----TTTCATTATGTTCTATCA-ATAGGAGCTGTATT 1160

M.mulatta-Ch1 CACATA-CTACGTTGTCGCCCAC----TTCCATTATGTTCTATCA-ATAGGAGCTGTATT 881

M.mulatta-Ch6(b) TAAGTAACTACAACTGAGTTTATAA--TCTTAATAAATTAAAGAA-GCTTAATTTAAAAT 1190

M.mulatta-Ch6(a) ATGAAATTTCTGAAAGAGTTTTTTAAATTCTATTCAACCATCTTGTATCTTTCCTCTTTT 1183

M.mulatta-Ch2 ATAATGCTTTGACTCTTGCAGACTCATAGAGGTACTATGGTGGTG-GTCTTGGTTGAAGC 1177

* * *

M.mulatta-cox1 -CGCCATTATGGGGGGCTTTATACACTGATTCCCTTTATTCTCAGGC------TACA-CA 1212

M.mulatta-Ch1 -TGCCATTATAGGAGGCTTCATACATTGATTTCCTTTATTCTCAGGC------TATA-CA 933

M.mulatta-Ch6(b) GTGTAACTAAAGAGAAACTAAGTCACAAAAACTCCTTGATGACAGGAGTAACATATAACA 1250

M.mulatta-Ch6(a) CCCTCCGTTTTTCAACCCTTGAAAATGGGAATGCCACAATATTTAATGT--TTAATTCTA 1241

M.mulatta-Ch2 CTGGGAGCATTCCCTAGATTACTAGGCAGATACTTTTATTCTTTTTCCAG--ACATT-CC 1234

* * *

M.mulatta-cox1 CTGAACCAA-----ACCTGTGCCAAAGCCCA----CTTCATCATTATATT-CATGGGCGT 1262

M.mulatta-Ch1 CTGAACCAG-----ACCTGTGCCAAAGCCCA----CTTCATCATTATATT-CGTGGGCGT 983

M.mulatta-Ch6(b) GGGAGACAGTAGTAAGTAGTGGCTAAGGGTAAAGGCTGTGGAATTACACTACCTGGGTCC 1310

M.mulatta-Ch6(a) CAAAAATAACTGCAGGGAATAA-TATGTGTA-----TTAGTCAGTGTTCTCCAGGGAAAC 1295

M.mulatta-Ch2 CCCAATCAGATTGAGTCTCTCTCTGTGCTGAGCT-TTCTGGAGCTGGAGGAGCTGTGAAA 1293

* * * * * * * *

M.mulatta-cox1 AA-ATTTAAC-CTTCTTTCCACAACACTTCCTTGGCCTGTCCGGAATACCCCGACGCTAC 1320

M.mulatta-Ch1 AA-ATTTAAC-CTTCTTTCCACAACACTTCCTTGGCCTCTCCGGAATACCCCGACGCTAC 1041

M.mulatta-Ch6(b) AATATCTAACACTGATTATTGTGGTGACTTACTGAATTTTCCTCACCTACAAACTAAGTT 1370

M.mulatta-Ch6(a) AG-AATCAAT-----TTATTATGACACAT----GGACTATCCAGTAACGGAGGCTGAGAA 1345

M.mulatta-Ch2 CAAGCACTCCTCTGGCTACCACCACTAAA-ACTGCACTGGATGAGACCTGAAGCCATCAC 1352

* * *

M.mulatta-cox1 TCTGATTATCCCGATGCCTACACTACATGAAATACCCTATCATCTATAGG-CTCCTTCAT 1379

M.mulatta-Ch1 TCTGATTATCCCGATGCCTACACTACATGAAATATCCTATCATCTATGGG-CTCCTTCAT 1100

M.mulatta-Ch6(b) ACAACCTATTGTGTTTTTTTAATTGATTGAGATTATTAACACTTTTTATA-ATGCCTGGT 1429

M.mulatta-Ch6(a) TTTCCAACAGCTGTTATCTGCAC-ACTGAAGAACCAGGAAAACTAGTGGT-GTAATCAGT 1403

M.mulatta-Ch2 AGCACTGGGTTTTGCCCAGGACCCAGGGTGACCACTGCCTGACTACTACATATGTTCACT 1412

* * *

M.mulatta-cox1 C-----TCACTA---GTAGCAGTAATTTTAATAATCTACATGAT---CTGAGAAGCCTTT 1428

M.mulatta-Ch1 C-----TCACTA---GTAGCAGTAATTTTAATAATCTACATGAT---CTGAGAAGCCTTT 1149

M.mulatta-Ch6(b) CCAGAGTAAATG---CTCAAAACACTTTACCTATTTTATATGCTAAATTTAAACTTAATC 1486

M.mulatta-Ch6(a) CTGTGTCCAAAGG-CCTGGGAGCCAGTGTTCTAAGCCCTGAAGT--ACAAAGACCCGAGA 1460

M.mulatta-Ch2 TATTTCCCAGGGGATCTACCATCAACTGCTGGTGAATCCATCCAC-GTTCAGTTCCTTTC 1471

* * * * *

M.mulatta-cox1 GCTTCAAAACGTAAAGTACTACTAATCGAAC--AACCCCTTACTAACCTAGAGTGA---- 1482

M.mulatta-Ch1 GCTTCAAAACGGAAAGTACTACTAATCGAAC--AACCCCTTACTAACCTAGAATGA---- 1203

M.mulatta-Ch6(b) TTTTTGAGACTTAATATATATCCATGCACATTGAACATATTTTAAATTCAGAAAGGAAAC 1546

M.mulatta-Ch6(a) ACCAGGAGCTGCAATGCCTGAGGATAGGAACAGATGGATGTCTCAGCACAAAAGAG---- 1516

M.mulatta-Ch2 CTTTAGGATGGCAAGTTCCCCCGGTCCTGAGTGGGTCCAGGTATGCCATCCAGGAG---C 1528

** * *

M.mulatta-cox1 CTAAATGGCTGCC------CCCCGCCTTATCACACATTCGAAGAACCACCCTACATTAAA 1536

M.mulatta-Ch1 CTAAATGGCTGCC------CCCCGCCTTATCACACATTCGAAGAACCAGCCTATATTAAA 1257

M.mulatta-Ch6(b) CGATATGCCAGCAAAAACACAGCAAATATTCTTGTTTTCAAATAAAGACCATTTCTTTAT 1606

M.mulatta-Ch6(a) -AGAATTCATTCT----TCCTCCATCTTTTTGCTCAGTTTGG-GCCCACAACAAATTAAA 1570

M.mulatta-Ch2 CAGGGCCTGAAGTCAAGAGCCTTAGGAATCTACCTATTTAACTGTGATGCCCTATTTAAA 1588

* * ** *

M.mulatta-cox1 CTAGTC 1542

M.mulatta-Ch1 CTAAAT 1263

M.mulatta-Ch6(b) GAGAAA 1612

M.mulatta-Ch6(a) CAGTGC 1576

M.mulatta-Ch2 TGTGGC 1594

**E1 (horse)**

E.caballus-Ch27 IGSPTPAGSKKVVFRLRSVRSIVMPAAKTGRASRRSTAVIRTDQTNRGV*

E.caballus-mt.st IGSPPPAGSKKVVFRLRSVRSMVMPAARTGRARRRSTAVIRTDQTNRGV*

E.caballus-mt.mt IGSPPPAGSKKVVF*LRSV*SMVMPAA*TG*A***STAVM*TDQTN*GVW

****.********* **** *:***** ** * ****: ***** *

E.caballus-Ch27 YWDRAGGFILMIVVIKLMAPKIEDRPVRWREKIVKSMEAPECARFPARGGY

E.caballus-mt.st YWDRAGGFMLMIVVIKLMAPKIEDTPARWREKMVKSTEAPACARFPARGGY

E.caballus-mt.mt YWD*AGGFMLMIVVMKLMAPKIEDTPA*W*EKMVKSTEAPACA*FPA*GGY

*** ****:*****:********* *. * **:*** *** ** *** ***

**E2 (horse)**

E.caballus-Ch27 ATGTTCATTACTCGCTGCTTATTCTCAACCAACCACAAAGACATCGGCACTTTATATCTC 60

E.caballus-cox1 ATGTTCATCAACCGCTGACTATTTTCAACTAACCACAAAGACATCGGCACTCTGTACCTC 60

******** * ***** **** ***** ********************* * ** ***

E.caballus-Ch27 CTATTCGGCGTTTGAGCTGGAATAGTAGGAACCGCCCTAAGCCTCTTAATTTGTGCTGAA 120

E.caballus-cox1 CTATTCGGCGCTTGAGCTGGAATAGTAGGAACTGCCCTAAGCCTCCTAATCCGTGCTGAA 120

********** ********************* ************ **** ********

E.caballus-Ch27 TTAGGTCAACCTGGAACCCTGCTAGGAGATGATCAAATCTACAATGTTATTGTAACCGCC 180

E.caballus-cox1 TTAGGCCAACCTGGGACCTTACTAGGAGATGATCAGATCTACAATGTCATTGTAACCGCC 180

***** ******** *** * ************** *********** ************

E.caballus-Ch27 CATGCATTCGTAATAATTTTCTTCATAGTTATACCTATCATGATTGGAGGATTTGGAAAC 240

E.caballus-cox1 CATGCATTCGTAATAATTTTCTTTATGGTCATACCCATTATAATCGGAGGATTCGGAAAC 240

*********************** ** ** ***** ** ** ** ******** ******

E.caballus-Ch27 TGATTAGTCCCCCTAATAATTGGAACACCCGATATAGCTTTCCCTTGAATAAACAATATA 300

E.caballus-cox1 TGATTAGTCCCCCTGATAATTGGAGCACCTGATATAGCTTTCCCCCGAATAAACAACATA 300

************** ********* **** ************** ********** ***

E.caballus-Ch27 AGCTTCTGACTACTCCCCCCATCATTCCTGCTCCTTCTTGCTTCCTCAATAATTGAAGCA 360

E.caballus-cox1 AGCTTCTGACTACTTCCCCCATCATTCCTACTTCTTCTCGCTTCCTCAATAATTGAAGCA 360

************** ************** ** ***** *********************

E.caballus-Ch27 GGCGCTGGGACGGGTTGAACCGT**ATATCCTCCCCTGGCTGGAAATCTGGCGCATTCAGGA** 420

E.caballus-cox1 GGTGCCGGAACAGGCTGAACCGTATATCCTCCTCTAGCTGGAAATCTGGCGCATGCAGGA 420

** ** ** ** ** ***************** ** ****************** *****

E.caballus-Ch27 **GCCTCCATTGACTTAACTATTTTCTCTCTCCACCTGACTGGTCTATCTTCAATTTTAGGT** 480

E.caballus-cox1 GCCTCTGTTGACTTAACCATTTTCTCTCTCCACCTAGCTGGGGTGTCCTCAATTTTAGGT 480

***** ********** ***************** **** * ** ************

E.caballus-Ch27 **GCCATCAACTTTATCACTACAATCATCAATATAAAACCACCAGCCCTGTCCCAGTATCAA** 540

E.caballus-cox1 GCCATCAACTTTATTACCACAATCATTAACATAAAACCACCGGCTCTATCCCAATATCAA 540

************** ** ******** ** *********** ** ** ***** ******

E.caballus-Ch27 **ACTCCCCTATTCGTCTGATCCGTCCTTATCACAGCAGTACTCCTTCTACTAGCTCTCCCA** 600

E.caballus-cox1 ACCCCCCTATTCGTTTGATCTGTCCTTATTACGGCAGTACTCCTTCTCCTAGCCCTCCCG 600

** *********** ***** ******** ** ************** ***** *****

E.caballus-Ch27 **GTCTTAGCAGCAGGCATTACTATACTTCTCACAGACCGTAATCTAAATACTACTTTCTTC** 660

E.caballus-cox1 GTCCTAGCAGCAGGCATTACCATGCTTCTCACAGACCGTAACCTAAACACTACTTTCTTC 660

*** **************** ** ***************** ***** ************

E.caballus-Ch27 **GACCCCGCGGGGGTAGGAGATCC**AATCCTTTACCAACACCTATTCTGATTCTTCGGACAC 720

E.caballus-cox1 GACCCCGCAGGAGGAGGGGATCCAATCCTTTATCAACACCTATTCTGATTCTTCGGACAC 720

******** ** * *** ************** ***************************

E.caballus-Ch27 CCCGAAGTCTACATTCTCATCCTACCGGGCTTCGGGATGATCTCACACTTCGTCACATAC 780

E.caballus-cox1 CCCGAAGTCTATATTCTTATCCTACCAGGCTTCGGTATAATCTCACACATCGTCACATAC 780

*********** ***** ******** ******** ** ********* ***********

E.caballus-Ch27 TAGTCAGGTAAAAAAGAACCATTTGGCTACATGGGCATGGTATGAGCTATAATGTCCATC 840

E.caballus-cox1 TACTCAGGTAAAAAGGAACCTTTTGGCTACATGGGTATAGTGTGAGCTATAATATCCATT 840

** *********** ***** ************** ** ** *********** *****

E.caballus-Ch27 GGCTTCTTAGGCTTCATCGTATGAGCTCACCACATGTTCACAGTAGGAATGGACATTGAC 900

E.caballus-cox1 GGCTTTCTAGGCTTCATCGTATGGGCTCACCACATGTTTACAGTAGGGATAGACGTTGAC 900

***** **************** ************** ******** ** *** *****

E.caballus-Ch27 ACACGAGCATACTTCACATCAGCTACCGTGATAATCGCCATCCCCACTGGTGTAAAAGTA 960

E.caballus-cox1 ACACGAGCATACTTCACATCAGCTACCATAATCATCGCTATCCCTACCGGTGTAAAAGTA 960

*************************** * ** ***** ***** ** ************

E.caballus-Ch27 TTTAGCTGGCTGGCCACCCTTCATGGAGGAAATATTAAATGATCTCCAGCCATACTCTGA 1020

E.caballus-cox1 TTCAGCTGACTAGCCACCCTGCACGGAGGAAATATCAAATGATCTCCAGCTATACTCTGA 1020

** ***** ** ******** ** *********** ************** *********

E.caballus-Ch27 GCCCTAGGCTTCATCTTCTTATTTACAGTAAGGGGTTTAACAGGGATCGTCTTAGCCAAC 1080

E.caballus-cox1 GCTCTAGGCTTCATCTTCTTATTCACAGTAGGAGGTCTAACAGGAATCGTCCTAGCTAAC 1080

** ******************** ****** * *** ******* ****** **** ***

E.caballus-Ch27 TCCTCTCTAGATATTGTCCTCCATGATACTTATTATGTAGTAGCACATTTCCACTACATC 1140

E.caballus-cox1 TCATCCCTAGATATTGTTCTCCACGATACTTATTATGTAGTAGCACATTTCCATTATGTC 1140

** ** *********** ***** ***************************** ** **

E.caballus-Ch27 TTATCTATAGGAGCAGTCTTTGCTATTATAGGAGGGTTTGTTCACTGATTCCCCCTATTC 1200

E.caballus-cox1 CTGTCTATAGGAGCAGTCTTCGCCATTATGGGGGGATTTGTACACTGATTCCCTCTATTC 1200

* ***************** ** ***** ** ** ***** *********** ******

E.caballus-Ch27 TCAGGATATACACTCAATCAAACCTGAGCAAAAGTCCACTTTACAATCATATTCGTAGGA 1260

E.caballus-cox1 TCAGGATACACACTCAACCAAACCTGAGCAAAAATCCACTTTACAATTATATTCGTAGGG 1260

******** ******** *************** ************* ***********

E.caballus-Ch27 GTCAACATAACCTTCTTCCCACAACATTTTCTTGGCCTCTCAGGAATGCCACGACGCTAT 1320

E.caballus-cox1 GTAAATATAACCTTCTTCCCACAACATTTCCTTGGCCTCTCAGGAATGCCACGACGCTAT 1320

** ** *********************** ******************************

E.caballus-Ch27 TCCCATTACCCAGACGCATATACACCATGAAATACAATCTCATCCATAGGATCTTTCATC 1380

E.caballus-cox1 TCTGATTACCCAGACGCATATACAACATGAAATACCATCTCATCCATAGGATCTTTTATC 1380

** ******************** ********** ******************** ***

E.caballus-Ch27 TCACTTACAGCAGTAATACTAATAATTTTCATGATCTGAGAAGCATTCGCATCCAAACAA 1440

E.caballus-cox1 TCACTTACAGCAGTGATACTAATAATTTTCATAATTTGAGAAGCATTCGCATCCAAACGA 1440

************** ***************** ** ********************** *

E.caballus-Ch27 GAAGTATCTACAGTAGAACTAACTTCAACCAATCTCGAATGACTACACGGATGCCCTCCG 1500

E.caballus-cox1 GAAGTGTCTACAGTAGAATTAACCTCAACTAATCTGGAATGACTACACGGATGCCCCCCA 1500

***** ************ **** ***** ***** ******************** **

E.caballus-Ch27 CCATATCATACATTTGAAGAACATGCCTATGTGAACCCAAAATAA 1545

E.caballus-cox1 CCATACCACACATTTGAAGAACCCACCTACGTAAACCTAAAATAA 1545

***** ** ************* **** ** **** *******

**F1 (dog)**

C.familiaris-Ch16 MGSPPPAGSKNVVFRFWSVRSIVIPAANTGNDNSNRTAVISMDHTNKGVW

C.familiaris-mt.st IGSPPPAGSKNVVLRFRSVKSIVIPAASTGRDSKSRTAVISTDHTNRGV*

C.familiaris-mt.mt MGSPPPAGSKNVVL*FRSVKSIVIPAASTG*DSKS*TAVISTDHTN*GVW

:************: * **:*******.** *... ***** **** **

C.familiaris-Ch16 YWDMAGGFILIIVVIKLIAPRMEEAPAKCKEKIVRSTDAPAWARLLASGGY

C.familiaris-mt.st YWDIAGGFMLIIVVMKLIAPKIEETPAKCKEKIVRSTDAPAWARLPASGGY

C.familiaris-mt.mt YWDIAGGFMLMMVVMKLIAPKMEETPAKCKEKIV*STDAPAWA*LPASGGY

***:****:*::**:*****::**:********* ******** * *****

**F2 (dog)**

C.familiaris-Ch16 TCTGCCCATGCATTCGTAATAATTTTCTTTATAGTTATACCTAT-ATAATTGGGGCTTTC 59

C.familiaris-cox1 ACCGCCCATGCTTTCGTAATAATCTTCTTCATAGTCATGCCCATCATAATTGGGGGCTTT 60

* ******** *********** ***** ***** ** ** ** ********** **

C.familiaris-Ch16 GGAAACTGACTAGTTCCGCTAATAATTGGCACCCCAGACATGGCATTCCCTCGAATAAAT 119

C.familiaris-cox1 GGAAACTGACTAGTGCCGTTAATAATTGGTGCTCCGGACATGGCATTCCCCCGAATAAAT 120

************** *** ********** * ** ************** *********

C.familiaris-Ch16 AACATAAGTTTCTGGTTGCTCCCCCCATCTTTCCTCCTCCAACTAGCATCTTCTATGGTA 179

C.familiaris-cox1 AACATGAGCTTCTGACTCCTTCCTCCATCCTTTCTTCTACTATTAGCATCTTCTATGGTA 180

***** ** ***** * ** ** ***** ** ** ** * * *****************

C.familiaris-Ch16 GAAGCAGGCGCAGGAACTGGGTGAACTGT**ATACCCCCCACTAGCCAGCAACCTGGCCCAC** 239

C.familiaris-cox1 GAAGCAGGTGCAGGAACGGGATGAACCGTATACCCCCCACTGGCTGGCAATCTGGCCCAT 240

******** ******** ** ***** ************** ** **** ********

C.familiaris-Ch16 **GCAGGGGCATCAGTAGACCTAACAATTTTTTCCTTGCATTTAGCAGGGGCCTCCTCCATT** 299

C.familiaris-cox1 GCAGGAGCATCCGTTGACCTTACAATTTTCTCCTTACACTTAGCCGGAGTCTCTTCTATT 300

***** ***** ** ***** ******** ***** ** ***** ** * *** ** ***

C.familiaris-Ch16 **CTTGGGGCAATTAATTTTATCACTACTATTATTAATATAAAACCCCCAGCCATGTCCCAA** 359

C.familiaris-cox1 TTAGGGGCAATTAATTTCATCACTACTATTATCAACATAAAACCCCCTGCAATATCCCAG 360

* ************** ************** ** *********** ** ** *****

C.familiaris-Ch16 **TACCAAACTCCCTTGTTTGTATGGTCCATACTAATTACAGCAGTCCTATTGCTATTATCA** 419

C.familiaris-cox1 TATCAAACTCCCCTGTTTGTATGATCAGTACTAATTACAGCAGTTCTACTCTTACTATCC 420

** ********* ********** ** **************** *** * ** ****

C.familiaris-Ch16 **TTGCCTGTATTAGCTGCTGGAATTACAATACTTCTGACAGACCAAAATCTAAACACAACA** 479

C.familiaris-cox1 CTGCCTGTACTGGCTGCTGGAATTACAATACTTTTAACAGACCGGAATCTTAATACAACA 480

******** * ********************* * ******* ***** ** ******

C.familiaris-Ch16 **TTTTTCGATCCTGCTGGAGGGGGAGACCCCAT**TCTATACCAACACTTATTCTGATTTTTC 539

C.familiaris-cox1 TTTTTTGATCCCGCTGGAGGAGGAGACCCTATCCTATATCAACACCTATTCTGATTCTTC 540

***** ***** ******** ******** ** ***** ****** ********** ***

C.familiaris-Ch16 AGGCACCCTGAAGTTTATATTCTAATTTTACCTGGATGTGGAATAATTTCCCATATCGTT 599

C.familiaris-cox1 GGACATCCTGAAGTTTACATTCTTATCCTGCCCGGATTCGGAATAATTTCTCACATTGTC 600

* ** *********** ***** ** * ** **** *********** ** ** **

C.familiaris-Ch16 ATGTATTACTCAGGGAAAAAAAGAGCCCCTTGGTTACATGGGTATGGTCTGAGCAATGAT 659

C.familiaris-cox1 ACTTACTACTCAGGGAAAAAA-GAGCCTTTCGGTTATATAGGAATAGTATGAGCAATAAT 659

* ** *************** ***** * ***** ** ** ** ** ******** **

C.familiaris-Ch16 ATCCATTGGGTTTCTAGGCTTTATTGTATGAACACATCATATATTTACTGTGGGAAGAGA 719

C.familiaris-cox1 ATCTATTGGGTTTTTAGGCTTTATCGTATGAGCTCACCATATGTTTACCGTAGGAATAGA 719

*** ********* ********** ****** * ** ***** ***** ** **** ***

C.familiaris-Ch16 TGTAGACACACGAGCATATTTTACATCTGCTACTATAATTATTGCCATCCCAACAGGGGT 779

C.familiaris-cox1 TGTAGACACACGAGCGTACTTTACGTCCGCCACTATAATTATCGCTATTCCAACGGGAGT 779

*************** ** ***** ** ** *********** ** ** ***** ** **

C.familiaris-Ch16 TAAAGTATTTAGCTGATTAGCAACACTTCATGGAGGAAATATCAAATGATCTCCTGCCAT 839

C.familiaris-cox1 AAAAGTATTTAGTTGACTGGCAACACTTCATGGAGGCAATATTAAATGATCTCCAGCTAT 839

*********** *** * ***************** ***** *********** ** **

C.familiaris-Ch16 ACTATGGGCTCTAGGATTTATCTTCCTATTCACAGTAGGTGGGTTAACAGGTAGTGTTCT 899

C.familiaris-cox1 GCTATGAGCTTTAGGGTTTATTTTCTTATTTACAGTAGGCGGGTTAACAGGTATTGTCCT 899

***** *** **** ***** *** **** ******** ************* *** **

C.familiaris-Ch16 AGCCAACTCATCCCTAGACATTGTACTTCATGACACATATTACGTAGTAGCCCACTTCCA 959

C.familiaris-cox1 AGCTAATTCGTCCTTAGACATCGTTCTTCATGATACATATTATGTTGTGGCTCATTTTCA 959

*** ** ** *** ******* ** ******** ******** ** ** ** ** ** **

C.familiaris-Ch16 CTAAGTACTCTCAAAAGGAGCAGTGTTTGCTATTATGGGCGGATTTGCTCACTGATTCCC 1019

C.familiaris-cox1 CTATGTGCTTTCAATAGGAGCAGTTTTTGCCATTATGGGAGGATTTGCCCACTGATTCCC 1019

*** ** ** **** ********* ***** ******** ******** ***********

C.familiaris-Ch16 CCTATTTTCAGGATATACTCTTAATGACACTTGAGCAAAAATCCACTTTACATACACCAG 1079

C.familiaris-cox1 TTTATTCTCAGGTTATACTCTTAACGATACTTGAGCAAAGATTCACTTTACAATTAT--G 1079

**** ***** *********** ** *********** ** ********* * *

C.familiaris-Ch16 TTAACAATGACCAACCAGTGACAATTACTAATCAA-GTCTCATAACTATATAACGCCGCA 1138

C.familiaris-cox1 TTTGTGGGAGTAAAT--ATAACTTTCTTCCCTCAACATTTCCTAGGTTTAT----CTGGA 1133

** ** * ** * **** * ** ** * *** * * *

C.familiaris-Ch16 ATCCCCCCCATAGCTTCCTCACTAAAGAACCCTGAATACCCGGTATCATAAATAACTCAA 1198

C.familiaris-cox1 ATACCTCGT-CGATACTCTGACTACCCAG--ATGCATATACTACCTGAAATACCGTCTCC 1190

** ** * ** **** * ** *** * * * * *

C.familiaris-Ch16 TCCCCCATCCCATTAAATTTCAACACCACCTCAACCCCATCATCCTTTAAGATATAGCAA 1258

C.familiaris-cox1 TCTATAGGATCGTTTATCTCGCTTACAGCGGTGATGCTTATAATTTTTATGATCTGGGAA 1250

** * ** * * ** * * * * **** *** * * **

C.familiaris-Ch16 GCAGTTAATAACTCAGACAGTAAGCCAGTAATGAAAGCGGCCAATACGGCCTTATTAGAA 1318

C.familiaris-cox1 GCCTTTGC--ATCCAAACGAGAAGTTGCTA-TAGTAGAACTTACTACAAC--TAACATTG 1305

** ** * ** ** *** ** * ** * *** * ** *

C.familiaris-Ch16 ACTCAGACCTCAGGGTACTGTTCAGTAGCCATAGCAGTCGTATAACCAAAAACTACAAGC 1378

C.familiaris-cox1 AGT--GACTACATGG--ATGTCCC-CCTCCATACCACACGTTCGA--AGAACCTACATAT 1358

* * *** ** ** *** * ***** ** *** * * ** *****

C.familiaris-Ch16 ATACCTCCCAAATAA 1393

C.familiaris-cox1 GTGATCCAAAAATAA 1371

* * ******

**G1 (cow)**

B.taurus-Ch10 IGSPPPAGSKKVVFRFRSVSSIVMPAASTGSERSSSTAVIITDQMTRGVWYCDIAGGFML 60

B.taurus-mt.st IGSPPPAGSKKVVFRFRSVNSIVMPAANTGSESSSSTAVIITDHTNRGVWYCDIAGGFML 60

B.taurus-mt.mt MGSPPPAGSKKVVF*FRSVNSIVMPAANTGSESSSSTAVIITDHTN*GVWYCDIAGGFML 58

:************* ****.*******.**** **********: . *************

B.taurus-Ch10 IIVVIKLIAPKIEETPAKCKEKMVRSTEAPAWARLPAKGGY 101

B.taurus-mt.st IIVVMKLMAPKIEETPAKCKEKMVRSTEAPAWARLPAKGGY 101

B.taurus-mt.mt MIVVMKLMAPKIEETPAKCKEKMV*STEAPAWA*LPAKGGY 97

:***:**:**************** ******** *******

**G2 (cow)**

B.taurus.Ch10 NNNNNNNTCAACCGCTGATTATTCTCAACTAATCATAAAGATATCGGTACCCTATACTTA 60

B.taurus-cox1 ATGTTCATTAACCGCTGACTATTCTCAACCAGCCATAAAGATATTGGTACCCTTTATCTA 60

* ********* ********** * *********** ******** ** **

B.taurus.Ch10 CTATTTGGTGCCTGGGCCGGCATAGTAGGAACAGCCCTAAGC-TGCTAATTCGCGCTGAA 119

B.taurus-cox1 CTATTTGATGCTTGGGCCGGTATAGTAGGAACAGCTCTAAGCCTTCTAATTCGCGCTGAA 120

******* *** ******** ************** ****** * ***************

B.taurus.Ch10 TTGGGTCAACCTGGAACCCTGCTCGGAGATGACCAAATCTACAATGTAGTTGTAACTGCA 179

B.taurus-cox1 TTAGGCCAACCCGGAACTCTGCTCGGAGACGACCAAATCTACAACGCAGTTGTAACCGCA 180

** ** ***** ***** *********** ************** * ********* ***

B.taurus.Ch10 CACGCATTTGTAATAATGTTCTTTACAGTAATGCCAATTATAATTGGAGGGTTCAGTAAT 239

B.taurus-cox1 CACGCATTTGTAATAATCTTCTTCATAGTAATACCAATCATAATTGGAGGATTCGGTAAC 240

***************** ***** * ****** ***** *********** *** ****

B.taurus.Ch10 TGACTCGTCCCTCTAATAATTGGCACCCCCGATATAGCATTCCCCTGGATAAATAATAAA 299

B.taurus-cox1 TGACTTGTTCCCCTAATAATTGGTGCTCCCGATATAGCATTTCCCCGAATAAATAATATA 300

***** ** ** *********** * ************** *** * ********** *

B.taurus.Ch10 AGCTTCTGACTCCTTCCCCTCTCCTTCCTACTACTTCAAGCATCATCCATAGTTGAGGCT 359

B.taurus-cox1 AGCTTCTGACTCCTCCCTCCCTCATTCCTACTACTCCTCGCATCCTCTATAGTTGAAGCT 360

************** ** * *** *********** * ***** ** ******** ***

B.taurus.Ch10 GGGGCAGGAACAGGTTGAACCAT**GTATCCCCCTTTAGCAGGTAACCTGGCCCACGCAGGA** 419

B.taurus-cox1 GGGGCAGGAACAGGCTGAACCGTGTACCCTCCCTTAGCAGGCAACCTAGCCCATGCAGGA 420

************** ****** **** ** ** ******** ***** ***** ******

B.taurus.Ch10 **GCCTCAGTAGACCTAACCATTTTCTCTTTACACTTGGCAGGTGTCTCCTCAATTTTAGGG** 479

B.taurus-cox1 GCTTCAGTAGATCTAACCATTTTCTCTTTACACTTAGCAGGAGTTTCCTCAATTTTAGGA 480

** ******** *********************** ***** ** **************

B.taurus.Ch10 **GCTATTAACTTTATTACAACAATTATTAACATAAAACCCCCTGCAATGTCACAATACCAA** 539

B.taurus-cox1 GCCATCAACTTCATTACAACAATTATCAACATAAAGCCCCCCGCAATGTCACAATACCAA 540

** ** ***** ************** ******** ***** ******************

B.taurus.Ch10 **ACCCCTCTAGTCATTTGATCTGTAATAATCACCGCCGTACTACTACTCCTCTCACTTCCT** 599

B.taurus-cox1 ACCCCTCTGTTCGTATGATCCGTAATAATTACCGCCGTACTACTACTACTCTCGCTCCCT 600

******** ** * ***** ******** ***************** ***** ** ***

B.taurus.Ch10 **GTACTAGCAGCTGGCATTACAATGCTACTAACAGATCGAAATCTAAATACAACCTTCTTT** 659

B.taurus-cox1 GTATTAGCAGCCGGCATCACAATGCTATTAACAGACCGGAACCTAAATACAACCTTCTTC 660

*** ******* ***** ********* ******* ** ** *****************

B.taurus.Ch10 **GACCCGGCAGGAGGAGGGGACCCTAT**CCTATACCAACACTTATTCTGATTCTTTGGACAC 719

B.taurus-cox1 GACCCGGCAGGAGGAGGAGACCCTATTCTATATCAACACTTATTCTGATTCTTTGGACAC 720

***************** ******** ***** ***************************

B.taurus.Ch10 CCCGAAGTCTATATTCTTATTCTACCTGGGTTTGGAATAATCTCTCATATTGTAACTTAC 779

B.taurus-cox1 CCCGAAGTCTATATTTTAATCTTACCTGGGTTTGGAATAATCTCTCATATCGTGACCTAC 780

*************** * ** **************************** ** ** ***

B.taurus.Ch10 TACTCAGGGAAAAAAGAACCATTCGGATATATAGGAATAGTTTGAGCTATGATATCAATC 839

B.taurus-cox1 TACTCAGGAAAAAAAGAACCATTCGGATATATGGGAATAGTTTGGGCTATAATGTCAATC 840

******** *********************** *********** ***** ** ******

B.taurus.Ch10 AGATTTTTAGGGTTTATCGTATGAGCTCACCACATATTCATGGTCGGAATAGAGGTCGAC 899

B.taurus-cox1 GGATTTCTAGGTTTCATCGTATGAGCCCACCATATATTCACTGTCGGAATAGACGTCGAC 900

***** **** ** *********** ***** ******* *********** ******

B.taurus.Ch10 ACACGGGCCTACATCACATCAGCCACTATGATCACTGCTATCCTAACCGGGGTGAAAGTC 959

B.taurus-cox1 ACACGAGCCTACTTCACATCAGCCACTATAATTATTGCTATTCCAACCGGGGTAAAAGTC 960

***** ****** **************** ** * ****** * ********* ******

B.taurus.Ch10 TTTAGTTGACTAGCAACACTTCACGGAGGTAATATCAAATGATCTCCCGCTATAATGTGA 1019

B.taurus-cox1 TTCAGCTGATTGGCAACACTTCATGGAGGTAATATCAAATGGTCTCCTGCTATAATGTGA 1020

** ** *** * *********** ***************** ***** ************

B.taurus.Ch10 GCCCTGGGCTTCATCTTCCTCTTTACAGTAGGAGGCTTAACTGGAATTGTCCTAGCCAAC 1079

B.taurus-cox1 GCCCTAGGCTTTATTTTCTTATTTACAGTAGGGGGTTTAACTGGAATTGTCTTAGCCAAC 1080

***** ***** ** *** * *********** ** *************** ********

B.taurus.Ch10 TCTTCCCTTGACATTGTTCTCCACGACACATATTATGTTGTCACACACTTCTACTATGTT 1139

B.taurus-cox1 TCTTCCCTCGATATTGTTCTTCACGACACATACTACGTTGTCGCACATTTCCACTATGTT 1140

******** ** ******** *********** ** ****** **** *** ********

B.taurus.Ch10 CTGTCAATAGGAGCTGTATTTGCTATTATAGGAGGATTTGTGCATTGATTCCCACTGTTC 1199

B.taurus-cox1 TTATCAATAGGAGCTGTATTTGCTATTATAGGGGGATTTGTTCATTGATTCCCACTATTC 1200

* ***************************** ******** ************** ***

B.taurus.Ch10 TCAGGT-ATACTCTCA-CGATACATGAGCCAAAATTCACTTTGCAATTATATTCCTTTGG 1257

B.taurus-cox1 TCAGGTTATACTCTCAACGATACATGAGCCAAAATCCACTTCGCAATTATATT--TGTAG 1258

****** ********* ****************** ***** *********** * * *

B.taurus.Ch10 GTGTTAATATGACTTTCTTTCCACAACACTTCTTAGGACTATCTGGCATGCCACGATGAT 1317

B.taurus-cox1 GCGTCAATATAACCTTCTTCCCACAACACTTTCTAGGACTATCTGGCATGCCTCGACGAT 1318

* ** ***** ** ***** *********** ******************* *** ***

B.taurus.Ch10 ACTCCGATTACCCAGACGCATACACAATATGAAATACTGTCTCATCAATAGGCTCATTTA 1377

B.taurus-cox1 ACTCCGACTACCCAGATGCATACACAATATGAAATACTATCTCATCAATAGGCTCATTCA 1378

******* ******** ********************* ******************* *

B.taurus.Ch10 TCTCTCTAACAGCAGTCATGCTAATGGTTTTCATTATCTGAGAAGCATTCACATCCAAGC 1437

B.taurus-cox1 TTTCCCTAACAGCAGTTATACTAATAGTTTTCATCATCTGAGAAGCATTTGCATCTAAAC 1438

* ** *********** ** ***** ******** ************** **** ** *

B.taurus.Ch10 GAGAAGTCTTCACCGTGGATTTAACCACAACAAACCTAGAATGATTAAACGGGTGCCCCC 1497

B.taurus-cox1 GAGAAGTCTTGACTGTAGACTTAACCACGACAAATCTAGAATGATTAAACGGATGCCCTC 1498

********** ** ** ** ******** ***** ***************** ***** *

B.taurus.Ch10 CGCCATACCATACATTTGAAGAACCCACATATGTTAACCTAAAGTAA 1544

B.taurus-cox1 CACCATATCACACATTTGAAGAACCCACCTATGTTAACCTAAAATAA 1545

* ***** ** ***************** ************** ***

**H1 (mouse)**

M.musculus-Ch2 IGSPPPAGSKKVVFRLRSVSSIVMPAASTGSDNRSSTAVISTDQTNSGV*

M.musculus-mt-st IGSPPPAGSKKVVFRLRSVSSIVMPAASTGSDNRSSTAVISTDQTNSGV*

M.musculus-mt-mt IGSPPPAGSKKVVF*LRSVSSMVMPAASTGSDN*SSTAVMSTDQTNSGVW

************** ******:*********** *****:*********

M.musculus-Ch2 YCVMAGGFMLIIVVIKLIAPKIDDTPAK*REKIVRSTDAPAWARFPARGG*

M.musculus-mt-st YCVMAGGFMLIIVVIKLIAPKIDDTPAK*REKIVRSTDAPAWARFPARGG*

M.musculus-mt-mt YCVMAGGFMLMMVVMKLIAPKMDDTPAKW*EKIV*STDAPAWA*FPA*GG*

**********::**:******:****** **** ******** *** **

**H2 (mouse)**

M.musculus-Ch2 ATGTTCATTAATCGTTGATTATTCTCAACCAATCACAAAGATATCGGAACCCTATATCTG 60

M.musculus-cox1 ATGTTCATTAATCGTTGATTATTCTCAACCAATCACAAAGATATCGGAACCCTCTATCTA 60

***************************************************** *****

M.musculus-Ch2 CTATTCGGAGCCTGAGCGGGAATAGTAGGTACTGCACTAAGTATTTTAATTCGAGCAGAA 120

M.musculus-cox1 CTATTCGGAGCCTGAGCGGGAATAGTGGGTACTGCACTAAGTATTTTAATTCGAGCAGAA 120

************************** *********************************

M.musculus-Ch2 TTAGGTCAACCAGGTGCACTTTTAGGAGATGACCAAATTTACAATGTTATCGTAACTGCC 180

M.musculus-cox1 TTAGGTCAACCAGGTGCACTTTTAGGAGATGACCAAATTTACAATGTTATCGTAACTGCC 180

************************************************************

M.musculus-Ch2 CATGTTTTTGTTATAATTTTCTTCATAGTAATACCAATAATAATCGGAGGCTTTGGAAAC 240

M.musculus-cox1 CATGCTTTTGTTATAATTTTCTTCATAGTAATACCAATAATAATTGGAGGCTTTGGAAAC 240

**** *************************************** ***************

M.musculus-Ch2 TGACTTGTCCCACTAATAATCGGAGCCCCAGATATAGCATTCCCACGAATAAATAATATA 300

M.musculus-cox1 TGACTTGTCCCACTAATAATCGGAGCCCCAGATATAGCATTCCCACGAATAAATAATATA 300

************************************************************

M.musculus-Ch2 AGTTTTTGACTCTTACCACCATCATTTCTCCTTCTCCTAGCATCATCAATAGTAGAAGCA 360

M.musculus-cox1 AGTTTTTGACTCCTACCACCATCATTTCTCCTTCTCCTAGCATCATCAATAGTAGAAGCA 360

************ ***********************************************

M.musculus-Ch2 GGAGCAGGAACAGGATGAACAGT**CTACCCACCTCTAGCCGGAAATCTAGCCCATGCAGGA** 420

M.musculus-cox1 GGAGCAGGAACAGGATGAACAGTCTACCCACCTCTAGCCGGAAATCTAGCCCATGCAGGA 420

************************************************************

M.musculus-Ch2 **GCATCAGTAGACCTAACAATTTTCTCCCTTCATTTAGCTGGGGTGTCGTCTATTTTAGGT** 480

M.musculus-cox1 GCATCAGTAGACCTAACAATTTTCTCCCTTCATTTAGCTGGAGTGTCATCTATTTTAGGT 480

***************************************** ***** ************

M.musculus-Ch2 **GCAATTAATTTTATTACCACCATTATCAACATGAAACCTCCAGCCATAACACAATATCAA** 540

M.musculus-cox1 GCAATTAATTTTATTACCACTATTATCAACATGAAACCCCCAGCCATAACACAGTATCAA 540

******************** ***************** ************** ******

M.musculus-Ch2 **ACTCCACTATTTGTCTGATCCGTACTTATTACAGCCGTACTTCTCCTATTATCACTACCA** 600

M.musculus-cox1 ACTCCACTATTTGTCTGATCCGTACTTATTACAGCCGTACTGCTCCTATTATCACTACCA 600

***************************************** ******************

M.musculus-Ch2 **GTACTAGCCGCAGGCATTACTATACTACTAACAGACCGCAACCTAAACACAACTTTCTTT** 660

M.musculus-cox1 GTGCTAGCCGCAGGCATTACTATACTACTAACAGACCGCAACCTAAACACAACTTTCTTT 660

** *********************************************************

M.musculus-Ch2 **GATCCCGCTGGAGGAGGGGACCCAAT**TCTCTACCAACACCTGTTCTGATTCTTTGGACAT 720

M.musculus-cox1 GATCCCGCTGGAGGAGGGGACCCAATTCTCTACCAGCATCTGTTCTGATTCTTTGGGCAC 720

*********************************** ** ***************** **

M.musculus-Ch2 CCAGAAGTTTATATTCTTATCCTCCCAGGATTTGGAATTATTTCACATGTAGTTACTTAC 780

M.musculus-cox1 CCAGAAGTTTATATTCTTATCCTCCCAGGATTTGGAATTATTTCACATGTAGTTACTTAC 780

************************************************************

M.musculus-Ch2 TACTCCGGAAAAAAAGAACCTTTCGGCTATATAGGAATAGTATGAGCAATAATGTCCATT 840

M.musculus-cox1 TACTCCGGAAAAAAAGAACCTTTCGGCTATATAGGAATAGTATGAGCAATAATGTCTATT 840

******************************************************** ***

M.musculus-Ch2 GGCTTCCTAGGCTTTATTGTATGAGCCCACCACATATTCACAGTAGGATTGGATGTAGAC 900

M.musculus-cox1 GGCTTTCTAGGCTTTATTGTATGAGCCCACCACATATTCACAGTAGGATTAGATGTAGAC 900

***** ******************************************** *********

M.musculus-Ch2 ACATGAGCTTACTTTACATCAGCCACTATAATTATCGCAATTCCTACCGGTGTCAAAGTA 960

M.musculus-cox1 ACACGAGCTTACTTTACATCAGCCACTATAATTATCGCAATTCCTACCGGTGTCAAAGTA 960

*** ********************************************************

M.musculus-Ch2 TTTAGCTGACTTGCAACCCTACACGGAGGAAATATTAAATGATCTCCAGCTATACTATGA 1020

M.musculus-cox1 TTTAGCTGACTTGCAACCCTACACGGAGGTAATATTAAATGATCTCCAGCTATACTATGA 1020

***************************** ******************************

M.musculus-Ch2 GCTTTAGGCTTTATTTTCTTATTTACAGTTGGTGGCCTAACCGGAATTGTTCTATCCAAC 1080

M.musculus-cox1 GCCTTAGGCTTTATTTTCTTATTTACAGTTGGTGGTCTAACCGGAATTGTTTTATCCAAC 1080

** ******************************** *************** ********

M.musculus-Ch2 TCATCCCTTGACATCGTGCTTCACGATACATACTATGTAGTAGCCCATTTCCATTATGTT 1140

M.musculus-cox1 TCATCCCTTGACATCGTGCTTCACGATACATACTATGTAGTAGCCCATTTCCACTATGTT 1140

***************************************************** ******

M.musculus-Ch2 CTATCAATGGGAGCAGTGTTTGCTATCATAGCAGGATTCATTCACTGATTCCCATTATTT 1200

M.musculus-cox1 CTATCAATGGGAGCAGTGTTTGCTATCATAGCAGGATTTGTTCACTGATTCCCATTATTT 1200

************************************** ********************

M.musculus-Ch2 TCAGGCTTTACCCTAGATGACACATGAGCAAAAGCCCACTTCGCCATCATGTTCGTAGGG 1260

M.musculus-cox1 TCAGGCTTCACCCTAGATGACACATGAGCAAAAGCCCACTTCGCCATCATATTCGTAGGA 1260

******** ***************************************** ********

M.musculus-Ch2 GTAAACATAACATTCTTCCCTCAACATTTCCTAGGCCTTTCAGGAATACCACGACGCTAC 1320

M.musculus-cox1 GTAAACATAACATTCTTCCCTCAACATTTCCTGGGCCTTTCAGGAATACCACGACGCTAC 1320

******************************** ***************************

M.musculus-Ch2 TCAGACTACCCAGCTGCTTACACTACATGAAACACTGTCTCTTCTATAGGATCATTTATT 1380

M.musculus-cox1 TCAGACTACCCAGATGCTTACACCACATGAAACACTGTCTCTTCTATAGGATCATTTATT 1380

************* ********* ************************************

M.musculus-Ch2 TCACTAACAGCTGTTCTCATCATGATCTTTATAATTTGAGAGGCCTTTGCTTCAAAACGA 1440

M.musculus-cox1 TCACTAACAGCTGTTCTCATCATGATCTTTATAATTTGAGAGGCCTTTGCTTCAAAACGA 1440

************************************************************

M.musculus-Ch2 GAAGTAATATCAGTATCATATGCCTCAACAAATTTAGAATGACTTCATGGCTGCCCTCCA 1500

M.musculus-cox1 GAAGTAATATCAGTATCGTATGCTTCAACAAATTTAGAATGACTTCATGGCTGCCCTCCA 1500

***************** ***** ************************************

M.musculus-Ch2 CCATACCACACATTCGAGGAACCAACCTATGTAAAAGTAAAATAA 1545

M.musculus-cox1 CCATATCACACATTCGAGGAACCAACCTATGTAAAAGTAAAATAA 1545

***** ***************************************
