# Supplementary material for: Probable presence of an ubiquitous cryptic mitochondrial gene on the antisense strand of the cytochrome oxidase I gene
Source: Biol Direct. 2011 Oct 24;6:56. doi: 10.1186/1745-6150-6-56 (PMC3214167; doi:10.1186/1745-6150-6-56)
Supplement: Additional file 2 — Alignments of the EST sequences containing the complete region of Gau translation with homologous regions in mtDNA. With the exception of the Rattus norvegicus sequence, the closest mtDNA issued from a complete genome has been used. Additionally, the nucleotide positions in the genome are given. The sequences corresponding to the gau regions are in bold letters. Characteristics of the sequences are the following: A) EST sequence from Eucalyptus gunnii (Viridiplantae, CT987850.1; another EST sequence (CT980201.1) is strictly identical to this one) and mtDNA sequence from Carica papaya (Viridiplantae, NC_012116); B) EST sequence from Biomphalaria glabrata (Mollusca, EE049639.1; the nucleotide insertion at position 531 is a sequencing artifact because it is not present in all ESTs from the Biomphalaria genus that contain this region) and mtDNA sequence from the same species (NC_005439); C) ESTs sequences from Phlebotomus perniciosus (Insecta, EST1: GW817739.1, EST2: GW816615.1, EST3: GW819720.1; for this last sequence, the 3' end has been removed because it apparently corresponds to a cloning artifact) and mtDNA sequence from Anopheles darlingi (NC_014275); D) ESTs sequences from Mus musculus and Rattus norvegicus (Muridae, BF784456.1 and CO394761.1, respectively) and mtDNA sequence from Mus musculus (NC_006914). [file 1745-6150-6-56-S2.DOC]

A

Eucalyptus.gunnii.EST CGGCCTCTGGATGACCGAAGAACCGAAAGAGATGCTGGTATAAT**ATTGGG** 50

Carica.papaya.mtDNA 191970 ACACCTCTGGATGACCGAAGAACCGAAAGAGATGCTGGTATAAT**ATAGGG**

******************************************* ***

Eucalyptus.gunnii.EST **TCTCCCCCTCCTGCGGGATCAGAAAAGGTTGTATTAAAATTTCGATCGGT** 100

Carica.papaya.mtDNA **TCTCCCCCTCCAGCGGGATCAGAAAAGGTTGTATTAAAGTTTCGATCGGT**

*********** ************************** ***********

Eucalyptus.gunnii.EST **TAATAACATGGTAATTGCCCCTGCCAGTACCGGAAGTGATAATAAAAGTG** 150

Carica.papaya.mtDNA **TAATAACATGGTAATTGCCCCTGCCAGTACCGGGAGTGATAATAAAAGTG**

********************************* ****************

Eucalyptus.gunnii.EST **GGAATGCTGTCACTGGAACGGACCACACAAATAGGGGTGATCTATGCATA** 200

Carica.papaya.mtDNA **GGAATGCTGTCACTAGAACGGACCACACAAATAGGGGTGATCTATGCATA**

************** ***********************************

Eucalyptus.gunnii.EST **GTCATTCCAGGTCCACGCATGTTGGAGATAGTTGTTATAAAATTGATAGA** 250

Carica.papaya.mtDNA **GTCATTCCAGGTCCACGCATGTTGGAGATAGTTGTTATAAAATTGATAGA**

**************************************************

Eucalyptus.gunnii.EST **ACCTAAAATGGATGAAACACCAGATAGATGAAGACTAGAAATTGCTGAAT** 300

Carica.papaya.mtDNA **ACCTAAAATGGATGAAACACCGGATAGATGAAGACTAGAAATTGCTAAAT**

********************* ************************ ***

Eucalyptus.gunnii.EST **CAACTGCTCCTCCAGAATGGCTGGTAATACCACTTAAGGGCGGATAG**ACC 350

Carica.papaya.mtDNA **CAACTGCTCCTCCAGAATGGCTGGTAATACCACTTAAGGGCGGATAG**ACC

**************************************************

Eucalyptus.gunnii.EST GTCCACCCAGTGCCGGTACCCACTTCTACTAAGGCTGGGCTTAATAGGAG 400

Carica.papaya.mtDNA GTCCACCCAGTGCCGCTACCTACTTCTACTAAGGCTGAGCTTAATAGGAG

*************** **** **************** ************

Eucalyptus.gunnii.EST CAAGAGACTTGGTGGCAACAACCAGAATGAAATATTATTTAATCGTGGAA 450

Carica.papaya.mtDNA CAAGAGACTTGGTGGCAACAACCAGAATGAAATATTATTTAATCGTGGAA

**************************************************

Eucalyptus.gunnii.EST ATGCCATGTCAGGTGCACCTATCAGAATCGGAACAGACCAATTACCAGAT 500

Carica.papaya.mtDNA ATGCCATGTCAGGTGCACCTATCAGAATCGGAACAGACCAATTACCAGAT

**************************************************

Eucalyptus.gunnii.EST CCACCTATCATCGCCGGCATAACCATAAAAAAAAAAAAAAAAAA 544

Carica.papaya.mtDNA CCACCTATCATCGCCGGCATAACCATAAAAAAGATCATTAAAAA 192513

******************************** * * *****

B

Biomphalaria.glabrata.EST GGTACACAAAACCAGCAAATAATGCAAATACTGCACCTATAGATAAAACA 50

Biomphalaria.glabrata.mt.DNA 12000 AGTACACAAAACCAGCAAATAATGCAAATACTGCACCTATAGATAAAACA

*************************************************

Biomphalaria.glabrata.EST TAATGAAAATGAGCAACTACATAGTATGTATCATGTAATATAATATCTAA 100

Biomphalaria.glabrata.mt.DNA TAATGAAAATGAGCAACTACATAGTATGTATCATGTAATATAATATCTAA

**************************************************

Biomphalaria.glabrata.EST AGATGAATTAGATAAAACAATTCCTGTTAATCCACCGAGTGTAAATAAAA 150

Biomphalaria.glabrata.mt.DNA AGATGAATTAGATAAAACAATTCCTGTTAATCCACCGAGTGTAAATAAAA

**************************************************

Biomphalaria.glabrata.EST AAATAAAACCTAAAACTCAATATATTGAAGCACTAAAATAACAATGACTT 200

Biomphalaria.glabrata.mt.DNA AAATAAAACCTAAAACTCAATATATTGAAGCACTAAAATAACAATGACTT

**************************************************

Biomphalaria.glabrata.EST CCATATAAAGTTATAAGTCATCTAAAAATTTTAATTCCTGTTGGGACCGC 250

Biomphalaria.glabrata.mt.DNA CCATATAAAGTTATAAGTCATCTAAAAATTTTAATTCCTGTTGGGACCGC

**************************************************

Biomphalaria.glabrata.EST AATAATTATAGTAGCAGCAGTAAAATATGCACGAGTATCAACATCTATTC 300

Biomphalaria.glabrata.mt.DNA AATAATTATAGTAGCAGCAGTAAAATATGCACGAGTATCAACATCTATTC

**************************************************

Biomphalaria.glabrata.EST CAACTGTAAATATATGATGAGCTCATACAATAAATCCTAAAATTCCAATT 350

Biomphalaria.glabrata.mt.DNA CAACTGTAAATATATGATGAGCTCATACAATAAATCCTAAAATTCCAATT

**************************************************

Biomphalaria.glabrata.EST GATACTATAGCATAAATCATACCTAAAGTTCCAAATGCAGGTTTTCTAAC 400

Biomphalaria.glabrata.mt.DNA GATACTATAGCATAAATCATACCTAAAGTTCCAAATGCAGGTTTTCTAAC

**************************************************

Biomphalaria.glabrata.EST AAAATTACTTAAAATATGAGAAACTATTCCAAATCCAGGTAAAATTAAAA 450

Biomphalaria.glabrata.mt.DNA AAAATTACTTAAAATATGAGAAACTATTCCAAATCCAGGTAAAATTAAAA

**************************************************

Biomphalaria.glabrata.EST TATATACTTCTGGATGACCAAAAAACCAAAATAAATGTTGGTATAAA**ATA** 500

Biomphalaria.glabrata.mt.DNA TATATACTTCTGGATGACCAAAAAACCAAAATAAATGTTGGTATAAA**ATA**

**************************************************

Biomphalaria.glabrata.EST **GGATCACCTCCTCCTGCAGGATCAAAAAAAACTAGTATTAAAATTTCGAT** 550

Biomphalaria.glabrata.mt.DNA **GGATCACCTCCTCCTGCAGGATCAAAAAAA**-**CTAGTATTAAAATTTCGAT**

****************************** *******************

Biomphalaria.glabrata.EST **CTGTTAATAACATTGTAATAGCCCCTGCTAAAACAGGTAAAGATAATAAA** 600

Biomphalaria.glabrata.mt.DNA **CTGTTAATAACATTGTAATAGCCCCTGCTAAAACAGGTAAAGATAATAAA**

**************************************************

Biomphalaria.glabrata.EST **AGTAAAAATGCTGTAACTAATACAGATCATACAAATAATGATAAACGTTC** 650

Biomphalaria.glabrata.mt.DNA **AGTAAAAATGCTGTAACTAATACAGATCATACAAATAATGATAAACGTTC**

**************************************************

Biomphalaria.glabrata.EST **CATTGTAATACCGGGAGCACGCATATTAAAAATTGTAGTAATAAAATTAA** 700

Biomphalaria.glabrata.mt.DNA **CATTGTAATACCGGGAGCACGCATATTAAAAATTGTAGTAATAAAATTAA**

**************************************************

Biomphalaria.glabrata.EST **TAGCACCTAAAATTGAACTTATTCCTGCTAAATGTAAAGAAAAAATAGCT** 750

Biomphalaria.glabrata.mt.DNA **TAGCACCTAAAATTGAACTTATTCCTGCTAAATGTAAAGAAAAAATAGCT**

**************************************************

Biomphalaria.glabrata.EST **AAATCCACAGAAGCACCACCATGAGCAATAGGACCACTTAAGGGAGGATA** 800

Biomphalaria.glabrata.mt.DNA **AAATCCACAGAAGCACCACCATGAGCAATAGGACCACTTAAGGGAGGATA**

**************************************************

Biomphalaria.glabrata.EST **T**ACAGTTCAACCTGTACCTACTCCACCCTCAACTATACTAGATACTAACA 850

Biomphalaria.glabrata.mt.DNA **T**ACAGTTCAACCTGTACCTACTCCACCCTCAACTATACTAGATACTAACA

**************************************************

Biomphalaria.glabrata.EST AAAAAAAAAAAAAAAAAAAAAAAAAAA 877 Biomphalaria.glabrata.mt.DNA ATAAAATAAATGAAGGCGGAAGTAATC 10474

* **** *** ** ** **

C

Phlebotomus.perniciosus.EST1 CCATTACGGCCGGGGATAATA--AGTTGATATCCGTGAATTGTTGCAAGT 48

Phlebotomus.perniciosus.EST3 GCATTACGGCCGGGATTCTAG--AGCCGA--------------------- 27

Phlebotomus.perniciosus.EST2 GCATTACGGCCGGGGATTATA--AGTTGATATCCGTGAATTGTTGCAAGT 48

Anopheles.darlingi.mtDNA 2431 AGAATATAGCTGGGCTGTATGTTAATTGAGTACCATGAAGAGTAGCTAAT

* ** ** *** * **

Phlebotomus.perniciosus.EST1 CAACTAAAAATTTTAATTCCTGTAGGAACAGCAATAATTATTGTAGCTGA 98

Phlebotomus.perniciosus.EST3 -------------------------------------------------- 27

Phlebotomus.perniciosus.EST2 CAACTAAAAATTTTAATTCCTGTAGGAACAGCAATAATTATTGTAGCTGA 98

Anopheles.darlingi.mtDNA CAACTAAAAATTTTAATTCCTGTAGGTACGGCAATAATTATAGTAGCAGA

Phlebotomus.perniciosus.EST1 TGTAAAATAAGCTCGAGTATCTACATCTATTCCTACAGTAAATATATGAT 148

Phlebotomus.perniciosus.EST3 -------------------------------------------------- 27

Phlebotomus.perniciosus.EST2 TGTAAAATAAGCTCGAGTATCTACATCTATTCCTACAGTAAATATATGAT 148

Anopheles.darlingi.mtDNA AGTAAAATAAGCTCGTGTATCTACATCTATACCAACAGTAAATATATGAT

Phlebotomus.perniciosus.EST1 GAGCTCAAACAATAAATCCTAAAAGACCAATTGCTAATATAGCATAAATT 198

Phlebotomus.perniciosus.EST3 -------------------------------------------------- 27

Phlebotomus.perniciosus.EST2 GAGCTCAAACAATAAATCCTAAAAGACCAATTGCTAATATAGCATAAATT 198

Anopheles.darlingi.mtDNA GAGCTCATACAATAAATCCTAATAATCCAATGGCTAATATTGCATAAATT 200

Phlebotomus.perniciosus.EST1 ATTCCAAGTGTTCCAAAGGTTTCCTTTTT--TCCTCTTTCATTTCTAATA 246

Phlebotomus.perniciosus.EST3 ------GGCGGCCGAATGTTTCCTTTTTT--TCCTCTTTCATTTCTAATA 69

Phlebotomus.perniciosus.EST2 ATTCCAAGTGTTCCAAAGGTTTCCTTTTTTTTCCTCTTTCATTTCTAATA 248

Anopheles.darlingi.mtDNA ATTCCTAAATTTCCAAAAGTTTCCTTTTT--TCCTCTTTCTTGAGTAATA

* ** ** * ***** ********* * *****

Phlebotomus.perniciosus.EST1 ATATGAGAAATTATTCCAAATCCTGGGAGAATTAAAATATAAACTTCAGG 296

Phlebotomus.perniciosus.EST3 ATATGAGAAATTATTCCAAATCCTGGGAGAATTAAAATATAAACTTCAGG 119

Phlebotomus.perniciosus.EST2 ATATGAGAAATTATTCCAAATCCTGGGAGAATTAAAATATAAACTTCAGG 298

Anopheles.darlingi.mtDNA ATATGTGAAATTATTCCAAATCCTGGTAAAATTAAAATGTAAACTTCAGG

***** ******************** * ********* ***********

Phlebotomus.perniciosus.EST1 ATGTCCAAAAAATCAAAATAAATGTTGATAAAGA**ATAGGATCTCCCCCTC** 346

Phlebotomus.perniciosus.EST3 ATGTCCAAAAAATCAAAATAAATGTTGATAAAGA**ATAGGATCTCCCCCTC** 169

Phlebotomus.perniciosus.EST2 ATGCCCAAAAAATCAAAATAAATGTTGATAAAGA**ATAGGATCTCCCCCTC** 348

Anopheles.darlingi.mtDNA ATGTCCAAAAAATCAAAATAAATGTTGATATAGA**ATAGGATCTCCTCCTC**

*** ************************** ************** ****

Phlebotomus.perniciosus.EST1 **CAGCTGGGTCAAAGAAGGAAGTATTTAAATTACGATCTGTTAAAAGTATT** 396

Phlebotomus.perniciosus.EST3 **CAGCTGGGTCAAAGAAGGAAGTATTTAAATTACGATCTGTTAAAAGTATT** 219

Phlebotomus.perniciosus.EST2 **CAGTTGGGTCAAAGAAGGAAGTATTTAAATTACGATCTGTTAAAAGTATT** 398

Anopheles.darlingi.mtDNA **CGGCTGGATCAAAGAAAGAAGTATTTAAATTTCGGTCTGTTAATAACATA**

* * *** ******** ************** ** ******** * **

Phlebotomus.perniciosus.EST1 **GTAATAGCTCCTGCTAATACAGGTAAAGATAAAAGTAATAAAACAGCTGT** 446

Phlebotomus.perniciosus.EST3 **GTAATAGCTCCTGCTAATACAGGTAAAGATAAAAGTAATAAAACAGCTGT** 269

Phlebotomus.perniciosus.EST2 **GTAATAGCTCCTGCTAATACAGGTAAAGATAAAAGTAATAAAACAGCTGT** 448

Anopheles.darlingi.mtDNA **GTAATAGCTCCAGCTAATACAGGTAAAGATAATAATAATAAAATAGCAGT**

*********** ******************** * ******** *** **

Phlebotomus.perniciosus.EST1 **AATTACTACTGATCAAGCAAATAGAGGTATTCGATCAAGAGTAATTCCTG** 496

Phlebotomus.perniciosus.EST3 **AATTACTACTGATCAAGCAAATAGAGGTATTCGATCAAGAGTAATTCCTG** 319

Phlebotomus.perniciosus.EST2 **AATTACTACTGATCAAGCAAATAGAGGTATTCGATCAAGAGTAATTCCTG** 498

Anopheles.darlingi.mtDNA **AATTACTACTGATCATACAAATAATGGTATTCGATCTAAAGTAATTCCTG**

*************** ****** *********** * ***********

Phlebotomus.perniciosus.EST1 **TTGCTCGTATATTAATAACAGTTGTAATAAAATTTACTGCTCCTAGAATT** 546

Phlebotomus.perniciosus.EST3 **TTGCTCGTATATTAATAACAGTTGTAATAAAATTTACTGCTCCTAGAATC** 369

Phlebotomus.perniciosus.EST2 **TTGCTCGTATATTAATAACAGTTGTAATAAAATTTACTGCTCCTAGAATT** 548

Anopheles.darlingi.mtDNA **GAGATCGTATATTAATTACTGTAGTAATAAAATTTACTGCTCCTAAAATA**

* ************ ** ** ********************** ***

Phlebotomus.perniciosus.EST1 **GATGAAATACCTGCTAAATGGAGTGAGAAAATAGCTAGATCAACTGAGGC** 596

Phlebotomus.perniciosus.EST3 **GATGAAATACCTGCTAAATGGAGTGAGAAAATAGCTAGATCAACTGAGGC** 419

Phlebotomus.perniciosus.EST2 **GATGAAATACCTGCTAAATGGAGTGAGAAAATAGCTAGATCAACTGAGGC** 598

Anopheles.darlingi.mtDNA **GATGAAATTCCTGCTAAATGAAGAGAAAAAATAGCTAAATCAACAGAAGC**

******** *********** ** ** ********** ****** ** **

Phlebotomus.perniciosus.EST1 **TCCTCTATGGGCAATATTTCTTGAAAGAGGAGGATAA**ACAGTTCATCCTG 646

Phlebotomus.perniciosus.EST3 **TCCTCTATGGGCAATACTTCTTGAAAGAGGAGGATAA**ACAGTTCATCCTG 469

Phlebotomus.perniciosus.EST2 **TCCTCTATGGGCAATATTTCTTGAAAGAGGAGGATAA**ACAGTTCATCCTG 648

Anopheles.darlingi.mtDNA **TCCAGCATGAGCAATTCCTGATGATAGAGGAGGATAA**ACAGTTCATCCTG

*** *** ***** * *** *************************

Phlebotomus.perniciosus.EST1 TTCCTGCTCCAGTTTCAACTATTCTTCTAGTTAAAAGAAGAGTTAATGAA 696

Phlebotomus.perniciosus.EST3 TTCCTGCTCCAGTTTCAACTATTCTTCTAGTTAAAAGAAGAGTTAATGAA 519

Phlebotomus.perniciosus.EST2 TTCCTGCTCCAGTTTCAACTATTCTTCTAGTTAAAAGAAGAGTTAATGAA 698

Anopheles.darlingi.mtDNA TTCCAGCTCCATTTTCTACTATACTTCTAGAAATTAATAAAGTTAAAGAA

**** ****** **** ***** ******* * * * ****** ***

Phlebotomus.perniciosus.EST1 GGAGGAAGTAATCAAAATCTTATATTATTTATTCGTGGAAATGCTATATC 746

Phlebotomus.perniciosus.EST3 GGAGGAAGTAATCAAAATCTTATATTATTTATTCGTGGAAATGCTATATC 569

Phlebotomus.perniciosus.EST2 GGAGGAAGTAATCAAAATCTTATATTATTTATTCGTGGAAATGCTATATC 748

Anopheles.darlingi.mtDNA GGGGGAAGTATTCAAAAACTTATATTGTTTATTCGTGGGAAAGCCATATC

** ******* ****** ******** *********** ** ** *****

Phlebotomus.perniciosus.EST1 AGGGGCTCCTAGTATTAAAGGAACAAGTCAATTTCCAAATCCTCCAATTA 796

Phlebotomus.perniciosus.EST3 AGGGGCTCCTAGTATTAAAGGAACAAGTCAATTTCCAAATCCTCCAATTA 619

Phlebotomus.perniciosus.EST2 AGGGGCTCCTAGTATTAAAGGAACAAGTCAATTTCCAAATCCTCCAATTA 798

Anopheles.darlingi.mtDNA AGGAGCTCCTAATATTAAAGGCACTAATCAATTACCAAATCCTCCAATTA

*** ******* ********* ** * ****** ****************

Phlebotomus.perniciosus.EST1 TAATAGGTATAACTATAAAGAAAATTATAACAAAGGCATGGGCTGTTACA 846

Phlebotomus.perniciosus.EST3 TAATAGGTATAACTATAAAGAAAATTATAACAAAGGCATGGGCTGTTACA 669

Phlebotomus.perniciosus.EST2 TAATAGGTATAACTATAAAGAAAATTATAACAAAGGCATGGGCTGTTACA 848

Anopheles.darlingi.mtDNA TAATTGGTATTACTATAAAAAAAATTATAATAAATGCATGTGCTGTAACA

**** ***** ******** ********** *** ***** ***** ***

Phlebotomus.perniciosus.EST1 ATTACATTATAAATTTGATCATCTCCAATTAGGGCTCCGGGATGACCAAG 896

Phlebotomus.perniciosus.EST3 ATTACATTATAAATTTGATCATCTCCAATTAGGGCTCCGGGATGACCAAG 719

Phlebotomus.perniciosus.EST2 ATTACATTATAAATTTGATCATCTCCAATTAGGGCTCCGGGATGACCAAG 898

Anopheles.darlingi.mtDNA ATTACATTATAAATTTGATCATCACCAATAAATGCTCCAGGATGACCTAA

*********************** ***** * ***** ******** *

Phlebotomus.perniciosus.EST1 TTCTGCTCGAATTAAAATTCTTAATGAAGTTCCAACTATTCCTGCTCANG 946

Phlebotomus.perniciosus.EST3 TTCTGCTCGAATTAAAATTCTTAATGAAGTTCCCACTATTCCTGCTCAAG 769

Phlebotomus.perniciosus.EST2 TTCTGCTCGAAT-AAAATTCTTAATGAAGTGCAAAAAAAAAAAAAA---- 943

Anopheles.darlingi.mtDNA TTCAGCCCGAATTAAAATTCTTAATGAAGTTCCAACTATTCCAGCTCAAG

*** ** ***** ***************** * *

Phlebotomus.perniciosus.EST1 CTCCGAANAAAAAAAAAAAAAAAAAAAAAAAAAAATTGGG 986

Phlebotomus.perniciosus.EST3 CTCCAAAAAAAAAAAAAAAAAAAAAAAAAAAAAATGTGCG 809

Phlebotomus.perniciosus.EST2 ---------------------------------------- 943

Anopheles.darlingi.mtDNA CTCCAAAAATAAAATATAATGTTCCAATATCCTTATGATT 1446

D

Mus.musculus.EST --------------------------------------------------

Mus.musculus.mtDNA 6321 TATTACCTCCGTGTAGGGTTGCAAGTCAGCTAAATACTTTGACACCGGTA

Rattus.norvegicus.EST CGGATTCCCGGGATTTTTTTTTGAGTCAGCTGAATACTTTTACGCCTGTA 50

Mus.musculus.EST --------------------------------------------------

Mus.musculus.mtDNA GGAATTGCGATAATTATAGTGGCTGATGTAAAGTAAGCTCGTGTGTCTAC

Rattus.norvegicus.EST GGAATTGCGATAATTATAGTGGCAGATGTAAAGTAGGCTCGGGTGTCTAC 100

Mus.musculus.EST -------------GTTCTGATTCTTTGGGCACCCAGAACAGATGCTGG-- 35

Mus.musculus.mtDNA ATCTAATCCTACTGTGAATATGTGGTGGGCTCATACAATAAAGCCTAGAA

Rattus.norvegicus.EST ATCTAGGCCTACTGTGAATATGTGATGTGCTCATACAATAAATCCTAGGA 150

** ** ** ** * * ** * * ** *

Mus.musculus.EST -----------------------------------TAGAGAATTGGGTCC 50

Mus.musculus.mtDNA AGCCAATAGACATTATTGCTCATACTATTCCTATATAGCCGAAAGGTTCT

Rattus.norvegicus.EST AGCCAATAGATATTATGGCTCATACCATACCTATATATCCGAAGGGTTCT 200

** * ** **

Mus.musculus.EST CCTCCTCCAGCGGGATCAAATTCTCTACCAGCATCTGTTCTGATTC---- 96

Mus.musculus.mtDNA TTTTTTCCGGAGTAGTAAGTAACTACATGTGAAATAATTCCAAATCCTGG

Rattus.norvegicus.EST TTTTTTCCAGAGTAATAGGTAACTACATGTGAAATAATTCCAAACCCTGG 250

* *** * * * ** * * * *** * *

Mus.musculus.EST ------------------TTCTGGGTGCCCAAAGAATCAGAACAGATGCT 128

Mus.musculus.mtDNA GAGGATAAGAATATAAACTTCTGGGTGCCCAAAGAATCAGAACAGATGCT

Rattus.norvegicus.EST AAGAATTAAGATGTACACTTCTGGGTGGCCGAAGAATCAGAATAGGTGTT 300

********* ** *********** ** ** *

Mus.musculus.EST GGTAGAGA**ATTGGGTCCCCTCCTCCAGCGGGATCAAAGAAAGTTGTGTTT** 178

Mus.musculus.mtDNA GGTAGAGA**ATTGGGTCCCCTCCTCCAGCGGGATCAAAGAAAGTTGTGTTT**

Rattus.norvegicus.EST GATAAAGG**ATTGGGTCTCCACCTCCAGCGGGGTCGAAGAAAGTAGTATTT** 350

* ** ** ******** ** *********** ** ******** ** ***

Mus.musculus.EST **AGGTTGCGGTCTGTTAGTAGTATAGTAATGCCTGCGGCTAGCACTGGTAG** 228

Mus.musculus.mtDNA **AGGTTGCGGTCTGTTAGTAGTATAGTAATGCCTGCGGCTAGCACTGGTAG**

Rattus.norvegicus.EST **AGATTTCGGTCTGTAAGGAGTATAGTGATACCTGCTGCTAATACTGGCAG** 400

** ** ******** ** ******** ** ***** **** ***** **

Mus.musculus.EST **TGATAATAGGAGCAGTACGGCTGTAATAAGTACGGATCAGACAAATAGTG** 278

Mus.musculus.mtDNA **TGATAATAGGAGCAGTACGGCTGTAATAAGTACGGATCAGACAAATAGTG**

Rattus.norvegicus.EST **TGAGAGAAGTAGTAGGACGGCTGTAATTAGTACGGATCATACAAAGAGAG** 450

*** * ** ** ** *********** *********** ***** ** *

Mus.musculus.EST **GAGTTTGATACTGTGTTATGGCTGGGGGTTTCATGTTGATAATAGTGGTA** 328

Mus.musculus.mtDNA **GAGTTTGATACTGTGTTATGGCTGGGGGTTTCATGTTGATAATAGTGGTA**

Rattus.norvegicus.EST **GTGTCTGATATTGGGTTATAGCAGGGGGTTTTATATTAATGATAGTGGTG** 500

* ** ***** ** ***** ** ******** ** ** ** ********

Mus.musculus.EST **ATAAAATTAATTGCACCTAAAATAGATGACACTCCAGCTAAATGAAGGGA** 378

Mus.musculus.mtDNA **ATAAAATTAATTGCACCTAAAATAGATGACACTCCAGCTAAATGAAGGGA**

Rattus.norvegicus.EST **ATAAAGTTGATAGCTCCTAAGATAGAAGACACCCCGGCTAGGTGGAGGGA** 550

***** ** ** ** ***** ***** ***** ** **** ** *****

Mus.musculus.EST **GAAAATTGTTAGGTCTACTGATGCTCCTGCATGGGCTAGATTTCCGGCTA** 428

Mus.musculus.mtDNA **GAAAATTGTTAGGTCTACTGATGCTCCTGCATGGGCTAGATTTCCGGCTA**

Rattus.norvegicus.EST **AAAAATAGTTAAATCTACGGATGCTCCAGCATGGGCTAGGTTTCCGGCTA** 600

***** **** ***** ******** *********** **********

Mus.musculus.EST **GAGGTGGGTAGACTGTTCATCCTG**TTCCTGCTCCTGCTTCTACTATTGAT 478

Mus.musculus.mtDNA **GAGGTGGGTAGACTGTTCATCCTG**TTCCTGCTCCTGCTTCTACTATTGAT

Rattus.norvegicus.EST **AGGGGGGATATACTGTTCATCCTG**TTCCAGCTCC-GCTTCTACTATGGAG 649

** ** ** ***************** ***** *********** **

Mus.musculus.EST GATGCTAGGAGAAGGAGAAATGATGGTGGTAGGAGTCAAAAACTTATATT 528

Mus.musculus.mtDNA GATGCTAGGAGAAGGAGAAATGATGGTGGTAGGAGTCAAAAACTTATATT

Rattus.norvegicus.EST GATGCTAAAGGAGTAGA-----ATGATGGAGGAAGCATCAAAGCTATGTT 694

******* ** *** *** * ** *** *** **

Mus.musculus.EST ATTTATTCGTGGGAATGCTATATCTGGGGCTCCGATTATTAGTGGGACAA 578

Mus.musculus.mtDNA ATTTATTCGTGGGAATGCTATATCTGGGGCTCCGATTATTAGTGGGACAA

Rattus.norvegicus.EST ATTATCGTGGGATGCTAATCAGGGCTCATTATAGTGTACAGTCAGTCCGA 744

*** * * * * * ** * * *

Mus.musculus.EST GTCAGTTTCCAACTAGTTCTAGATACGC---------------------- 606

Mus.musculus.mtDNA GTCAGTTTCCAAAGCCTCCAATTATTATTGGTATTACTATGAAGAAAATT

Rattus.norvegicus.EST GCTNCATATATAGTATACATAGAAATATAGATGCTGGCGGACAGACTAAA 794

* * * *

Mus.musculus.EST ------------------------------------- 606

Mus.musculus.mtDNA ATAACAAAAGCATGGGCAGTTACGATAACATTGTAAA 5485

Rattus.norvegicus.EST TTGCTCTCAATGCGCGTGCTATCATCATAAACTACTC 831
